# Novel covalent and non-covalent complex-based pharmacophore models of SARS-CoV-2 main protease (M^pro^) elucidated by microsecond MD simulations

**DOI:** 10.1038/s41598-022-17204-0

**Published:** 2022-08-18

**Authors:** Yasser Hayek-Orduz, Andrés Felipe Vásquez, María Francisca Villegas-Torres, Paola A. Caicedo, Luke E. K. Achenie, Andrés Fernando González Barrios

**Affiliations:** 1 Grupo de Diseño de Productos y Procesos (GDPP), Department of Chemical and Food Engineering, Universidad de los Andes, Bogotá, Colombia; 2 Naturalius SAS, Bogotá, Colombia; 3 Centro de Investigaciones Microbiológicas (CIMIC), Department of Biological Sciences, Universidad de los Andes, Bogotá, Colombia; 4 Grupo Natura, Faculty of Sciences, Universidad ICESI, Cali, Colombia; 5 Department of Chemical Engineering, Polytechnic Institute and State University from Virginia (Virginia Tech), Blacksburg, USA

**Keywords:** Structure-based drug design, Computational chemistry, Computational chemistry, Viral infection, Molecular dynamics

## Abstract

As the world enters its second year of the pandemic caused by SARS-CoV-2, intense efforts have been directed to develop an effective diagnosis, prevention, and treatment strategies. One promising drug target to design COVID-19 treatments is the SARS-CoV-2 M^pro^. To date, a comparative understanding of M^pro^ dynamic stereoelectronic interactions with either covalent or non-covalent inhibitors (depending on their interaction with a pocket called S1’ or oxyanion hole) has not been still achieved. In this study, we seek to fill this knowledge gap using a cascade in silico protocol of docking, molecular dynamics simulations, and MM/PBSA in order to elucidate pharmacophore models for both types of inhibitors. After docking and MD analysis, a set of complex-based pharmacophore models was elucidated for covalent and non-covalent categories making use of the residue bonding point feature. The highest ranked models exhibited ROC-AUC values of 0.93 and 0.73, respectively for each category. Interestingly, we observed that the active site region of M^pro^ protein–ligand complex undergoes large conformational changes, especially within the S2 and S4 subsites. The results reported in this article may be helpful in virtual screening (VS) campaigns to guide the design and discovery of novel small-molecule therapeutic agents against SARS-CoV-2 M^pro^ protein.

## Introduction

Since the outbreak of the novel coronavirus disease (COVID-19) in December 2019, our entire civilization has been facing a serious and challenging public health threat^1,2^. This has been demonstrated by almost 250 million cases and more than 5 million deaths around the globe^3^, especially throughout several epidemiological peaks, which has unleashed an unprecedented impact on healthcare systems, economies, and society^2^. The causative agent of this pandemic illness is a positive-sense, single-stranded RNA (+ ssRNA) virus referred to as SARS-CoV-2 (severe acute respiratory syndrome coronavirus 2) because of its similarity to the already known SARS-CoV in terms of their genome sequence (82% identical), transmission mode, and clinical manifestations^4,5^. Whereas significant progress has been made to generate pre-infection treatments such as vaccines, the implementation of drug discovery strategies to create post-infection therapies is still needed. In this field, a limited number of molecules has been developed up to date and those under study still need rigorous and valid assessment on humans^6,7^. Therefore, post-infection treatment alternatives able to block the replication and activity of SARS-CoV-2 with an optimal selectivity, efficacy, and safety profile, are being urgently sought.

**Figure 1:**
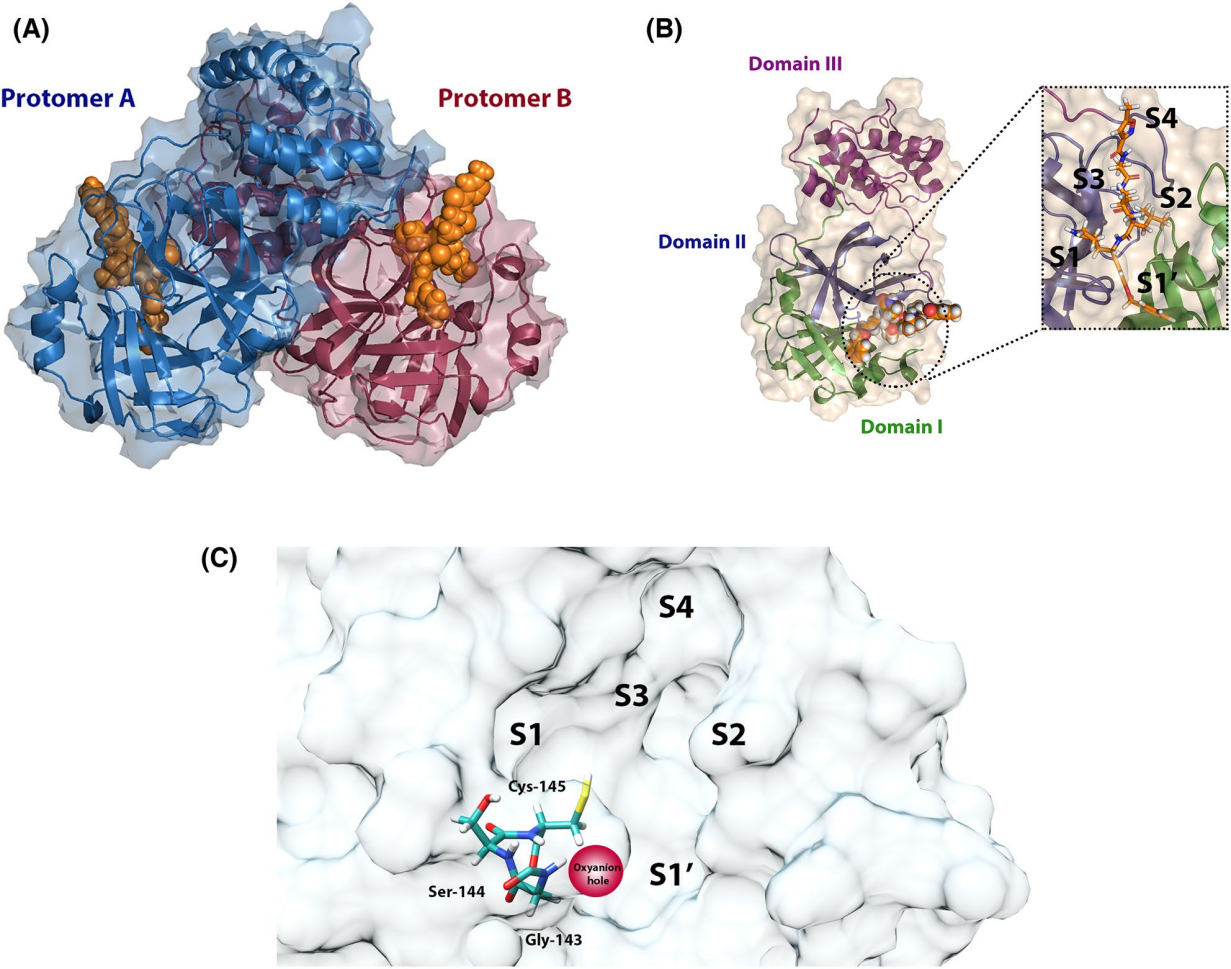
(**A**) Crystal structure of SARS-CoV-2 M^pro^ dimer. (**B**) Crystal structure of SARS-CoV-2 M^pro^ monomer. (**C**) Active site of SARS-CoV-2 M^pro^ with subsites and oxyanion hole (Cys-145, Ser-144, and Gly-143) highlighted. (PDB entry: 6LU7).

One of the most promising proteins to design COVID-19 treatments is the SARS-CoV-2 main protease (M^pro^). The role of this protein is crucial for the proteolysis process, which is an essential step required for viral RNA replication^8,9^. Coronavirus main protease has been found to exist as a dimer in aqueous solutions and protein crystals^10,11^ (Fig. 1A). The SARS-CoV-2 M^pro^ monomer is composed of three domains and its active site has 5 subsites labeled using the Schechter and Berger nomenclature (Fig. 1B). The proteolysis reaction takes place on the S1' subsite, where Cys-145 is responsible for reacting as a nucleophile with the substrate. Particularly, Cys-145 is located on an active site area called oxyanion hole composed of residues Gly-143, Ser-144, and Cys-145^12^ (Fig. 1C). To catalyze proteolysis reaction, the protease uses the oxyanion hole to donate hydrogen bonds (HB) to the substrate via N–H groups. Depending on the chemical group that is located on the oxyanion hole, the main protease inhibitors can be categorized into two types: covalent inhibitors and non-covalent inhibitors (Figure S1). Covalent inhibitors are molecules with electrophilic groups that are able to covalently bind to the active site. Contrarily, the non-covalent inhibitors do not have reactive fragments and only inhibit through non-covalent interactions. However, and although there are studies on the dynamic behavior or the steric and electronic features (pharmacophore modeling) of covalent and non-covalent inhibitors in complex with main protease^13,14^, the differences in behavior between the two categories in virtual screening processes are not yet known, and to date pharmacophore modeling studies have been carried out but no taking into account the active site flexibility from MD simulations^15^. On the other hand, a relevant number of potent inhibitors are currently known with experimental evidence but lacking crystallographic pose. Taking advantage of the information that these inhibitors can provide is key to guiding the design of future agents with activity against SARS-CoV-2.

**Figure 2:**
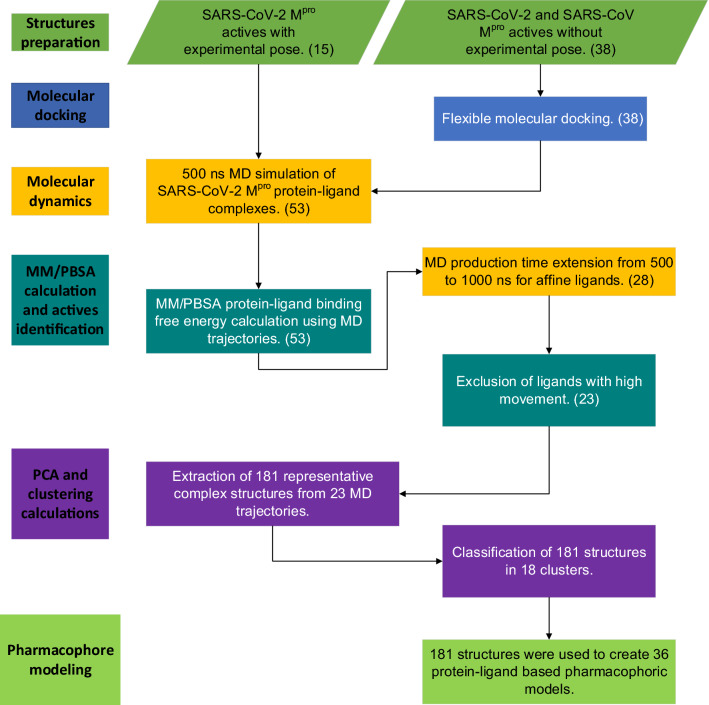
Summary of the methods to generate pharmacophore models for SARS-CoV-2 M^pro^. We collected 15 inhibitors with protein–ligand crystal available and 38 inhibitors with protein–ligand crystal unavailable. These 38 inhibitors went through a molecular docking process to predict ligand poses on the active site. The 38 predicted poses and the 15 experimental poses were taken to perform a MD simulation with 500 ns production time. The MD trajectories were used for MM/PBSA free energy calculations and 28 systems with protein–ligand binding free energy less than − 100 kJ/mol were identified and the respective ligands were considered as possible actives. The MD production time of these 28 systems was extended to 1000 ns. Ligands showing prominent movement on the active site were excluded resulting in 23 actives. The 23 MD trajectories were used for a PCA and clustering process in order to extract representative conformations of each ligand on the active site. Finally, the conformations were employed to create protein–ligand-based pharmacophore models.

In this research work, with the aim of modeling, identifying, and discriminating the essential physicochemical features that favor the interaction between SARS-CoV-2 M^pro^ and either covalent and non-covalent inhibitors, we designed novel complex-based pharmacophore models, using a cascade protocol of docking, molecular dynamics (MD), clustering, and Molecular Mechanics Poisson-Boltzmann Surface Area (MM/PBSA) free energy calculations (Fig. 2). The protocol started with SARS-CoV-2 complex-based MD simulations, which resulting trajectories were combined with MM/PBSA calculations to estimate the binding free energy of each protein–ligand complex and generate an overall ranking. Finally, the top-ranked protein–ligand complexes were subsequently used to design pharmacophoric models able to consider active site flexibility using principal component analysis (PCA) and k-means clustering. It is noteworthy that some models make use of the electrophilic group feature also called as residue bonding point feature, which is a pharmacophoric feature rarely found in the literature. The pharmacophore models generated together with the information reported will be useful when conducting future studies focused on the design of new drugs that can function as a treatment for COVID-19.

## Methods

### SARS-CoV-2 M^pro^ flexibility study using active site cavity size

Atomic coordinates of SARS-CoV-2 M^pro^ (PDB 6YB7) were uploaded to the Computed Atlas of Surface Topography of proteins (CASTp) web server and enzyme cavities were calculated with a radius of 1.4 Å^16^. The 3D apoprotein structures 6M03, 6M2Q, 6WQF, 6WTM, 6Y2E, 6Y84, 6YB7, 7K3T and the 3D protein complex structures 6LU7, 6LZE, 6M0K, 6WNP, 6WTJ, 6WTK, 6WTT, and 7BUY were downloaded. Residues located at 5 Å to the ligand for 6LU7 crystal were found, and these residues were used to calculate active site cavity volume and area through the tool SURFNET of the software UCSF Chimera with a grid interval of 1.4 and a cutoff of 12^17^. Specific results about cavity calculations can be found in Supplementary Information, SARS-CoV-2 M^pro^ active site flexibility study.

### Preparation of SARS-CoV-2 M^pro^ structures

A total of 15 3D protein complex structures of SARS-CoV-2 M^pro^ were downloaded: 6LU7, 6LZE, 6M0K, 6M2N, 6W63, 6WTT, 6XMK, 6XR3, 6Y2F, 7C8R, 7C8T, 7JU7, 7JYC, 7K40, and 7K6D (Table S1). Hydrogens were added with EPIK module at a pH of 7.2 ± 0.2 similar to cytosol pH^18^ (Schrödinger, LCC). Some histidine residues were assigned with the following protonation states: HID-41, HIE-163, HIE-164, and HIP-172 based on information from literature^12,19^. The remaining residues were optimized with PROPKA at a pH of 7.2. A conserved water molecule near to His-41 was preserved in all structures^20^. Protein structures with missing atoms were treated with Swiss-Model and EPIK^21^ (Schrödinger, LCC).

### Flexible molecular docking

Molecules with inhibitory activity lacking crystallographic pose as of October 15^th^, 2020 towards SARS-CoV-2 and SARS-CoV M^pro^ were labeled with identification numbers from 1 to 38. The structures of these molecules are available in Table S2 and Table S3. The molecules were prepared for a molecular docking procedure using the tool Ligand Preparation (Schrödinger, LCC). The force field OPLS3 and a tautomer generation at a 7.2 ± 0.2 pH were used. To perform flexible molecular docking, a choice of flexible residues must be carried out. For that choice, the crystals 6YB7, 6M2Q, 6M03, 6Y2E, 7BRO, 6LZE, 5RGP, 6LU7, and 6Y2F were used. To choose flexible residues, we created rules based on literature information^22^. The rules are as follows: the residue must (1) be located in a loop, (2) have hydrophobic or hydrophilic contacts with the ligand, (3) have rotamers in its side chain that can exist with a probability close to 0.7, and (4) show movement in several protein crystals. More specific details of this procedure can be found in Supplementary Information, Flexible molecular docking.

The Induced fit docking tool was used to perform flexible molecular docking (Schrödinger, LCC). The receptor used was 6LU7 protein crystal and an energy restrained minimization on this structure was executed with 0.18 Å RMSD. The box was located manually in the active site center using a centroid composed of the residues: Thr-26, Ser-46, Asn-142, Met-165, and Pro-168. Ring conformations were sampled with an energy window of 2.5 kcal/mol. The flexible residues Met-49, Asn-142, Cys-145, Met-165, Asp-187, and Gln-189 were specified with the Trim side chains option. The poses amount was limited to 20 poses per ligand and the flexible residues located at 5 Å of the ligand were energetically minimized. The XP scoring function was used. The ligand poses were refined through MM/GBSA calculations using the Prime MM/GBSA module (Schrödinger, LCC). The solvation model was VSGB, the force field was OPLS3 and the residues located at 5 Å of the ligand pose were minimized. The XP Gscore and ΔG MM/GBSA values of the best poses of each ligand can be found in Table S8 and Table S9.

### Molecular dynamics simulations

Experimental ligand poses contained in the treated structures from the Preparation of SARS-CoV-2 M^pro^ structures section (Table S1) and predicted poses from the “Flexible molecular docking” section (Table S2, S3) were extracted. These ligand structures were used for a quantum mechanics electronic energy minimization using RHF/6-31G(d) model chemistry. The minimized geometries were utilized for a quantum mechanics restrained electrostatic potential calculation (RESP) through a Merz-Singh–Kollman (MK) scheme. All quantum mechanics calculations were performed in the gas phase, with a singlet multiplicity and using Gaussian 16. The RESP calculation was employed for ligand parameterization using the GAFF force field through AmberTools20. Considering SARS-CoV-2 M^pro^ is a dimeric protein, then, one ligand was situated on each protomer and all MD simulations were carried out with the protein in the dimeric form. The protein atoms were parametrized using the ff14SB force field and ligand atoms with GAFF force field^23^. Additionally, the acpype.py script was used to convert files to Gromacs format^24^.

Protein–ligand complexes were solvated using a cubic box with a distance of 1.5 nm. Water molecules were parametrized using the TIP3P model. System charge was neutralized using sodium and chlorine ions with a NaCl concentration of 0.15 M. The system preparation was performed through three steps: energy minimization, heating-up, and equilibration. Energy minimization was accomplished using the steepest descent algorithm with a 0.01 nm step. Heating-up was carried out with the Berendsen thermostat, at a temperature of 310 K and a simulation time of 100 ps. Initial random atomic velocities were obtained using the Maxwell–Boltzmann distribution with a temperature of 310 K. On the other hand, system equilibration was executed with a 100 ps simulation time using the Berendsen thermostat and Berendsen barostat. The heating-up and equilibration were performed using restraints of 1000 kJ/(mol nm^2^) for backbone and ligand atoms. Electrostatic interactions were modeled with the Particle Mesh Ewald (PME) method using a Fourier spacing of 0.125 and a 1 nm cutoff. Heating-up, equilibration, and production were executed with a 2 fs time step using Gromacs 2020.2. Finally, the production run for the systems was performed using a 500 ns simulation time with the Berendsen thermostat and Parrinello-Rahman barostat. Some productions were extended to 1000 ns (more specific details of this procedure can be found in the “MM/PBSA free energy calculations” section). All simulations were run in Virginia Tech's Advanced Research Computing (ARC) Cluster and MAGNUS cluster from Universidad de Los Andes. Specific details about MD simulations stability can be found in Supplementary Information, Molecular dynamics stability. For PCA analysis of dimer movements the pyPcazip toolkit was utilized^25^. The PDB files corresponding to the movies of the principal component motions can be found in Supplementary Data 1.

### MM/PBSA free energy calculations

For MM/PBSA free energy calculations, the tool g_mmpbsa was used in conjunction with the 500 ns MD trajectories of 53 protein–ligand complexes^26^. Sodium chloride concentration was defined with a value of 0.15 M. Protein dielectric constant and solvent dielectric constant were established as 2 and 80 respectively^26^. For all calculations, the non-linear version of the Poisson-Boltzmann equation and the SASA model were used. The MM/PBSA calculation was performed every 50 MD trajectory frame. MM/PBSA energy calculations were used to create a ligand affinity ranking. A scheme of the process to generate the ranking can be found in Figure S6. Since SARS-CoV-2 M^pro^ was simulated in its dimer form then each protomer was in complex with a ligand, therefore, we obtained 2 protein–ligand binding free energy values for each simulated system. We found that systems which presented a low variation coefficient for binding free energy usually showed a lower ligand movement or a stronger interaction (lower delta free energy). Thus, variation coefficient for the two protein–ligand binding energies corresponding to each protomer was calculated and the protomer with the lower value of variation coefficient was chosen for the ranking. The average of protein–ligand binding energies and standard deviation were obtained for both protomers using MD simulation frames with the MmPbSaSat.py and MmPbSaDecomp.py python scripts from g_mmpbsa web page (Table S10, Table S11, and Table S12)^26^.

Consecutively, the standard deviation of each protein–ligand binding energy data was added to the average to create data lacking standard deviation. These lacking standard deviation values were used to create a first ligand affinity ranking where complexes that presented the lower values of protein–ligand binding energy were located at the top positions (Table S13). Ligands that presented protein–ligand binding energy lower than − 100 kJ/mol (− 24 kcal/mol) were considered as possible active ligands. To choose this cut-off, the approximated relationship ($\Delta \mathrm{G}=\mathrm{RTln}({\mathrm{IC}}_{50})$) between delta free energy ($\Delta \mathrm{G}$) and half maximal inhibitory concentration ${\mathrm{IC}}_{50}$ was used^27^. For an inhibitor with an ${\mathrm{IC}}_{50}$ value in the nanomolar range then $\Delta \mathrm{G}$ is approximately − 50 kJ/mol for 310 K, being that the MM/PBSA method overestimates free energy values^28^, we decided to use − 100 kJ/mol as a cut-off.

MD trajectories of active ligands were extended to 1000 ns. The 1000 ns MD trajectories were used for new MM/PBSA protein–ligand binding energy calculations (Table S14) and a second affinity ranking for active ligands was created (Table S15). Among the 28 MD simulations, we selected 23 protein–ligand complexes that showed a low movement of the ligand, based on RMSD values and visual inspection of the MD production stage (Supplementary Data 1).

### Principal component analysis and clustering of MD trajectories

To extract ligand poses of complexes from Table S15 using MD trajectories, a principal component analysis, and a clustering procedure was carried out. We decided to combine PCA with k-means clustering by virtue of dimensionality reduction allows to reduce noise and generate more compact and well-separated clusters than k-means clustering alone^29^. Furthermore, since the principal components with the highest eigenvalue are those that contain the majority of variability, then the noise generated by atom movements that are not relevant is reduced. The ligand atomic coordinates of the protomer corresponding to the lower value of protein–ligand MM/PBSA binding energy variation coefficient were extracted from the MD simulation. The hydrogen ligand atoms were removed and the remaining ligand atomic coordinates of each MD trajectory frame were employed in a principal component analysis (PCA) calculation using Bio3D software^30^. The three components with the highest eigenvalue were used with the K-means clustering method to separate the ligand poses for each MD simulation frame in ligand-pose-clusters (LPC). The optimal number of LPC was defined using the SSR/SST ratio value^29^. For every cluster, a centroid was calculated and the nearest MD simulation frame to the centroid was considered as the representative ligand pose of that cluster. The representative structures were analyzed through LigPlot+ software^31^ and ligand–protein interaction diagrams with interaction frequency percentages were generated. Files of representative structures in PDB format are available in Supplementary Data 2.

The representative protein–ligand complex structure corresponding to each LPC was used for a second principal component analysis (PCA) calculation using the atomic coordinates of active site alpha carbons. The three components with the highest eigenvalue were used with the K-means clustering method to separate the protein–ligand complex structures in active-site-conformation-clusters (ASCC) and the optimal number of clusters was defined using the SSR/SST ratio value^29^. A scheme of this procedure can be found in Figure S7.

### Pharmacophore modeling

The clustering procedure preceding pharmacophore modeling is vital for this receptor, because it allows taking account the active site flexibility represented by exclusion volume locations. Protein–ligand complex representative structures corresponding to each LPC were loaded into LigandScout Expert version and complex-based pharmacophore models were created. The pharmacophore models were separated into groups (each one corresponding to one ASCC). The pharmacophore models contained in the correspondent cluster were aligned using reference points (Figure S47). Next, the aligned structures were merged with LigandScout options: merge pharmacophores and interpolate overlapping features (Figure S48, S49). The features with a low occurrence frequency or low contribution to binding energy were configured as optional features. One pharmacophoric feature, hydrogen bond acceptor (HBA), near the oxyanion hole was changed to the residue bonding point feature. Thereby, a new set of ligand–protein-based pharmacophore models were generated (Figure S50). Models which have the HBA feature located on the oxyanion hole are named non-covalent models and models which have the residue bonding point feature located on the oxyanion hole are named covalent models.

Next, the 23 ligands considered active were used to create two groups: non-covalent ligands which correspond to all 23 ligands, and covalent ligands which correspond to 19 ligands. To perform the validation of the pharmacophore models, a decoys library was generated using the non-covalent ligands and the covalent ligands. This library was developed making use of RADER (Rapid Decoy Retriever) capable of generating decoys from active molecules^32^. The library used for the compound search was ChEMBL. The RADER settings were left at their default value with the difference of the Maximum decoys per active ligand and Minimum decoys per active ligand parameters. These parameters were established at the value of 100. The library conformers were generated with the default values ​​of the iCon Best option. The decoys coming from the non-covalent ligands are called non-covalent decoys and decoys coming from the covalent ligands are called covalent decoys.

The calculation validation process was divided into two parts, the first consists of modifying the feature tolerances of the pharmacophore models. For vector-related features a 3.3 Å tolerance (hydrogen bonding features) and for non-vector-related features (residue point and hydrophobic feature) a 4.35 Å tolerance were employed. The 5^th^ pharmacophore models that presented the best performance measured by ROC-AUC (Area under ROC curve) and BACC (balanced-accuracy) were selected for the second part. In the second stage, these pharmacophore models were refined decreasing tolerance and changing weight values with which it was expected to improve the selectivity of the models. In addition, the maximum number of pharmacophores omitted was varied to find the optimal settings for each model. Finally, we used Nirmatrelvir, one FDA-approved SARS-CoV-2 M^pro^ inhibitor designed by Pfizer, as a positive control of pharmacophore models. Models files in PMZ format are available in Supplementary Data 2.

## Results

### Molecular dynamics simulations

**Figure 3:**
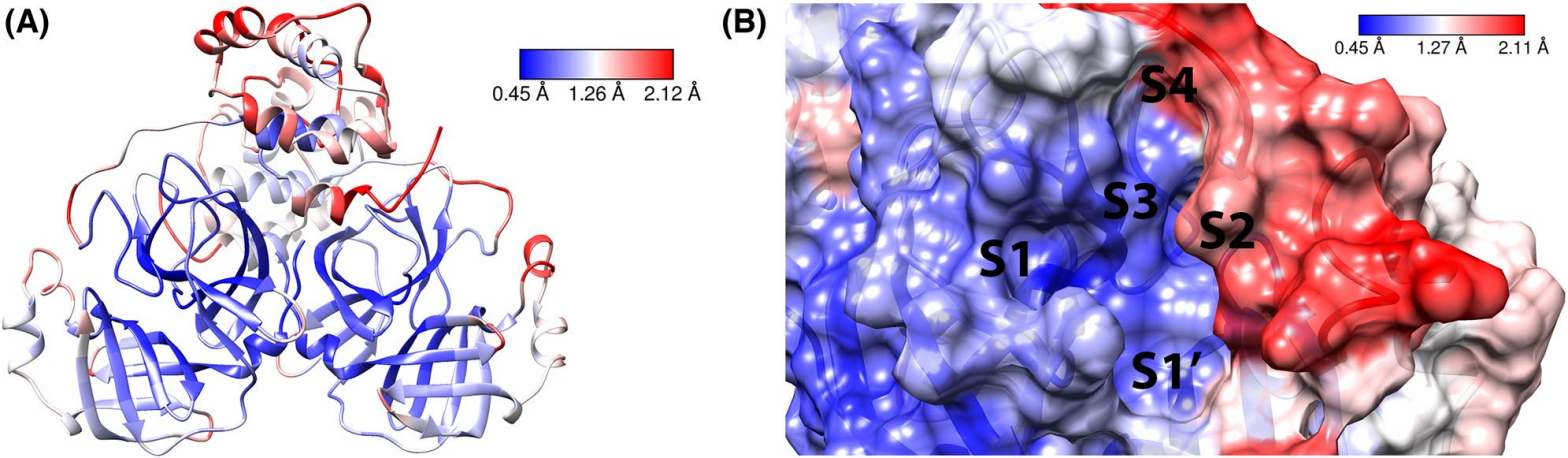
(**A**) RMSF values of MD simulation for 6LU7 complex applied on SARS-CoV-2 M^pro^ dimer ribbons representation. (**B**) RMSF values of MD simulation for 6LU7 complex applied on SARS-CoV-2 M^pro^ active site surface.

We performed 500 ns MD simulations for 15 protein–ligand complexes with experimental ligand pose available (Table S1) and 38 protein–ligand complexes (Table S2, S3) with predicted ligand pose from flexible molecular docking. The root-mean-square-deviation (RMSD) and the per-residue-root-mean-square-fluctuation (RMSF) over alpha carbon atoms for the MD production stage of all systems are reported in Supplementary Data 1. After the MD simulation extension of the top-ranked complexes from MM/PBSA calculations, the RMSD and RMSF graphs were obtained (Figure S8, S9, S10, S11). For some complexes, the RMSD graphs showed possible conformational changes due to abrupt fluctuations. To understand the protein flexibility in detail Fig. 3 presents the M^pro^ crystal structure of 6LU7 complex in ribbons and surface representation colored with RMSF values.

We identified three important zones with higher RMSF values. The first zone is located around the residues present in the 45–55 range, the second one corresponds to the residues located in the range 189–193, and the third zone corresponds to the range 200–300. Besides, large movements at the terminal residues were also detected. The first two zones correspond to S2 (45–55) and S4 (189–193) subsites, so the flexibility of these areas may be important in protein–ligand interaction. To highlight, subsite S2 presented a gradual loss of secondary structure from alpha-helix to loop for some complexes, as shown in Figure S24 for the 7JU7 complex. Furthermore, in some protein crystals such as 6Y2F, we observed missing residues in the S2 subsite, which might be related to the flexibility of this zone. Respecting covalent and non-covalent inhibitors, no remarkable differences between these categories were appreciated in the RMSF plots or visual inspections of the MD trajectories in consideration of the largest conformational changes occur in the S2/S4 subsites.

In order to perform an in-depth review of the MD trajectories, we carried out principal component analysis (PCA) for alpha carbons and H-bond analysis for some complexes. The PCA graphs for 6LU7, 6LZE, 6Y2F, and 7K6D complexes (Figure S12) showed that the SARS-CoV-2 M^pro^ suffered conformational changes, especially between 500 and 1000 ns. The eigenvectors corresponding to principal components were plotted using porcupine plots (Figure S13, S14, S15, S16) to illustrate in a visual way which are the protein movements that happened in the MD simulations. We found that these motions belong to the terminal ends, domain III, and subsites S2/S4 which agrees with the flexible zones found in the RMSF plots, being the motions of terminal ends and domain III the most prominent. The observed motions correlate to clockwise or counterclockwise rotations of domain III and subsites S2/S4 which agrees with the findings of similar MD studies^33^. Furthermore, the magnitude of these movements varies between the two protomers, suggesting asymmetric behavior in the structural units that constitute the dimer. In parallel, superpositions of the MD simulations frames and the X-ray structure were generated for certain protein–ligand complexes (Figure S22), which showed that domain III in dimer form is maintained at an approximately constant distance from domain II, which is in line with other studies^34^. On the other hand, H-bonds involved in protein–ligand interactions were studied for the 6LU7, 6Y2F, 7K6D, and 0026 complexes (Figure S23). The H-bonds with the highest frequency of occurrence belonged to Glu-166, Phe-140, His-163, His-164, Cys-145, and Gly-143 residues. Finally, although it is outside the scope of this article, interesting results based on H-bond analysis for N-finger residues between protomers are reported in the Supplementary Information section called: N-finger H-bond analysis.

### MM/PBSA affinity ranking of coronavirus inhibitors

We performed a sequential protocol of MM/PBSA protein–ligand binding energy calculations based on MD trajectories, from them an affinity ranking was created (Table S15) which contains 23 actives. These compounds are divided in 4 non-covalent and 19 covalent inhibitors. The extension of the 500 ns MD trajectories did not show appreciable raking differences, hence the average protein–ligand binding energy values remained approximately constant for the extra 500 ns. A set of 10 inhibitors lacking crystallographic pose but exhibiting excellent protein–ligand binding energy values (under − 25 kcal/mol) belong to the raking. A point to highlight is the structure diversity of the identified active compounds, considering that among these compounds we identified inhibitors with Michael acceptor, ketone, aldehyde warheads, and non-covalent inhibitors with some scaffolds based on natural products.

**Figure 4:**
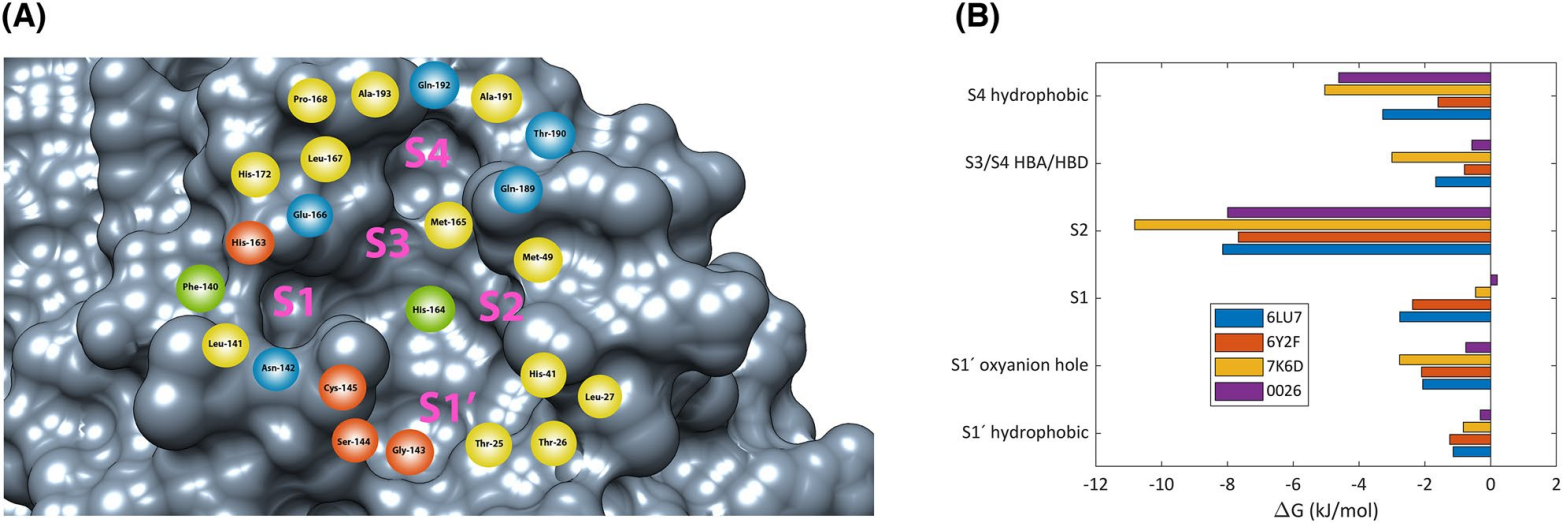
(**A**) Key residues for the protein–ligand interaction obtained through MD simulations and MM/PBSA calculations. HBA (red), HBD (green), HBA/HBD (blue), and hydrophobic (yellow). (**B**) Subsite contribution profile of protein–ligand binding energy for complexes (A) 6LU7, (B) 7K6D, (C) 6Y2F, and (D) 0026 normalized by the number of residues. S4 hydrophobic (Leu-167, Pro-168, Ala-191 and Ala-193), S3/S4 HBA/HBD (Glu-166, Gln-189, Thr-190 and Gln-192), S2 (Met-49 and Met-165), S1 (Phe-140, Leu-141, Asn-142, His-163, His-164 and His-172), S1’ oxyanion hole (Gly-143, Ser-144, Cys-145), S1’ hydrophobic (Thr-25, Thr-26, Leu-27 and His-41).

The PCA-clustering procedure allowed the extraction of 181 ligand poses from the 23 MD trajectories (Table S16), and ligand–protein interaction diagrams with interaction frequency percentages were generated for the 23 protein–ligand complexes (Figure S36, S36, S37, S38, S39, S40). A comparison of the diagrams allowed us to find the residues with the highest number of protein–ligand interactions and the nature of these interactions (Fig. 4A). Thus, we classified the SARS-CoV-2 M^pro^ active site residues in 6 zones: S1’ oxyanion hole, S1’ hydrophobic, S1, S2, S3/S4 HBA/HBD, and S4 hydrophobic. The S1’ oxyanion hole zone is composed of Cys-145, Ser-144, and Gly-143 where the ligand interacts with N–H backbone groups through hydrogen bonds (Figure S44). For the S1’ subsite, we denominated “S1’ hydrophobic” a zone composed of His-41, Thr-25, Thr-26, and Leu-27 residues which interact with ligands employing hydrophobic contacts (Figure S44). Near the S1' subsite is located S1, composed of His-172, Leu-141, Asn-142, Phe-140, His-163, and His-164. Most S1 residues interact with the ligand through hydrogen bonds, except for Leu-141 and His-172 that interact through hydrophobic contacts (Figure S42). The Asn-142 residue interacts as HBA or HBD with ligand through side chain while Phe-140 interacts using backbone. Residues His-163 and His-164 interact using side chain and backbone, respectively. The S2 subsite, constituted by Met-49 and Met-165, interacts with the ligand utilizing hydrophobic contacts (Figure S41). Additionally, we found that the His-41 residue is sometimes located close to Met-49, thus assisting in hydrophobic contacts for S2. For S3 and S4 subsites, we generated two classifications: the first is called S3/S4 HBA/HBD which is composed of Glu-166, Gln-189, Gln-192, and Thr-190. The second is called S4 hydrophobic and is constituted by Leu-167, Pro-168, Ala-191, and Ala-193. The residues Glu-166, Gln-189, Gln-192, and Thr-190 showed the ability to act as HBA and HBD. Glu-166 interacts mostly using its backbone, although it occasionally interacts using its side chain (Figure S41). Gln-189 interacts by its side chain, while Gln-192 and Thr-190 do by both, their side chain and backbone (Figure S43). Finally, residues Leu-167, Pro-168, Ala-191, and Ala-193 interact with the ligand through hydrophobic contacts, although occasionally hydrogen bonds with the backbone are present (Figure S43).

To recognize which residues are the most energetically relevant in the protein–ligand interaction, a protein–ligand binding energy decomposition was performed for some complexes with a special focus on the already mentioned zones (Figure S25). The contributions for residues belonging to the 6 zones (Table S18 and Figure S46) were added to obtain the energetic contributions of the zones (Figure S45). The energy comparison between zones can be unequal since the number of residues that integrate the zones is not the same. To compensate for it, the energy values were normalized by the number of residues, which resulted in Fig. 4B. The zone that contributes the most to the protein–ligand binding energy is S2 where Met-165 is the residue with the highest protein–ligand binding energy for all complexes. On the other hand, the second highest contributing zone is S4 hydrophobic, with Pro-168 being the residue with the lowest binding energy values (highest contributor). The remaining zones appear to have similar contributions except S1' hydrophobic which is the zone with the lowest contribution to the binding energy. Pointing to find the relationship between alpha carbons movement and protein–ligand binding contribution average, protein–ligand free energy binding contribution plots were generated for 6LU7, 7K6D, 6Y2F, and 0026 systems along with the RMSF values for alpha carbons represented in the format of a heat map (Figure S26). These plots reaffirmed that S2 and S4 subsites are the ones that presented the highest mobility and contribution to the free energy (normalized by the number of residues).

Once the most relevant residues and zones of the active site were identified, the stability of the complexes was evaluated through the use of binding energy and time plots. Initially, binding energy vs time plots were obtained for the 23 complexes (Figure S27, S28, S29, S30). These plots show that the binding energy remained oscillating around constant values for some systems, while for others changes were observed. Despite these energetic changes, the energies for these systems remained at negative values indicating that the complexes continued to exhibit good stability. These changes were due to variations of the ligand poses, which were confirmed after a visual review of the MD trajectories. On account of the fact that the contribution profiles indicate an average of the energetic contribution over time, then contribution vs time plots were generated for the residues of the 6 zones (Figure S31, S32, S33, S34). This was done in order to inspect the stability of each individual interaction. As a result, it can be appreciated that the majority of interactions remained stable, unlike some residues belonging to subsite S4/S1’, which showed a loss of interactions at certain times during the MD simulation.

### Pharmacophore modeling

To classify the 181 ligand poses in several protein–ligand conformations, the protein–ligand structures corresponding to each of the 181 ligand poses were extracted from MD trajectories and a principal component analysis was performed on the active site C-α atomic coordinates. Following, the three components with the highest eigenvalue were used for a clustering process with the k-means method (Figure S7), and 18 active-site-conformation cluster were finally obtained. The representative structures of each cluster are shown in Figure S47.

After elucidation, clustering, and merging, of 181 initial pharmacophore models (Figure S48), we obtained 18 representatives for non-covalent interaction modes (Figure S49). Features with a low occurrence frequency or low contribution to binding energy were configured as optional features. Moreover, one pharmacophoric feature (HBA) near the oxyanion hole was changed to the residue bonding point feature (Figure S50). This is supported by the finding of no remarkable differences between these covalent and non-covalent inhibitors in active site flexibility from MD trajectories. Models which have the HBA feature located on the oxyanion hole were named non-covalent models and models which have the residue bonding point feature located on the oxyanion hole were named covalent models. The residue bonding point feature detects electrophilic heads (warheads) such as aldehydes, ketones, nitriles, or Michael acceptors. The 18 covalent and non-covalent models were subjected to a validation process to measure the ability of the models to discriminate between active and decoy compounds using metrics such as ROC-AUC (Area Under ROC Curve) and BACC (Balanced Accuracy). The 23 actives from MM/PBSA calculations were classified into covalent and non-covalent inhibitors according to the presence or absence of reactive fragments such as Michael acceptors, aldehydes, or ketones in the P1' position of the inhibitor. Being that these reactive fragments are capable of accepting HB, then the 23 actives were classified into 23 non-covalent and 19 covalent ligands. The validation process was composed of two steps: (1) reduction of the rigidity of the models increasing spheroid tolerances to filter models with bad performance more easily, and (2) decreasing spheroid tolerance and optimizing weight values to generate tighter models. The results for the first validation are reported in Table S19 and Table S20. The five models that presented the best performance were subjected to the second validation as illustrated in Table S21 and Table S22.

Referring to the feature location for the pharmacophore models, we observed that features have a high similarity to the interaction zones found in Fig. 4. Features located in S2 are principally hydrophobic considering that inhibitors present aromatic rings or aliphatic groups which can position on the S2 subsite. For S1 subsite features, we found are in the majority HB donor/acceptors, as a consequence of the existence of lactam fragments with carbonyl and amine functions that can act as HB acceptor and donor respectively. In the case of S3/S4 subsites, we noticed the presence of hydrophobic and HB donors/acceptors features. Ultimately, for S1’ subsite, we encountered HBA and hydrophobic features located near to oxyanion hole and His-41 respectively. Comparing with the MM/PBSA protein–ligand binding contributions (Table S18, Fig. 3B, S45, S46) the most relevant features for binding energy with high frequency percentages (Figure S35, S36, S37, S38, S39, S40) are: the hydrophobic group in S2 (Met-49, Met-165), the hydrophobic group in S4 (Pro-168), HBA or residue bonding point in oxyanion hole (Cys-145), HBD associated to Phe-140 and HBD/HBA related with Glu-166. Comparing our pharmacophore models with other pharmacophore modeling studies^35^ we found the presence of some common features related to oxyanion hole residues, S2 residues, His-164, and Glu-166.

**Figure 5:**
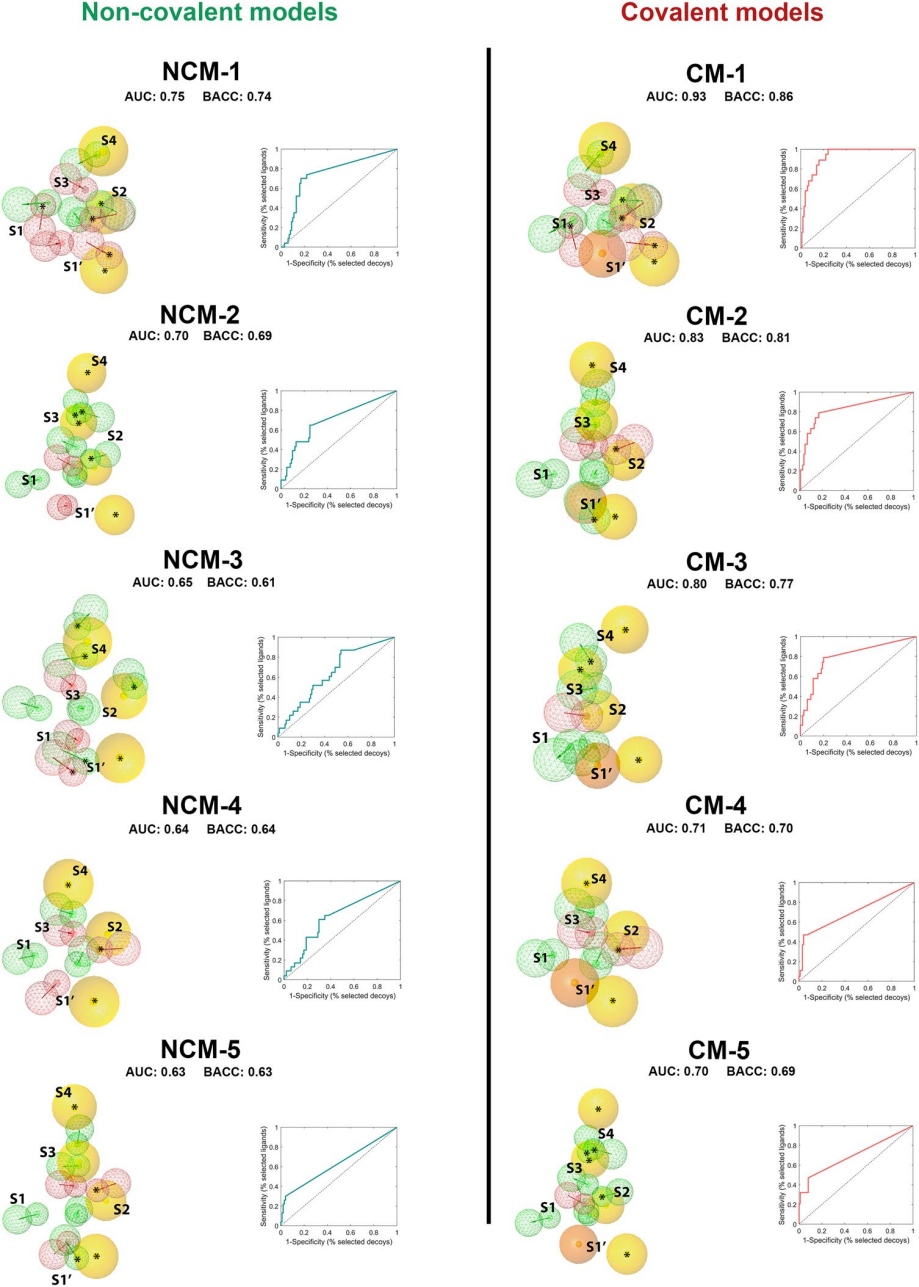
Protein–ligand-based pharmacophore models and ROC curves. The models are ordered from best to worst according to ROC-AUC and BACC values. Non-covalent models (NCM) on the left and covalent models (CM) on the right. HBD (green), HBA (red), hydrophobic (yellow), residue bonding point (orange), and optional feature (*). Each pharmacophore model corresponds to an active-site-conformation cluster, conventions can be found in Figure S49 and Figure S50.

**Figure 6:**
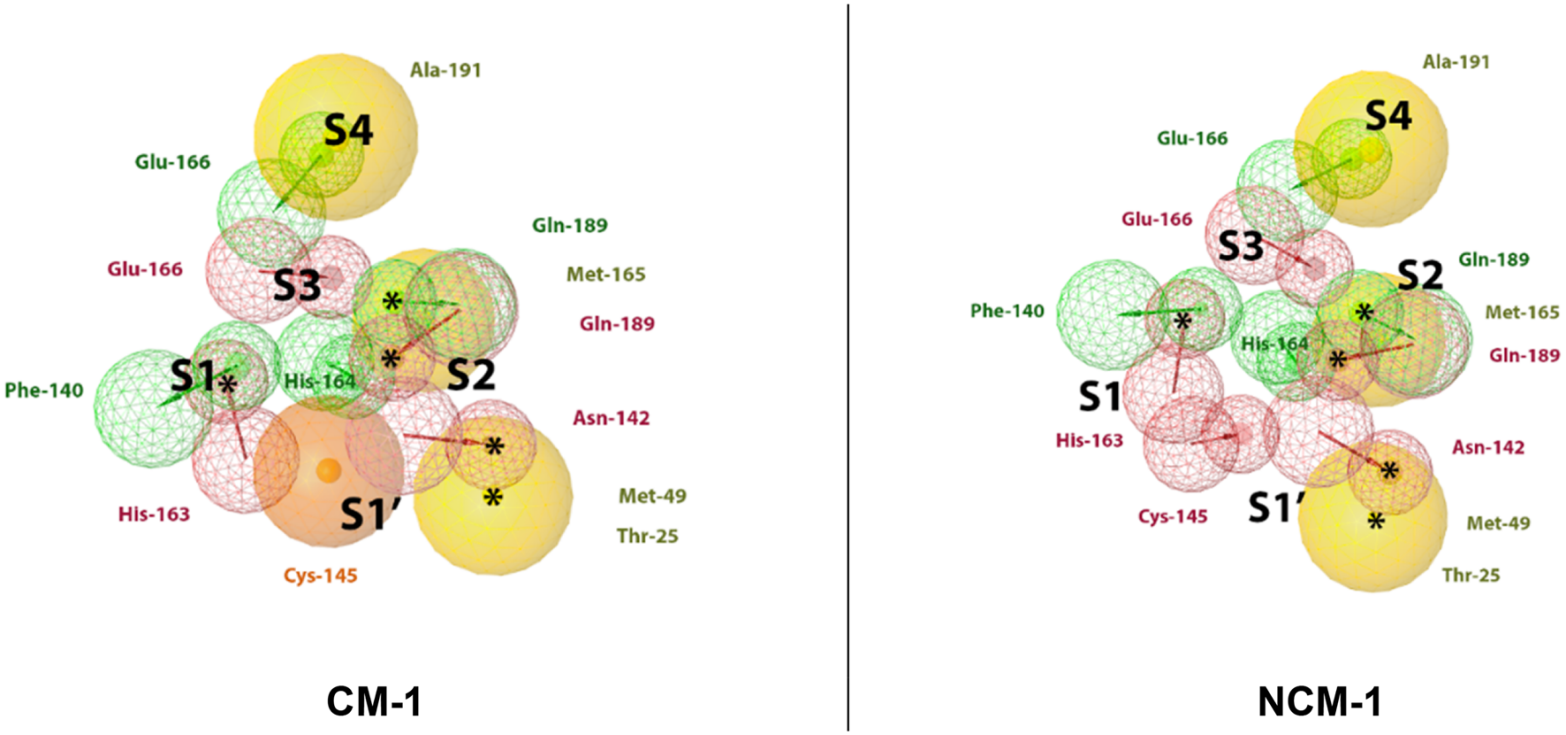
Best covalent CM-1 and non-covalent NCM-1 models according to ROC-AUC and BACC values. HBD (green), HBA (red), hydrophobic (yellow), residue bonding point (orange), and optional feature (*). The residues reported in the models were identified by LigandScout.

The best 5 covalent and non-covalent models are shown in Fig. 5, S51, and S52. We identified that the non-covalent pharmacophore model (NCM-1) with the best performance has a ROC-AUC value of 0.75 and a BACC value of 0.74. On the other hand, the best covalent pharmacophore model (CM-1) presented a ROC-AUC value of 0.93 and a BACC of 0.86. The CM-1 and NCM-1 share 10 identical pharmacophoric features: 4 HBA, 4 HBD, and 3 hydrophobic groups (Fig. 6). Among these 10 pharmacophoric features, 5 features are configured as optional, namely 3 HBA, 1 HBD, and 1 hydrophobic group. Furthermore, to test the influence of the features on the ROC-AUC values, two tests of the models in Fig. 5 were performed: in the first all features configured as optional were removed (Table S23, S24), and in the second the features belonging to the oxyanion hole were deleted (Table S25, S26). As a result of the first test, it was determined that the elimination of the optional features causes a total loss of the ability of the models to detect molecules, with ROC-AUC values between 0.5 and 0.0. On the other hand, for the second test, a decrease in ROC-AUC values was obtained, although not as drastic as in the first test. This means that the models retain some predictive capacity without the presence of the oxyanion hole feature.

**Figure 7:**
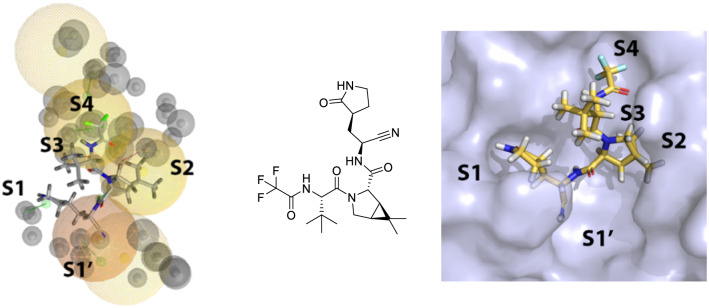
Ligand pose of Nirmatrelvir obtained through CM-2 on the left. Experimental ligand pose of Nirmatrelvir on the right. (PDB 7SI9) The nitrile group in the experimental pose does not appear correctly as it is covalently bonded to the protease. HBD (green), HBA (red), hydrophobic (yellow), residue bonding point (orange), and exclusion volume (gray).

Concerning pharmacophore screening against Nirmatrelvir, the results can be found in Tables S27 and S28. The covalent and non-covalent models displayed good performance with pharmacophore score functions above 70 in all cases, detecting Nirmatrelvir as a true positive. Additionally, the CM-2 model was able to replicate the experimental pose of the Nirmatrelvir (Fig. 7). The nitrile group was located at S1', the lactam at S1, the hydrophobic bicyclic fragment at S2, the peptide backbone at S3/S2, and the trifluoromethyl at S4. The interactions related to the pharmacophore features correspond to those observed in the ligand set used, which are: S1 (Phe-140 and His-164), S1′ (Gly-143), S2 (Met-49 and Met-165), and S3 (Glu-166). Monitoring the electrophilic fragments corresponding to the covalent inhibitors of our set of ligands, in conjunction with the Nirmatrelvir molecule, we found differentiation between the aldehyde, nitrile, ketone, and Michael acceptor categories. Inhibitors based on aldehydes and nitriles did not satisfy the hydrophobic feature of S1' due to the absence of hydrophobic groups at the P1' position of the inhibitor, which is not the case for ketones and Michael acceptors.

## Discussion

A promising COVID-19 treatment strategy is the generation of SARS-CoV-2 M^pro^ inhibitors. Knowledge and understanding of the most relevant protein–ligand interactions for the inhibitory capacity are crucial for developing molecules with optimal selectivity and efficacy. Based on this motivation, we performed an in silico protocol of docking, MD simulations, clustering, and MM/PBSA free energy calculations to model, identify, and discriminate the essential physicochemical features favoring the interaction between SARS-CoV-2 M^pro^ and inhibitors within two categories: covalent and non-covalent. After MD simulations and MM/PBSA calculations, we observed that the most influential actives site zones primarily corresponded to subsites S2 and S4, which were not only accounted for higher binding energy with ligands but were also more flexible compared to subsite S1'. Because this latter subsite has been long known to play key role in covalent binding, this finding may shift the current paradigm for non-covalent M^pro^ inhibitors to have a more prominent role. Finally, generated pharmacophore models showed excellent performance, as evidenced by the positive control of the Nirmatrelvir molecule. The search for pharmacophoric features has previously been carried out in high quality different studies^15^, but in the present research, the pharmacophore modeling was performed using long-extended trajectories, which allows considering the flexibility of the M^pro^ active site in a broader manner represented by exclusion volume locations.

The employment of known active compounds lacking crystallographic pose, as well as the binding site analysis of the M^pro^, were pivotal to shed insight into the protease's intricate features. The advantage of using compounds with experimentally validated potency played a key role in capturing different patterns around protein–ligand interactions that would be eventually used as input during the elucidation of pharmacophore models. In line with this observation, the more structure diversity the ligand population has, the more pharmacophoric features crucial to ensure activity will be potentially captured. Complementary to these results, the binding site analysis we performed for SARS-CoV-2 M^pro^ proved to be helpful to rationalize the structural comparison between protein–ligand complexes and apoproteins crystals. To highlight, the larger volume of the active site for complexed crystals compared to the free state (apo) crystals may be associated with the presence of hydrophobic chemical moieties in the ligand able to favor its occupation of S2 subsite, as these latter have been previously reported as playing a role as gatekeepers and hence been able to modify the active site size^13^. Moreover, the volume data from protein–ligand complex crystals suggest that SARS-CoV-2 M^pro^ protein–ligand complexes exhibit large conformational changes within the active site region. This high flexibility agrees with other investigations showing that SARS-CoV-2 M^pro^ active site region undergoes important conformational variations in the same region^36,37^. Based on these analyses, we selected a set of residues for flexible molecular docking: this approach demonstrated to be advantageous to obtain a first approximation of the pose and binding energy, which was later refined using a combination of MD simulations and MM/PBSA calculations focused on the best-scored protein–ligand complexes.

One of the most important observations from this study is that MD simulations, PCA calculations and k-means procedure proved effective in successfully retrieving the conformational diversity of SARS-CoV-2 M^pro^. In good agreement with other studies^38^, such diversity demonstrated to be particularly pronounced in domain III and S2/S4 subsites motions, suggesting that the PCA and the k-means procedure were effective in extracting dramatic patterns in active site motion^29^. Likewise, the most flexible zones were not detected in S1′, indicating that the active site is likely able to adopt conformations, at least in the pre-reaction complex, irrespective of whether a covalent or non-covalent inhibitor is present. Interestingly, since S2 and S4 tended to show a large affinity for hydrophobic groups and to adopt secondary loop structures in the MD simulations, we propose that the observed flexibility for M^pro^ active site may be due to the subsite specificity. Because the hydrophobic interaction does not require directionality, it can be speculated that the flexibility is derived owing to it is not required that S2 and S4 are located in a particular fashion to facilitate the interaction^22^. Concerning the limitation of having no replicas for our long-range MD simulations, it is worth highlighting that we did not intend to determine the exact behavior of a specific complex, but rather to gather information from independent MD simulations of several complexes that provided a high conformational diversity. Nevertheless, the latter was not reflected in dramatic changes in the protein–ligand binding free energy during the simulation time. Therefore, considering the relevant population of protein–ligand complexes used during this study, we believe that the combined protocol of microsecond-long MD simulations and MM/PBSA was informative enough to capture different patterns around protein–ligand interactions and active site flexibility that would be eventually used as input during the elucidation of pharmacophore models. These findings not only contributed to confirming results obtained by previous studies^35^ but also to finding critical and exploitable features of non-covalent and covalent pharmacophore models (*vide infra* for details).

A noteworthy aspect to mention is the differences and similarities between model categories under study and the particular chemical moieties identified by each one of them. Even if the covalent models exhibited a better performance compared to the non-covalent models according to our ROC curve analysis, it is worth noting the absence of orientation from the residue bonding point feature compared to the HBA feature, which allows the entire area of the oxyanion hole to be covered without the need to use vectors. It could, in turn, suggest that the non-directional interaction of the feature located in the oxyanion hole would remarkably enhance the covalent model to detect more active ligands. However, we are not unaware that the more limited data of non-covalent inhibitors compared to covalent ones may influence the pharmacophore model performance within this particular category. Nevertheless, in terms of the chemical moieties, a detailed comparison between the pharmacophore features of our models and previously reported structure–activity relationship (SAR) studies, revealed various similarities among fragments associated with better potency (and some other physicochemical features) by using a similar ligand set^39^, including primarily peptide scaffolds, cyclohexyl and cyclohexyl methyl groups. Peptide scaffolds contain HBA and HBD groups capable of interacting with Glu-166 and His-164 residues while cyclohexyl and cyclohexyl methyl groups correspond to hydrophobic groups able to interact with Met-165, His-41, and Met-49. MD simulations of several complexes of M^pro^ with diverse ligands allowed us to identify this set of essential physicochemical features favoring the interaction between SARS-CoV-2 M^pro^ and inhibitors, complementing the previous results and helping to accomplish the overall purpose of the study. Nevertheless, we consider that for inhibitors design, the presence, absence, and/or optionality of certain physicochemical features depend on the active site regions to be occupied by the ligand. On the one hand, we noticed that active site zones that contribute most to the binding free energy were not located in S1' for most of the compounds under study, particularly the non-covalent ones: a prime example is the well-known inhibitor 7JU7, which managed to rank second in the affinity ranking occupying S1 and S2 subsites, but no S1'. Considering the covalent inhibitor poses of active molecules, the majority occupied the S2 and S4 subsites, which represents a key finding for the design of non-covalent inhibitors because strong classical interactions with the active site might not strictly depend on fragments directly interacting with the oxyanion hole. On the other hand, the use of covalent pharmacophore models to discover SARS-CoV-2 M^pro^ inhibitors appears to be promising, as evidenced by the results obtained for the Nirmatrelvir molecule. Covalent inhibitors allow to obtain longer residence times on the pharmacological targets and therefore permit using lower doses^40^. From the above evidence, we could infer that each inhibitor class has a wide variety of interesting attributes that should encourage the scientific community to continue working on the generation of both covalent and non-covalent inhibitors.

In summary, we demonstrated that a cascade in silico protocol of docking, MD simulations, and MM/PBSA calculations proved useful to elucidate pharmacophore models for covalent and non-covalent inhibitors of SARS-CoV-2 M^pro^. The participation of MM/PBSA calculations was crucial as it allowed finding binding predicted free energy values that consider flexibility and solvent effects^28^ compared to conventional docking scoring functions. On the other hand, cavities calculations combined with MD simulations revealed the high flexibility of the SARS-CoV-2 M^pro^ active site, which is in line with prior reports^36–38^. For pharmacophore modeling, this discovery inspired us to perform a clustering campaign to extract active site conformations from MD trajectories. In this manner, pharmacophore models consider M^pro^ flexibility, apparently a crucial factor in the search for inhibitors for this receptor. Furthermore, understanding the difference in SARS-CoV-2 M^pro^ behavior in the presence of covalent and non-covalent inhibitors may play a critical element in future studies. A further benefit of the usage of pharmacophore models is the possibility to discover new scaffolds^41^ that may act similarly to peptidomimetics and exhibit high affinities towards SARS-CoV-2 M^pro^ (scaffold hopping), as has been performed for 3C enterovirus proteases^42^. In recent months, interesting results have been obtained in investigations focused on the search for natural products that can act as SARS-CoV-2 M^pro^ inhibitors^43–45^. In this regard, we believe that the use of the pharmacophore models opens the door to inhibitors design based on natural products, which would benefit drug discovery in distinct modes^46^. We expect that our pharmacophore models will be used in the design of new molecules that can exhibit high levels of affinity towards SARS-CoV-2 M^pro^ and may function as a treatment for COVID-19.

## Supplementary Information

Supplementary Information.

## Data Availability

Supplementary Data can be found here: https://doi.org/10.6084/m9.figshare.19729837. Additional raw data will be available upon request.
