# Supplementary material for: Novel covalent and non-covalent complex-based pharmacophore models of SARS-CoV-2 main protease (Mpro) elucidated by microsecond MD simulations
Source: Sci Rep. 2022 Aug 18;12:14030. doi: 10.1038/s41598-022-17204-0 (PMC9386674; doi:10.1038/s41598-022-17204-0)
Supplement: Supplementary file 1 — Supplementary Information. [file 41598_2022_17204_MOESM1_ESM.docx]

**Supplementary information**

**Novel covalent and non-covalent complex-based pharmacophore models of SARS-CoV-2 main protease (Mpro) elucidated by microsecond MD simulations**

Yasser Hayek Orduz^1^, Andrés Felipe Vásquez^1,3^, María Francisca Villegas Torres^2,4^, Paola Andrea Caicedo Burbano^4^, Luke E. K. Achenie^5^, Andrés Fernando González Barrios^1*^

^1^Grupo de Diseño de Productos y Procesos (GDPP), Department of Chemical and Food Engineering, Universidad de los Andes, Bogotá, Colombia.

^2^Centro de Investigaciones Microbiológicas (CIMIC), Department of Biological Sciences, Universidad de los Andes, Bogotá, Colombia.

^3^Naturalius SAS, Bogotá, Colombia.

^4^Grupo Natura, Faculty of Sciences, Universidad ICESI, Cali, Colombia.

^5^Department of Chemical Engineering, Polytechnic Institute and State University from Virginia (Virginia Tech), USA.

***Correspondence:**

Corresponding Author

andgonza@uniandes.edu.co

**Table of contents**

| Figure S1. Examples of non-covalent inhibitors and covalent inhibitors for coronavirus main protease. | **1** |
| --- | --- |
| Table S1. Inhibitors of the SARS-CoV-2 main protease with protein crystal reported in the Protein Data Bank. | **2** |
| Table S2. Inhibitors of the SARS-CoV-2 main protease without protein crystal reported in the Protein Data Bank. | **3** |
| Table S3. Inhibitors of the SARS-CoV main protease. | **4** |
| SARS-CoV-2 M^pro^ active site flexibility study. | **5** |
| Table S4. Volume and area of apo SARS-CoV-2 M^pro^ active site cavity for several crystals. | **5** |
| Table S5. Volume and area of protein-ligand complex SARS-CoV-2 Mpro active site cavity for several crystals. | **5** |
| Table S6. Volume and area of apo SARS-CoV-2 M^pro^ 6YB7 crystal for 5 CASTp cavities of 35 identified. | **5** |
| Figure S2. Active site cavity for SARS-CoV-2 M^pro^ 6YB7 crystal identified with CASTp. | **5** |
| Figure S3. Met-49 movement and surroundings between the apoprotein 6YB7 (green) and complex 6LU7 (purple). | **6** |
| Figure S4. Active site surface of 6YB7 apoprotein (green) and 6LU7 complex (purple) emphasizing Met-49 residue. | **6** |
| Flexible molecular docking. | **7** |
| Table S7. Flexibility rules applied to 6 residues of SARS-CoV-2 main protease. | **7** |
| Figure S5. 0019 thermodynamic pose and 0019 kinetic pose. | **7** |
| Molecular dynamics stability | **8** |
| Figure S6. Steps to generate an affinity ranking. | **8** |
| Figure S7. Steps for clustering and PCA calculations. | **9** |
| Figure S8. Superposition of RMSD C-α values of MD trajectories for protein-ligand complexes of active ligands identified with MM/PBSA calculations. | **10** |
| Figure S9. Superposition of RMSF C-α values of MD trajectories for protein-ligand complexes of active ligands identified with MM/PBSA calculations. | **10** |
| Figure S10. RMSD C-α values of MD trajectories for protein-ligand complexes of active ligands identified with MM/PBSA calculations. | **11** |
| Figure S11. RMSF C-α values of MD trajectories for protein-ligand complexes of active ligands identified with MM/PBSA calculations. Protomer A (Residues 1-306), protomer B (Residues 307-612). | **12** |
| Figure S12. Principal components analysis graph for C-α of MD trajectories for 6LU7, 6LZE, 6Y2F and 7K6D complexes. | **13** |
| Figure S13. Porcupine plot of protein movements corresponding to PC1 and PC2 for C-α of MD trajectories for 6LU7 complex. | **14** |
| Figure S14. Porcupine plot of protein movements corresponding to PC1 and PC2 for C-α of MD trajectories for 6LZE complex. | **15** |
| Figure S15. Porcupine plot of protein movements corresponding to PC1 and PC2 for C-α of MD trajectories for 6Y2F complex. | **16** |
| Figure S16. Porcupine plot of protein movements corresponding to PC1 and PC2 for C-α of MD trajectories for 7K6D complex. | **17** |
| Figure S17. N-finger interactions of Ser-1 and Arg-4 residues for 1UK3 protein crystal. Hydrogen bonds in yellow. | **18** |
| Figure S18. Occupancy percentages of N-finger hydrogen bonds donors (D) and acceptors (A) for 6LU7 MD simulation. | **18** |
| Figure S19. Occupancy percentages of N-finger hydrogen bonds donors (D) and acceptors (A) for 6Y2F MD simulation. | **19** |
| Figure S20. Occupancy percentages of N-finger hydrogen bonds donors (D) and acceptors (A) for 7K6D MD simulation. | **19** |
| Figure S21. Occupancy percentages of N-finger hydrogen bonds donors (D) and acceptors (A) for 0026 MD simulation. | **20** |
| Figure S22. Comparison of 6LU7, 6Y2F and 7K6D crystal structures with several MD frames for one protomer. | **21** |
| Figure S23. Occupancy percentages of protein-ligand hydrogen bonds donors (D) and acceptors (A) for complexes (A) 6LU7, (B) 7K6D, (C) 6Y2F and (D) 0026. | **22** |
| Figure S24. Movement of residues 45-55 corresponding to simulation times of 100, 500 and 1000 ns for the 7JU7 complex. | **23** |
| Table S8. ΔG MM/GBSA and XP Gscore of the best poses of flexible molecular docking inhibitors from Table S2. | **24** |
| Table S9. ΔG MM/GBSA and XP Gscore of the best poses of flexible molecular docking inhibitors from Table S3. | **24** |
| Table S10. ΔG MM/PBSA for inhibitors from Table S1 with 500 ns MD production. | **24** |
| Table S11. ΔG MM/PBSA for inhibitors from Table S2 with 500 ns MD production. | **24** |
| Table S12. ΔG MM/PBSA for inhibitors from Table S3 with 500 ns MD production. | **25** |
| Table S13. First ΔG MM/PBSA ranking (affinity ranking) for inhibitors. | **25** |
| Table S14. ΔG MM/PBSA for inhibitors from Table S11 with 1000 ns MD production excluding ligands with high movement on the active site. | **26** |
| Table S15. Second ΔG MM/PBSA ranking for inhibitors. | **26** |
| Figure S25. Residue contribution profile of protein-ligand binding energy for complexes (A) 6LU7, (B) 7K6D, (C) 6Y2F and (D) 0026. | **27** |
| Figure S26. Residue contribution profile of protein-ligand binding energy and RMSF C-α values for complexes (A) 6LU7, (B) 7K6D, (C) 6Y2F and (D) 0026. | **28** |
| Figure S27. MM/PBSA ligand-protein binding energy vs MD simulation time for 0003, 0004, 0006, 6LU7, 6LZE and 6W63 complexes. | **29** |
| Figure S28. MM/PBSA ligand-protein binding energy vs MD simulation time for 6WTK, 6XMK, 6XR3, 6Y2F, 0007 and 7C8R complexes. | **30** |
| Figure S29. MM/PBSA ligand-protein binding energy vs MD simulation time for 7C8T, 7JU7, 7JYC, 7K6D, 7K40 and 0012 complexes. | **31** |
| Figure S30. MM/PBSA ligand-protein binding energy vs MD simulation time for 0013, 0019, 0026, 0030 and 0035 complexes. | **32** |
| Figure S31. MM/PBSA ligand-protein binding energy contributions of several active site residues vs MD simulation time for 6LU7 system. | **33** |
| Figure S32. MM/PBSA ligand-protein binding energy contributions of several active site residues vs MD simulation time for 6Y2F system. | **34** |
| Figure S33. MM/PBSA ligand-protein binding energy contributions of several active site residues vs MD simulation time for 7K6D system. | **35** |
| Figure S34. MM/PBSA ligand-protein binding energy contributions of several active site residues vs MD simulation time for 0026 system. | **36** |
| Table S16. Number of ligand-pose-clusters obtained for each protein-ligand complex. | **37** |
| Table S17. Number of protein-ligand structures contained in each active-site-conformation-cluster (ASCC). | **37** |
| Figure S35. Ligand-protein interaction diagrams with interaction frequency percentages of representative structures (Figure S5) for 0003, 0004, 0006 and 6LU7 complexes. | **38** |
| Figure S36. Ligand-protein interaction diagrams with interaction frequency percentages of representative structures (Figure S5) for 6LZE, 6W63, 6WTK and 6XMK complexes. | **39** |
| Figure S37. Ligand-protein interaction diagrams with interaction frequency percentages of representative structures (Figure S5) for 6XR3, 6Y2F, 0007 and 7C8R complexes. | **40** |
| Figure S38. Ligand-protein interaction diagrams with interaction frequency percentages of representative structures (Figure S5) for 7C8T, 7JU7, 7JYC and 7K6D complexes. | **41** |
| Figure S39. Ligand-protein interaction diagrams with interaction frequency percentages of representative structures (Figure S5) for 7K40, 0013, 0019 and 0026 complexes. | **42** |
| Figure S40. Ligand-protein interaction diagrams with interaction frequency percentages of representative structures (Figure S5) for 0012, 0035 and 0030 complexes. | **43** |
| Figure S41. Ligand poses from MD simulations corresponding to interactions of crucial residues for S2/S3 subsite. | **44** |
| Figure S42. Ligand poses from MD simulations corresponding to interactions of crucial residues for S1 subsite. | **44** |
| Figure S43. Ligand poses from MD simulations corresponding to interactions of crucial residues for S4 subsite. | **45** |
| Figure S44. Ligand poses from MD simulations corresponding to interactions of crucial residues for S1’ subsite. | **46** |
| Figure S45. Subsite contribution profile of protein-ligand binding energy for complexes (A) 6LU7, (B) 7K6D, (C) 6Y2F and (D) 0026. | **46** |
| Table S18. Protein-ligand binding energy contribution values (kJ/mol) of important active site residues for complexes 6LU7, 7K6D, 6Y2F and 0026. | **47** |
| Figure S46. Protein-ligand binding energy contribution graphs (kJ/mol) of important active site residues for complexes 6LU7, 7K6D, 6Y2F and 0026. | **48** |
| Figure S47. Representative structure (centroid) for each active-site-conformation-cluster (18). | **49** |
| Figure S48. Overlapped pharmacophore models (181) for each active-site-conformation-cluster (18). | **50** |
| Figure S49. Non-covalent pharmacophore models (NCM). | **51** |
| Figure S50. Covalent pharmacophore models (CM). | **52** |
| Figure S51. Best non-covalent pharmacophore models (NCM) with residues directly involved in the pharmacophoric features detected by LigandScout. | **53** |
| Figure S52. Best covalent pharmacophore models (CM) with residues directly involved in the pharmacophoric features detected by LigandScout. | **54** |
| Table S19. First validation for Non-covalent pharmacophore models. | **55** |
| Table S20. First validation for Covalent pharmacophore models. | **56** |
| Table S21. Second validation for Non-covalent pharmacophore models. | **57** |
| Table S22. Second validation for Covalent pharmacophore models. | **57** |
| Table S23. Performance of Non-covalent pharmacophore models with disabled optional features. | **57** |
| Table S24. Performance of covalent pharmacophore models with disabled optional features. | **57** |
| Table S25. Performance of Non-covalent pharmacophore models with disabled oxyanion feature. | **57** |
| Table S26. Performance of covalent pharmacophore models with disabled oxyanion feature. | **58** |
| Table S27. Virtual screening results for Nirmatrelvir using Covalent pharmacophore models. | **58** |
| Table S28. Virtual screening results for Nirmatrelvir using Non-covalent pharmacophore models. | **58** |

| **Non-covalent inhibitors** | **Covalent inhibitors** |
| --- | --- |
|  |  |

**Figure S1.** **Examples of non-covalent inhibitors (2GZ7 and Iguesterin) and covalent inhibitors (6LZE, 6LU7 and 6Y2F) for coronavirus main protease.** Electrophilic groups (warhead) are colored in red.

**Table S1. Inhibitors of the SARS-CoV-2 main protease with protein crystal reported in the**

**Protein Data Bank.**

| **6LU7** | **6LZE** | **6M0K** | **6M2N** |
| --- | --- | --- | --- |
| **** | **** | **** | **** |
| **EC_50_=16.77 µM**^1^ | **IC_50_=0.053 µM**^2^ | **IC_50_=0.04 µM**^2^ | **IC_50_=0.94 µM (SARS‑CoV)**^3^ |
| **6W63** | **6WTT** | **6XMK** | **6XR3** |
| **** | **** | **** | **** |
| **‑** | **IC_50_=0.03 µM**^4^ | **IC_50_=0.48 µM**^5^ | **KI=0.0063 µM (SARS‑CoV)**^6^ |
| **6Y2F** | **7C8R** | **7C8T** | **7JU7** |
| **** | **** | **** | **** |
| **IC_50_=0.67 µM**^7^ | **KI=0.058 µM(SARS‑CoV)**^8^ | **KI=0.053 µM(SARS‑CoV)**^8^ | **IC_50_=2.5 µM**^9^ |
| **7JYC** | **7K40** | **7K6D** | |
| **** | **** | **** | |
| **IC_50_=5.73 µM**^4^ | **IC_50_=4.13 µM**^4^ | **IC_50_=10.7 µM**^10^ | |

**Table S2. Inhibitors of the SARS-CoV-2 main protease without protein crystal reported in the**

**Protein Data Bank.**

| **0001** | **0002** | **0003** | **0004** |
| --- | --- | --- | --- |
| **** | **** | **** | **** |
| **IC_50_=13.74 µM**^4^ | **IC_50_=0.97 µM**^4^ | **IC_50_=10.38 µM**^4^ | **IC_50_=0.45 µM**^4^ |
| **0005** | **0006** | **0007** | **0008** |
| **** | **** | **** | **** |
| **IC_50_=3.9 µM**^4^ | **IC_50_=2.39 µM**^7^ | **IC_50_=0.18 µM**^7^ | **IC_50_=15.75 µM**^1^ |
| **0009** | **0010** | **0011** | **0012** |
| **** | **** | **** | **** |
| **IC_50_=0.53 µM**^11^ | **EC_50_=1.13 µM**^12^ | **EC_50_=5.73 µM**^12^ | **EC_50_=8.83 µM**^12^ |
| **0013** | **0014** | **0015** | **0016** |
| **** | **** | **** | **** |
| **EC_50_=9.36 µM**^12^ | **EC_50_=13.34 µM**^12^ | **IC_50_=33.17 µM**^13^ | **IC_50_=37.78 µM**^13^ |

**Table S3. Inhibitors of the SARS-CoV main protease.**

| **0017** | **0018** | **0019** | **0020** |
| --- | --- | --- | --- |
| **** | **** | **** | **** |
| **KI=0.003 µM**^6^ | **IC_50_=0.051 µM**^14^ | **IC_50_=0.065 µM**^15^ | **KI=0.073 µM**^16^ |
| **0021** | **0022** | **0023** | **0024** |
| **** | **** | **** | **** |
| **IC_50_=0.098 µM[**83] | **IC_50_=0.3 µM**^17^ | **KI=0.66 µM**^8^ | **KI=0.8 µM**^18^ |
| **0025** | **0026** | **0027** | **0028** |
| **** | **** | **** | **** |
| **IC_50_=0.98 µM**^19^ | **KI=2.20 µM**^20^ | **IC_50_=3 µM**^17^ | **IC_50_=5.5 µM**^21^ |
| **0029** | **0030** | **0031** | **0032** |
| **** | **** | **** | **** |
| **EC_50_=6.2 µM**^22^ | **KI=6.7 µM**^23^ | **IC_50_=6.8 µM**^21^ | **KI=8.27 µM**^24^ |
| **0033** | **0034** | **0035** | **0036** |
| **** | **** | **** | **** |
| **KI=9.1 µM**^25^ | **IC_50_=10 µM**^26^ | **KI=10.7 µM**^23^ | **IC_50_=70 µM**^27^ |
| **0037** | | **0038** | |
| **** | |  | |
| **IC_50_=45 µM**^27^ | | **KI=100 µM**^25^ | |

***SARS-CoV-2 M^pro^ active site flexibility study.*** We located cavities in the main protease that could act as protein-ligand binding sites. For this purpose, the protease in apo form (PDB code: 6YB7) and the CASTp server were used^28^, which identified 35 cavities (**Table S6**). The cavity with the highest volume based on molecular surface area exhibited a volume of 979.8 Å^3^ (**Figure S2**). Furthermore, comparing the cavity with protein-ligand complex structures (PDB code: 6LU7) the largest cavity was also the protein-ligand binding site. Additionally, a druggability analysis of the cavity was performed using SiteMap of Schrödinger 2020. The druggability score obtained was 1.067, which means that the SARS-CoV-2 M^pro^ active site could be a receptor for drug-like molecules^29^.

| **Table S4. Volume and area of apo SARS-CoV-2 M^pro^ active site cavity for several crystals.** | **Table S5. Volume and area of protein-ligand complex SARS-CoV-2 M^pro^ active site cavity for several crystals.** |
| --- | --- |
| \| **PDB code** \| **Resolution (Å)** \| **Volume (Å^3^)** \| **Area (Å^2^)** \| \| --- \| --- \| --- \| --- \| \| **6M03** \| 2.00 \| 1150.80 \| 846.40 \| \| **6M2Q** \| 1.70 \| 1273.20 \| 870.99 \| \| **6WQF** \| 2.30 \| 1117.10 \| 764.76 \| \| **6WTM** \| 1.85 \| 1181.90 \| 794.49 \| \| **6Y2E** \| 1.75 \| 1130.30 \| 771.35 \| \| **6Y84** \| 1.39 \| 1149.90 \| 772.45 \| \| **6YB7** \| 1.25 \| 1164.50 \| 782.03 \| \| **7K3T** \| 1.20 \| 986.35 \| 700.87 \| \| **Average** \| \| 1144.26 \| 787.92 \| \| **Standard deviation** \| \| 79.64 \| 52.14 \| \| **Variation coefficient** \| \| 0.070 \| 0.066 \| | \| **PBD code** \| **Resolution (Å)** \| **Volume (Å^3^)** \| **Area (Å^2^)** \| \| --- \| --- \| --- \| --- \| \| **6LU7** \| 2.16 \| 1419.00 \| 926.74 \| \| **6LZE** \| 1.50 \| 1722.30 \| 1093.20 \| \| **6M0K** \| 1.50 \| 1801.40 \| 1125.80 \| \| **6WNP** \| 1.45 \| 2322.00 \| 1569.40 \| \| **6WTJ** \| 1.90 \| 1350.20 \| 907.36 \| \| **6WTK** \| 2.00 \| 1201.00 \| 844.29 \| \| **6WTT** \| 2.15 \| 1645.90 \| 1090.90 \| \| **7BUY** \| 1.60 \| 1673.40 \| 1129.90 \| \| **Average** \| \| 1641.90 \| 1085.95 \| \| **Standard deviation** \| \| 343.22 \| 224.55 \| \| **Variation coefficient** \| \| 0.209 \| 0.207 \| |

The flexibility level of the M^pro^ protein-ligand binding site was studied calculating the cavity volume of different crystallized SARS-CoV-2 M^pro^ structures employing the SURFNET algorithm^30^. As a result, the average values and standard deviation of volume and area for the SARS-CoV-2 M^pro^ active site were obtained for 8 apoprotein structures (**Table S4**) and 8 protein-ligand complexes (**Table S5**). The existence of flexibility is highly relevant since in the case of high flexibility the protein-ligand interactions may be dependent on active site conformational changes. If the active site of the protease is flexible then different values ​​of volume and area of ​​the cavity will be obtained for different protein crystals^31^. This disparity can be observed in statistical measures such as standard deviation or variation coefficient. Specifically, for a flexible active site, it was expected to obtain a high standard deviation and high variation coefficient. The main difference between the active site surfaces for apoproteins and complexes was the shape of the S2 subsite (**Figure S4**). For apoproteins, the S2 subsite showed a smaller size in comparison to for complexes (**Figure S3**), which is due to the movement of the Met-49 backbone and side chain.

| **Table S6. Volume and area of apo** **SARS-CoV-2 M^pro^ 6YB7 crystal for 5 CASTp cavities of 35 identified.** The cavity with the largest volume and area is the active site.   \| **Cavity number** \| **Volume (Å^3^)** \| **Area (Å^2^)** \| \| --- \| --- \| --- \| \| 1 \| 979.8 \| 538.5 \| \| 2 \| 394.5 \| 237.8 \| \| 3 \| 270.2 \| 241.3 \| \| 4 \| 146.3 \| 121.6 \| \| 5 \| 96.8 \| 80.3 \| | 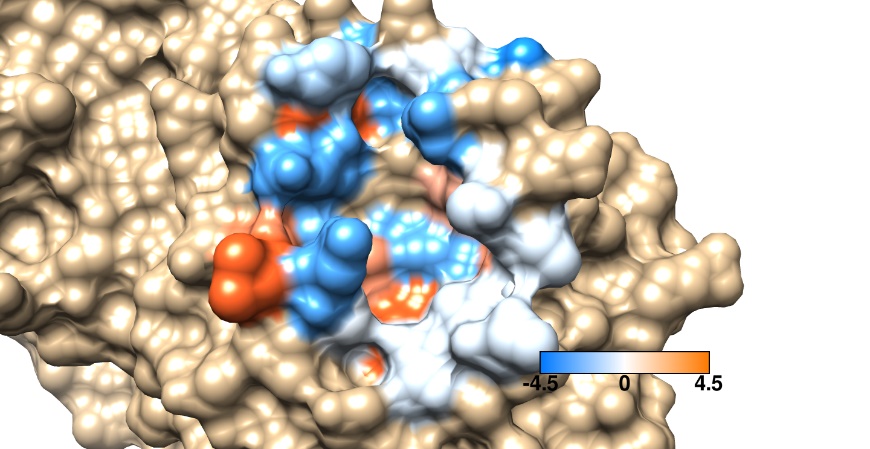  **Figure S2. Active site cavity for SARS-CoV-2 M^pro^ 6YB7 crystal identified with CASTp.** Cavity surface colored according to the hydrophobicity scale of Kyte-Doolittle. |
| --- | --- | --- | --- | --- | --- | --- | --- | --- | --- | --- | --- | --- | --- | --- | --- | --- | --- | --- | --- |


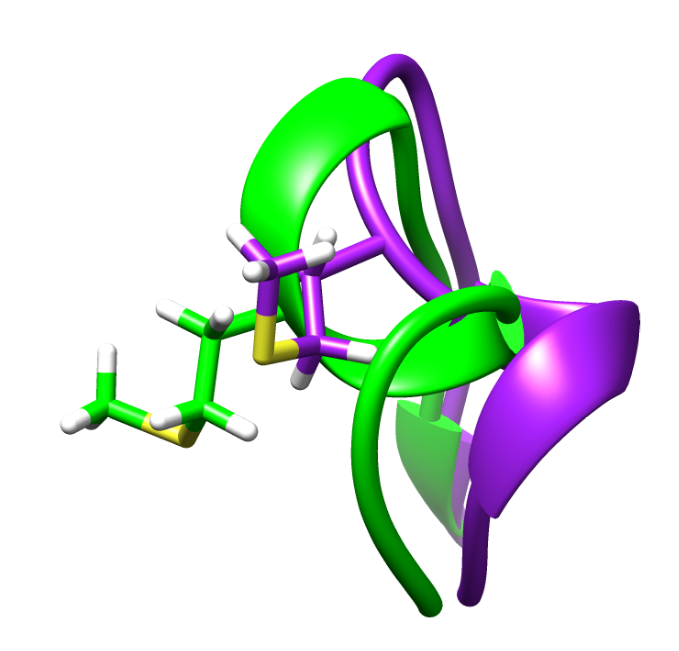


**Figure S3. Met-49 movement and surroundings between the apoprotein 6YB7 (green) and complex 6LU7 (purple).**

| 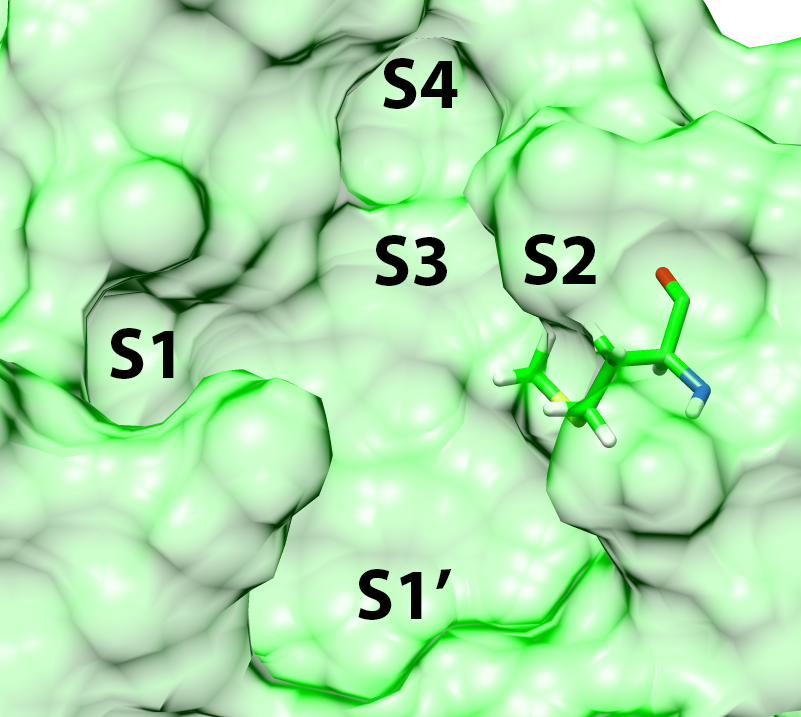  **(A)** | 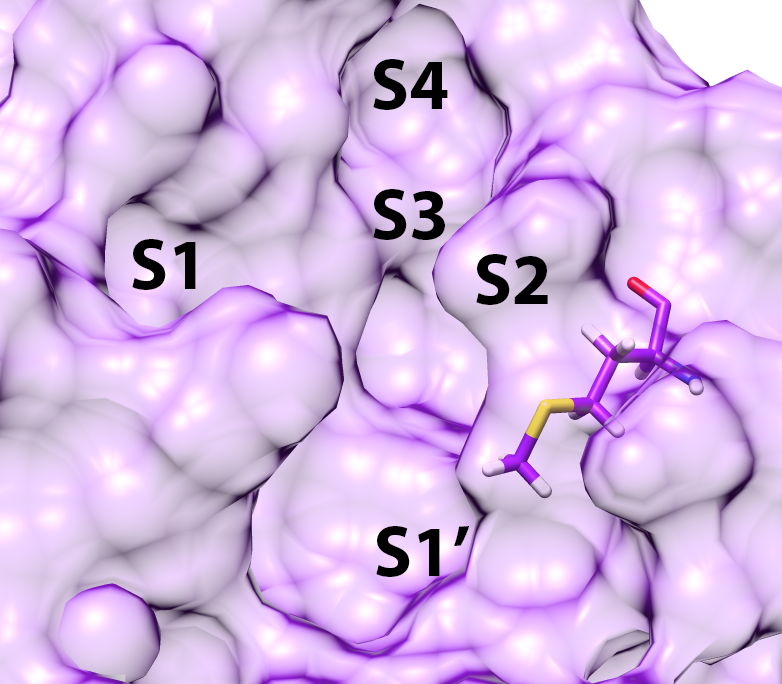  **(B)** |
| --- | --- |

**Figure S4. Active site surface of 6YB7 apoprotein (green) and 6LU7 complex (purple) emphasizing Met-49 residue.**

***Flexible molecular docking.*** In order to perform a flexible molecular docking an important task arose that had to be carried out. This task is related to the fact that the computational cost of flexible molecular docking increases with the degree of flexibility used for the receptor. This means that a defined number of residues must be considered as flexible. To obtain these flexible residues, we created rules that are inspired on literature^32^. The rules are: the residue must 1) be located in a loop, 2) have hydrophobic or hydrophilic contacts with the ligand, 3) have rotamers in its side chain that can exist with a probability close to 0.7, and 4) show movement in several crystals.

In the procedure, the 6LU7 protein was taken and the residues that were located at a distance of 5 Å from the native ligand were found. These residues were subjected to a comparison process to see the number of rules that each residue satisfy. As a result, it was decided to consider the flexibility of the residues: Met-49, Asn-142, Cys-145, Met-165, Asp-187 and Gln-189.

**Table S7. Flexibility rules applied to 6 residues of SARS-CoV-2 main protease.** Y=residue satisfies rule, N=residue does not satisfy rule.

| **Residue** | **Rule 1** | **Rule 2** | **Rule 3** | **Rule 4** |
| --- | --- | --- | --- | --- |
| **Met‑49** | Y | Y | Y | Y |
| **Asn‑142** | Y | Y | Y | Y |
| **Cys‑145** | Y | Y | Y | N |
| **Met‑165** | N | Y | Y | Y |
| **Asp‑187** | Y | Y | Y | N |
| **Gln‑189** | Y | Y | Y | Y |

After obtaining the poses from molecular docking and score the poses with XP Gscore and MM/GBSA protein-ligand binding energy, a visual inspection of the obtained poses was performed. This inspection was considered crucial because the pose scoring ignores the reactivity effects of the catalytic dyad residues. This is due to the fact that the score generated principally considers the non-covalent interactions between protein and ligand. Therefore, in the case of covalent inhibitors there are issues when predicting experimental pose.

To solve this problem, the meaning of two terms used in this research must be clarified, which are known as thermodynamic pose and kinetic pose. The thermodynamic pose refers to the pose with the lowest MM/GBSA protein-ligand binding energy. On the other hand, the kinetic pose is the pose with the lowest MM/GBSA protein-ligand binding energy and with the shortest distance between the nucleophilic residue Cys-145 and the inhibitor warhead. To put these terms into practice, in (**Figure S5**) a visualization of the thermodynamic and kinetic pose of the 0019 inhibitor can be found. In **Figure S5A** it can be seen that in the thermodynamic pose the electrophilic aldehyde group is located far from the Cys-145 residue. Thermodynamic pose has a ΔG of -94.07 kJ/mol. On the other hand, in the kinetic pose (**Figure S5B**) the aldehyde is located near the Cys-145 residue with the carbonyl of the aldehyde interacting through hydrogen bond with the oxyanion hole. This kinetic pose has a ΔG of -91.78 kJ/mol.

| 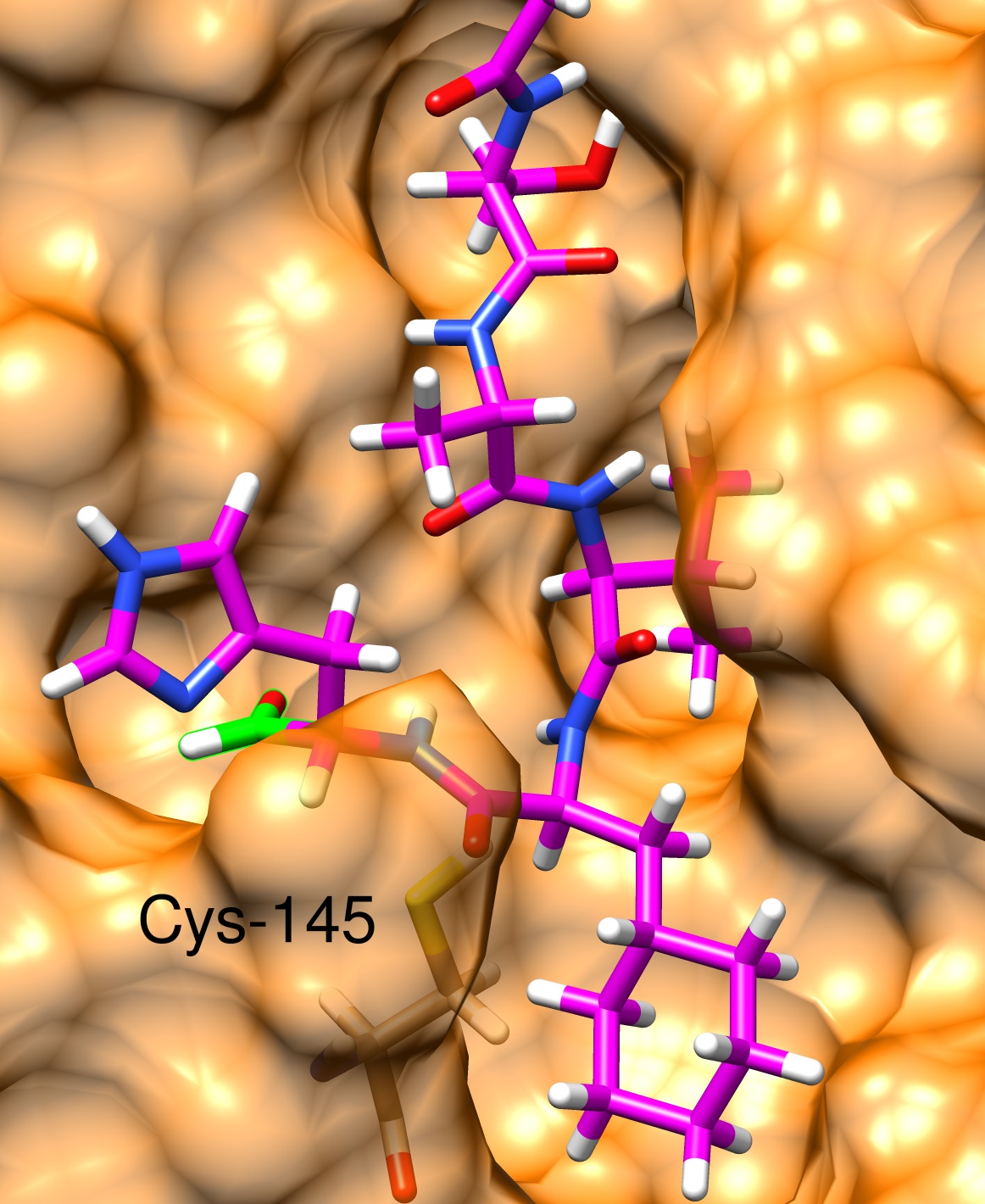  **(A)**  $\Delta G=-94.07 kJ/mol$ |   **0019** | 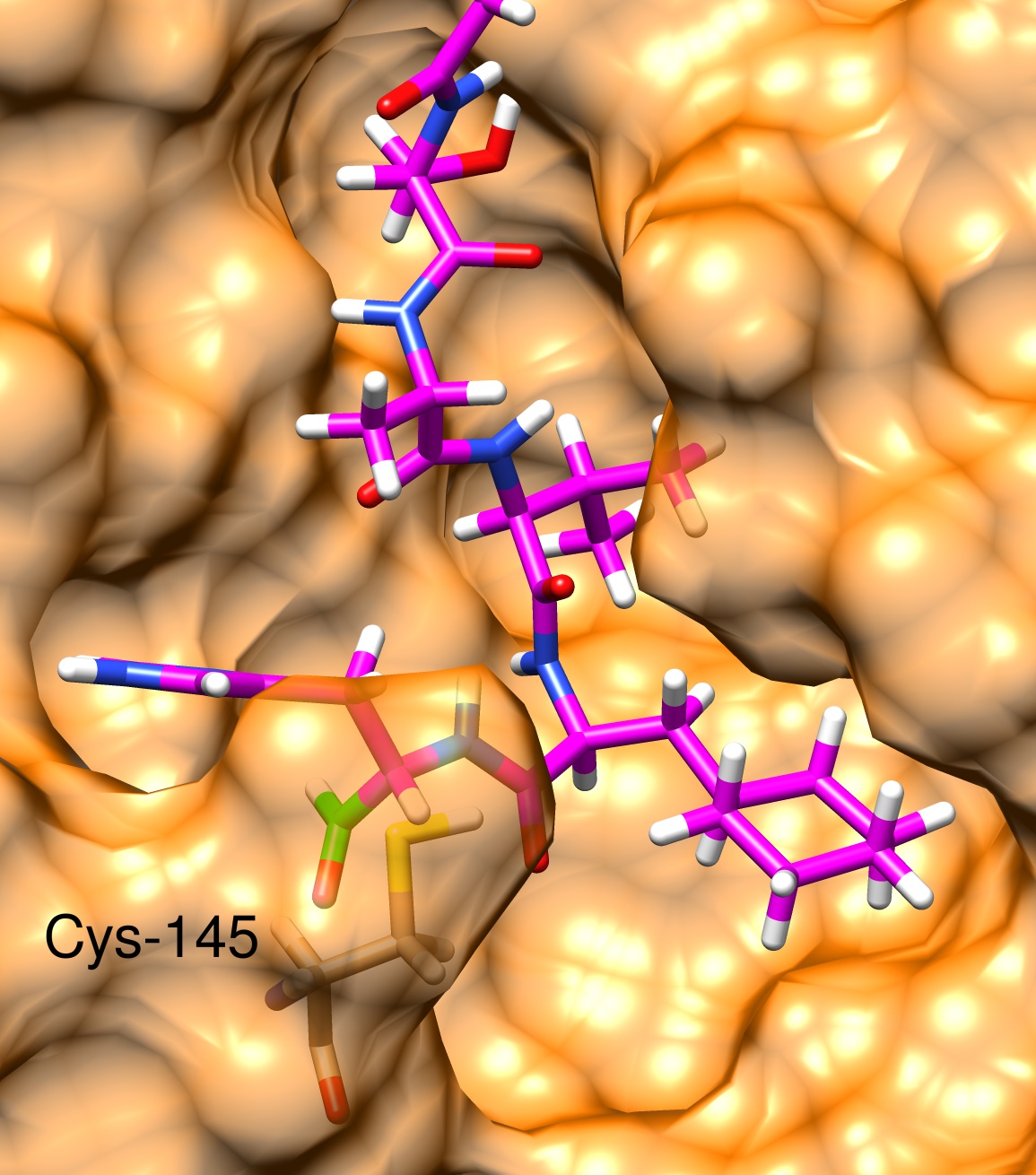  **(B)**  $\Delta G=-91.78 kJ/mol$ |
| --- | --- | --- |

**Figure S5. (A) 0019 thermodynamic pose. (B) 0019 kinetic pose.** Ligand warhead colored in green and red.

In the process of choosing the best pose for the covalent inhibitors, the kinetic pose was chosen over the thermodynamic pose as long as the absolute value of the difference between the ΔG value of the two poses is less than 5 kJ/mol. In the case that the thermodynamic pose has a difference of ΔG (in absolute value) with the kinetic pose greater than 5 kJ/mol, then the thermodynamic pose was chosen as the correct pose. The XP Gscore and ΔG values ​​calculated with MM/GBSA of the best poses of each complex can be found in **Table S8** and **Table S9**.

***Molecular dynamics stability.*** Energy, volume, temperature, and pressure graphs for the MD production phase were generated to verify correct behavior for the 53 simulated systems. The graphs of kinetic and total energy showed oscillations around a constant value. In addition, constant values ​​are observed in the volume graphs. The temperature and pressure graphs oscillated around 310 K and 1 bar. The graphs are available in Supplementary Data 1.

**Figure S6. Steps to generate an affinity ranking.** The MD trajectories of the SARS-CoV-2 M^pro^ protein-ligand dimer complexes were used for a protein-ligand binding free energy MM/PBSA calculation. Since protease is a dimer then a free energy calculation must be performed for each protomer, in this way each protein-ligand system has two free energy values. The value chosen between these two values ​​corresponds to the one with the lowest variation coefficient. Subsequently, the corresponding standard deviation of the chosen value is added and, in this way, free energy values ​​lacking standard deviation are generated. Finally, the free energy values ​​of all the complexes are used to generate the affinity ranking. A SARS-CoV-2 M^pro^ dimer example is showed in ribbons representation, the free energy value with the lowest variation coefficient corresponds to protomer B.

| 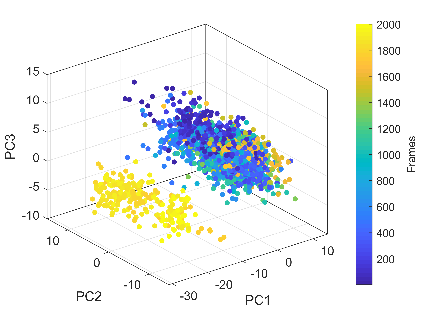 | 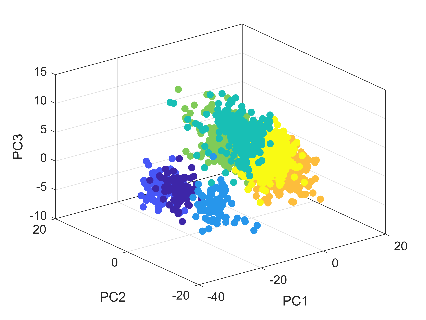 |
| --- | --- |
| **Principal components for 6LU7 ligand atomic coordinates colored by MD time simulation.** | **Principal components for 6LU7 ligand atomic coordinates colored by clusters.** |
| 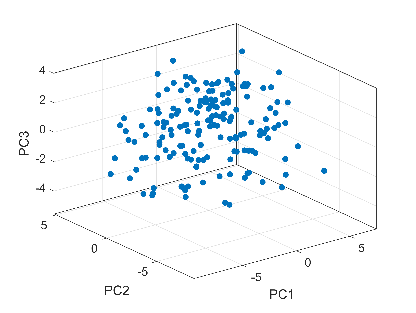 | 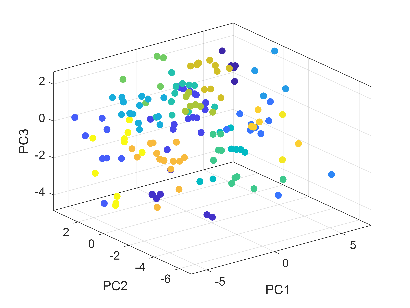 |
| **Principal components for active sites.** | **Principal components for active sites colored by clusters.** |

**Figure S7. Steps for clustering and PCA calculations.** The MD trajectories of the identified actives (**Table S15**) were used to extract the ligand atomic coordinates for the protomer with the lowest variation coefficient from the MM/PBSA calculations. These atomic coordinates went through a principal component analysis (PCA) resulting in three principal components. The principal components were used for a k-means clustering and the ligand poses were classified in ligand-pose-clusters (LPC). For every cluster a centroid was calculated and the nearest MD simulation frame to the centroid was considered as the representative ligand pose of that cluster. As a result, 181 representative structures were extracted. The resulting representative structures went through a principal component analysis for the atomic coordinates of the active site alpha carbons and the three principal components were used for k-means clustering. The 181 representative structures were classified in 18 active-site-conformation-clusters.


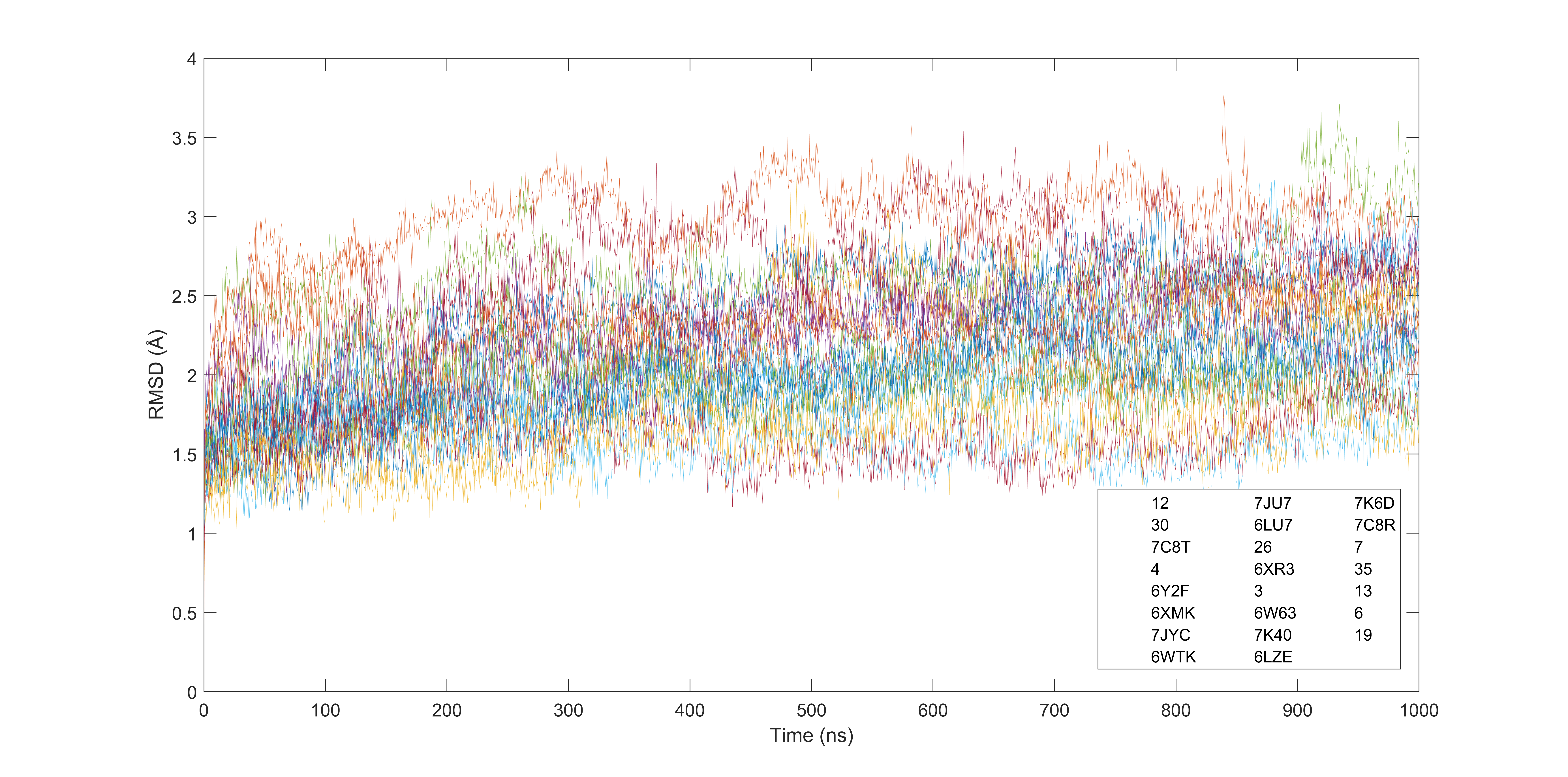


**Figure S8. Superposition of RMSD C-α values of MD trajectories for protein-ligand complexes of active ligands identified with MM/PBSA calculations.**


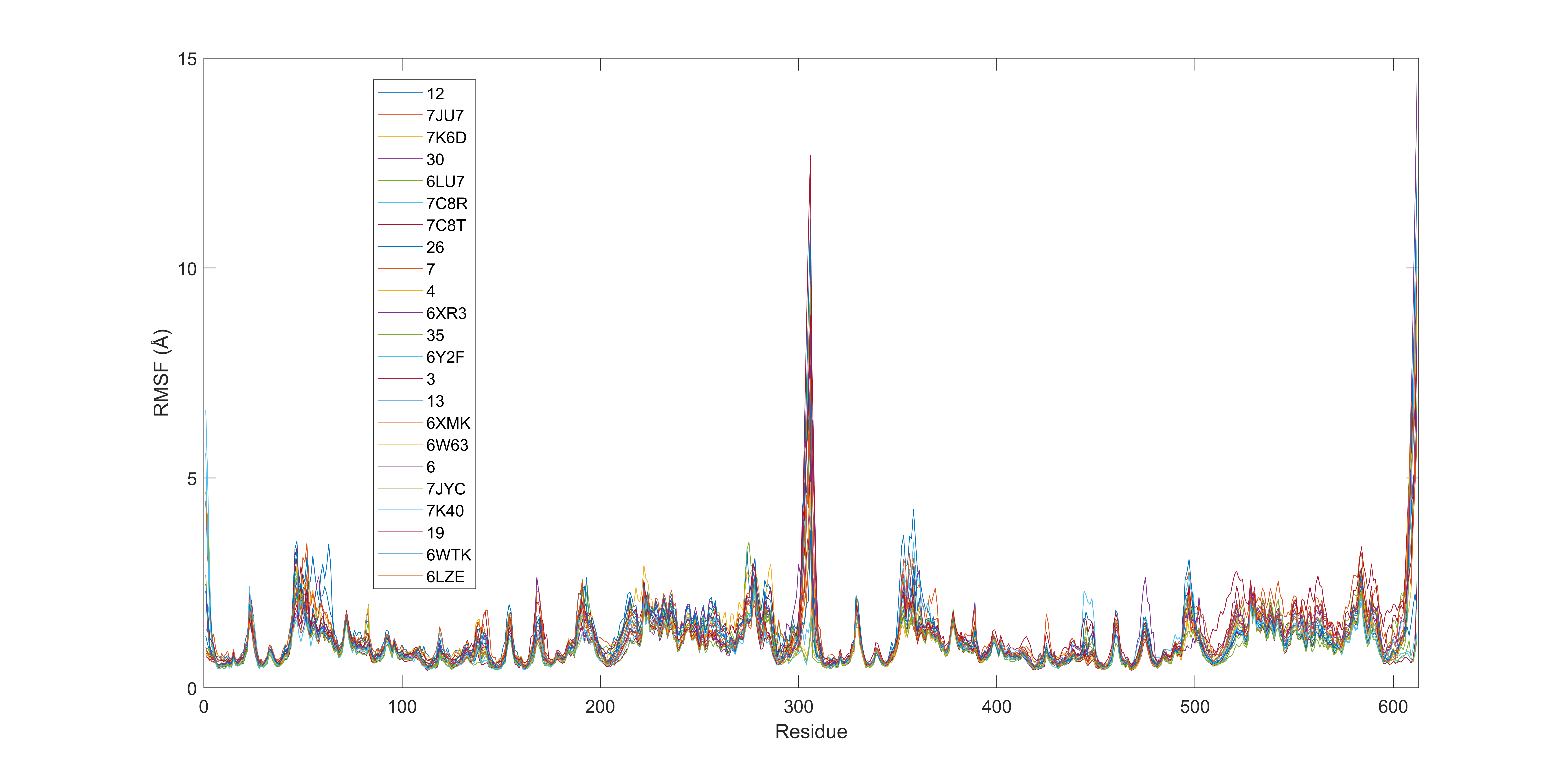


**Figure S9. Superposition of RMSF C-α values of MD trajectories for protein-ligand complexes of active ligands identified with MM/PBSA calculations. Protomer A (Residues 1-306), protomer B (Residues 307-612).**

| 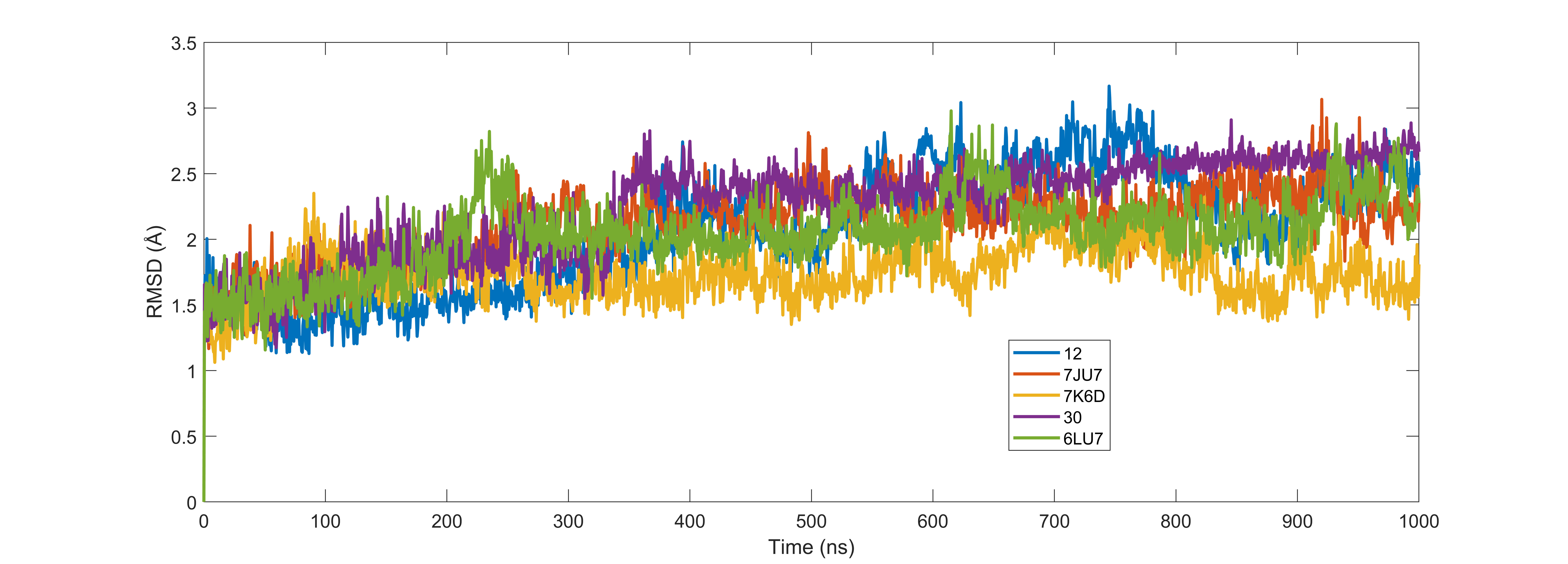 |
| --- |
| 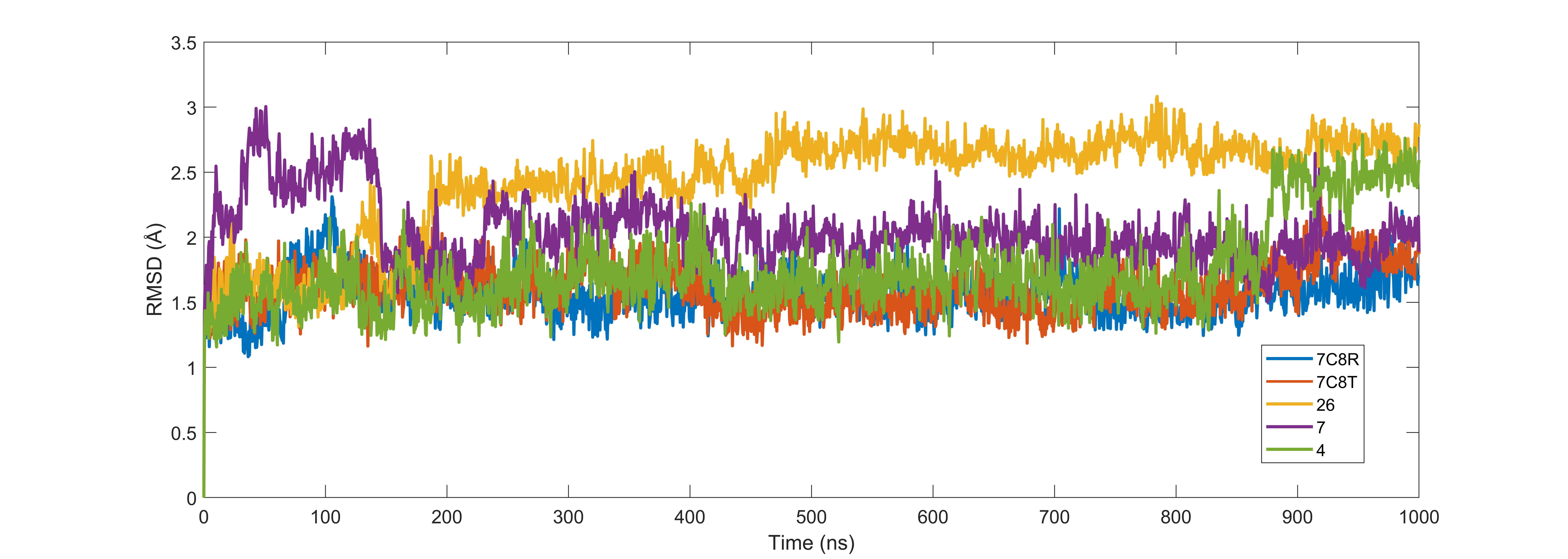 |
| 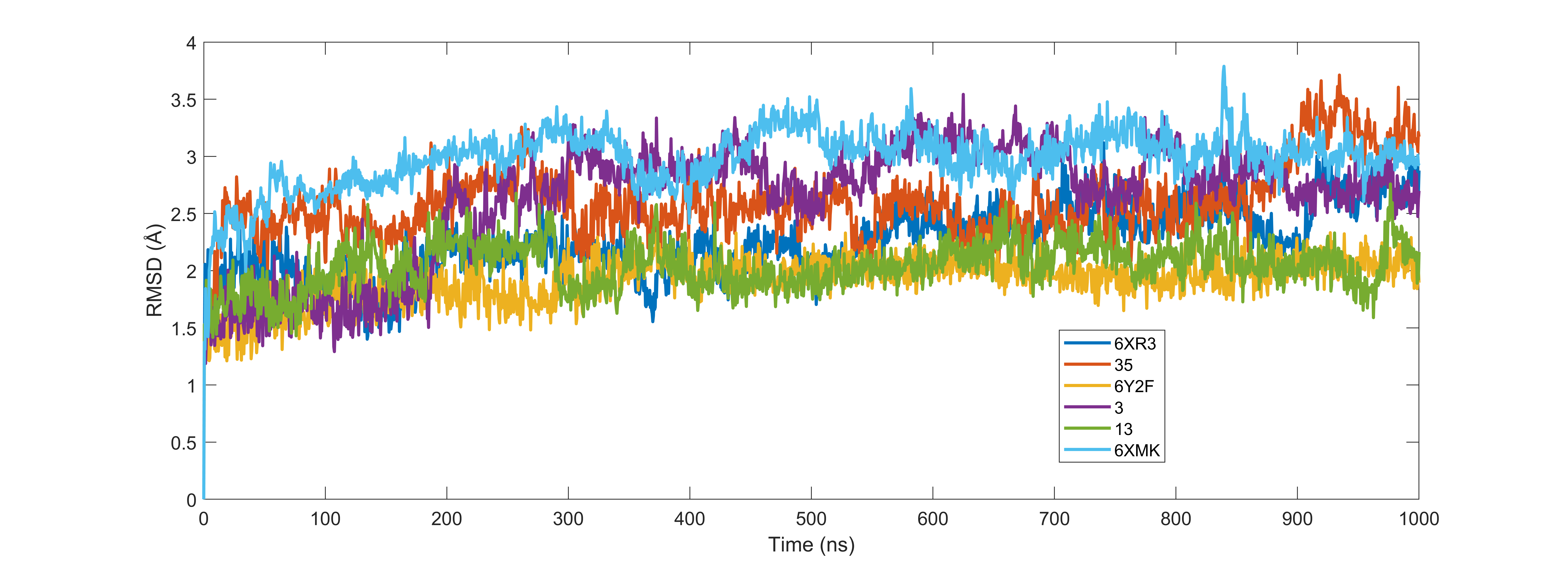 |
| 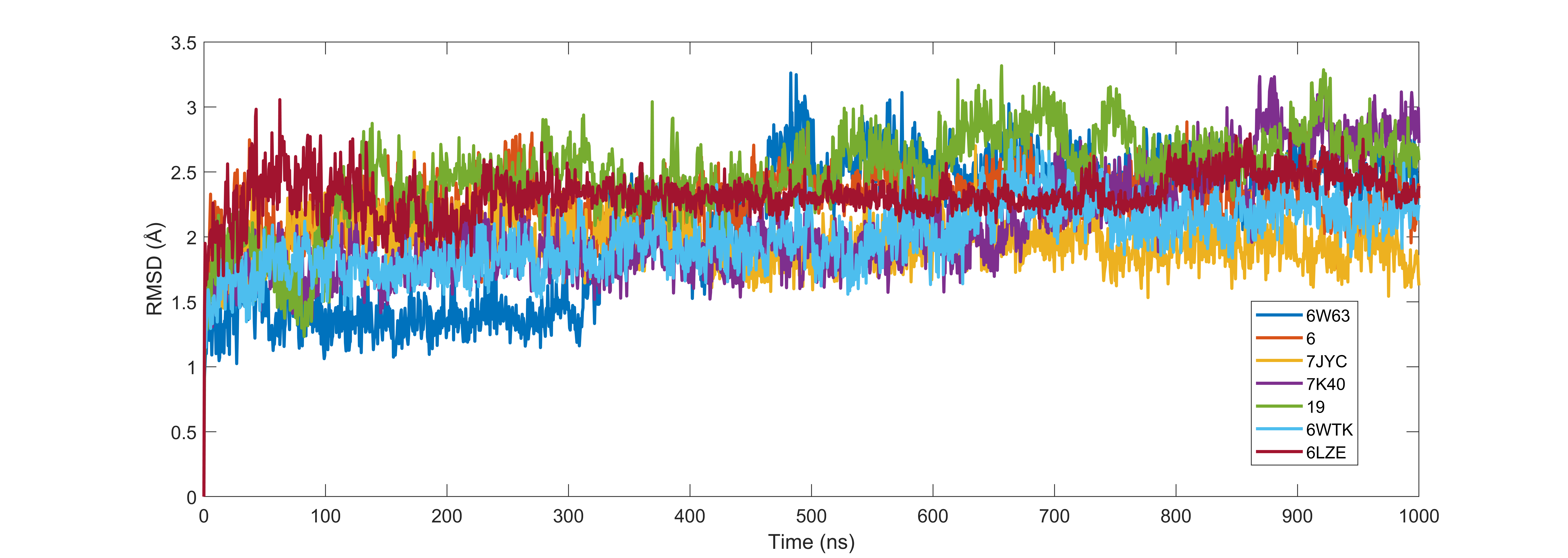 |

**Figure S10. RMSD C-α values of MD trajectories for protein-ligand complexes of active ligands identified with MM/PBSA calculations.**

| 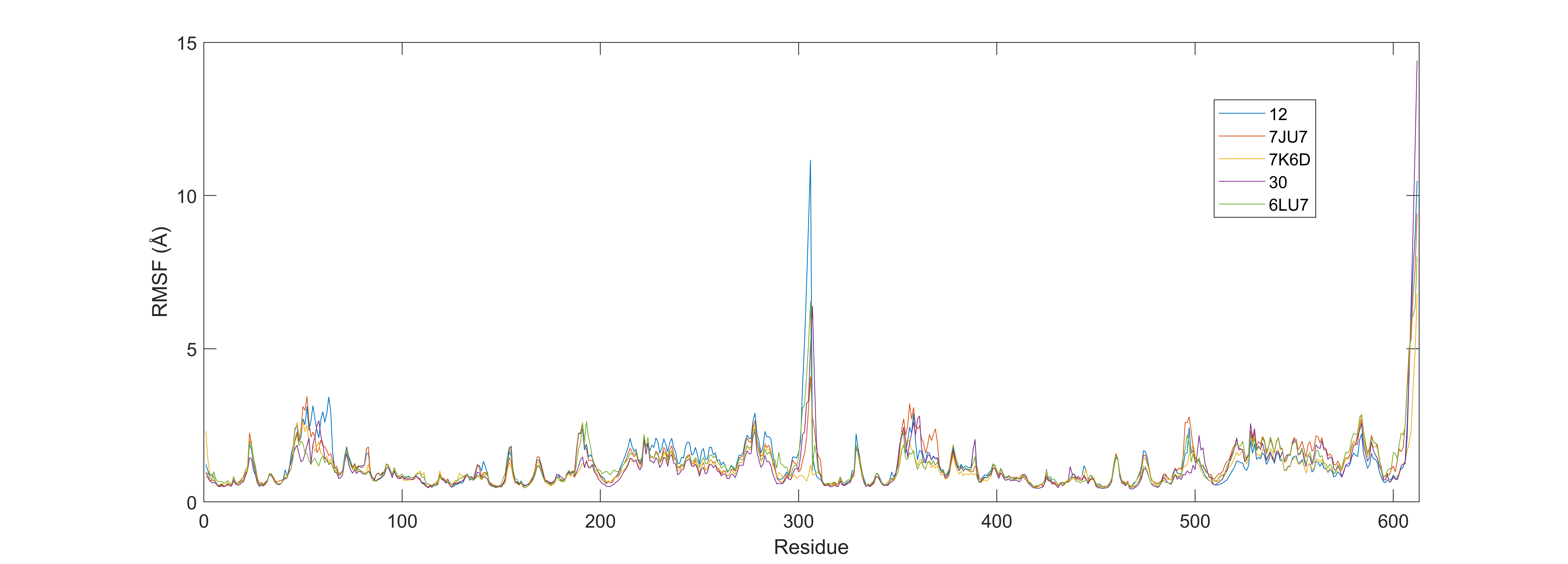 |
| --- |
| 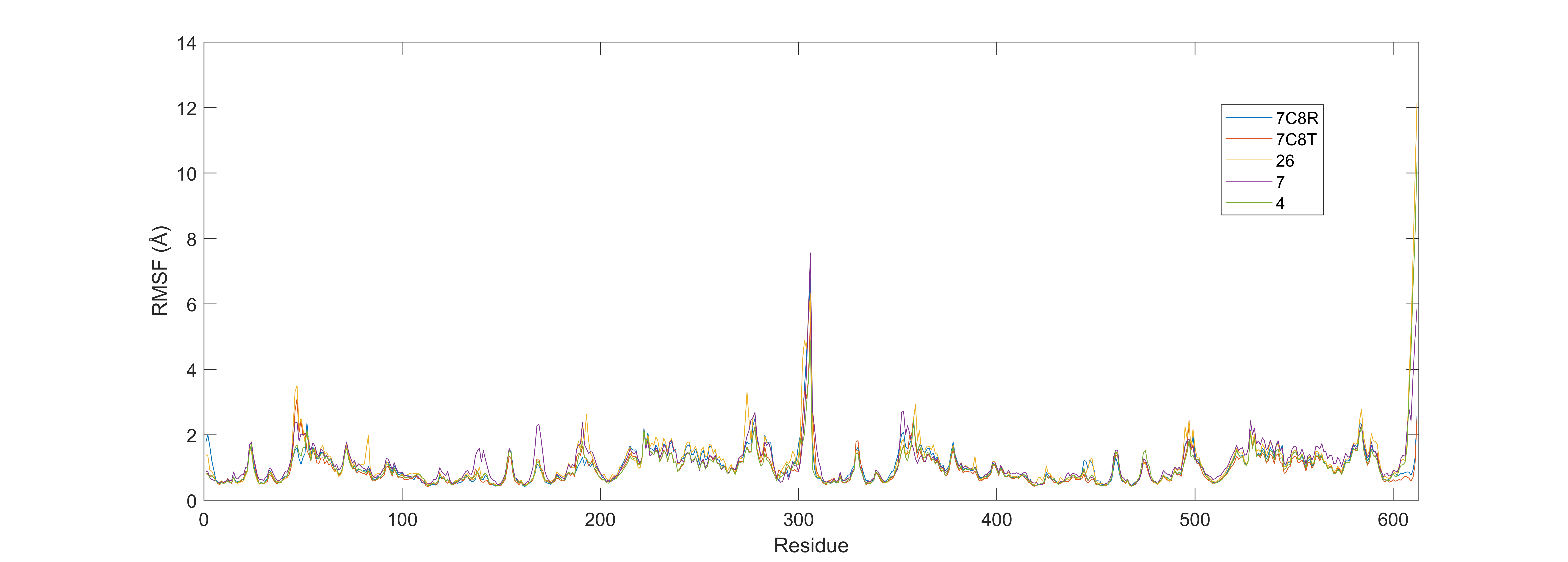 |
| 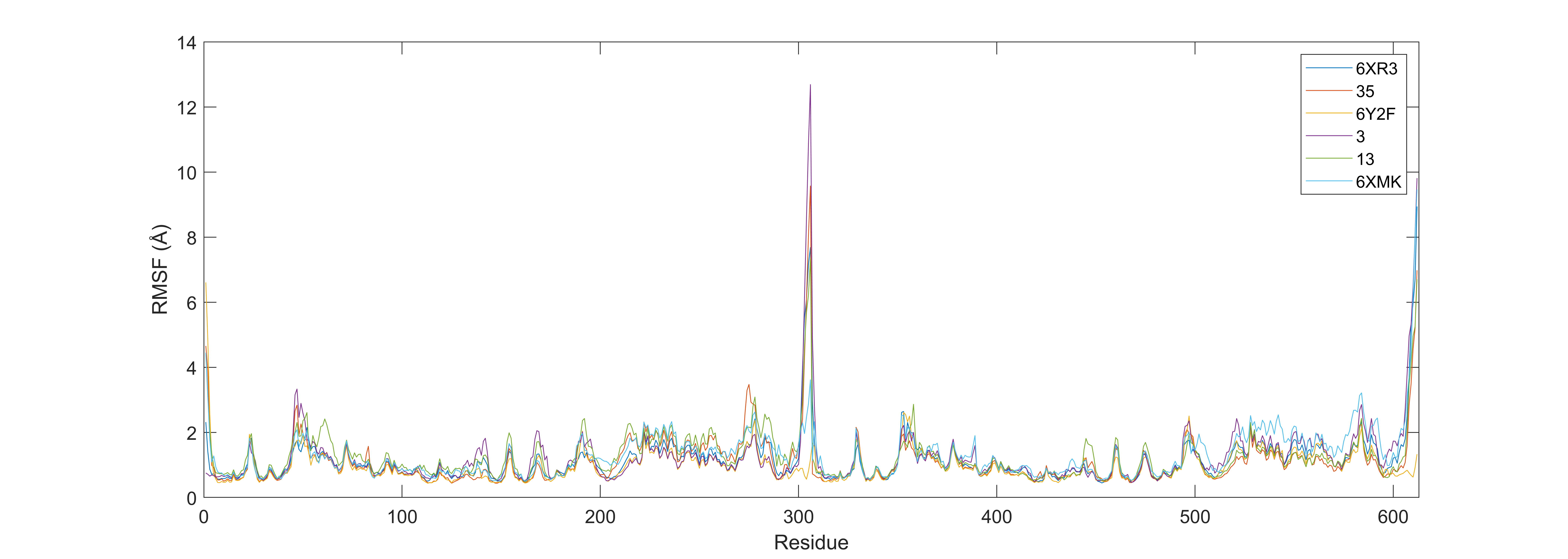 |
| 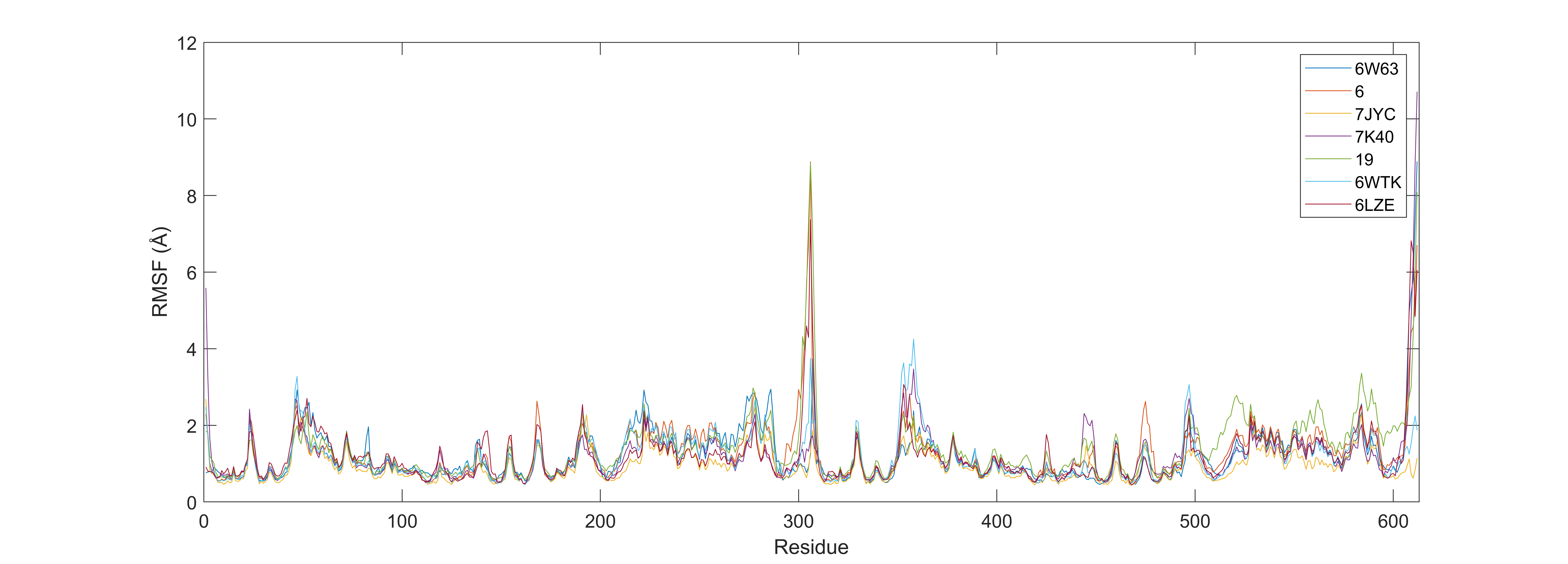 |

**Figure S11. RMSF C-α values of MD trajectories for protein-ligand complexes of active ligands identified with MM/PBSA calculations. Protomer A (Residues 1-306), protomer B (Residues 307-612).**

| **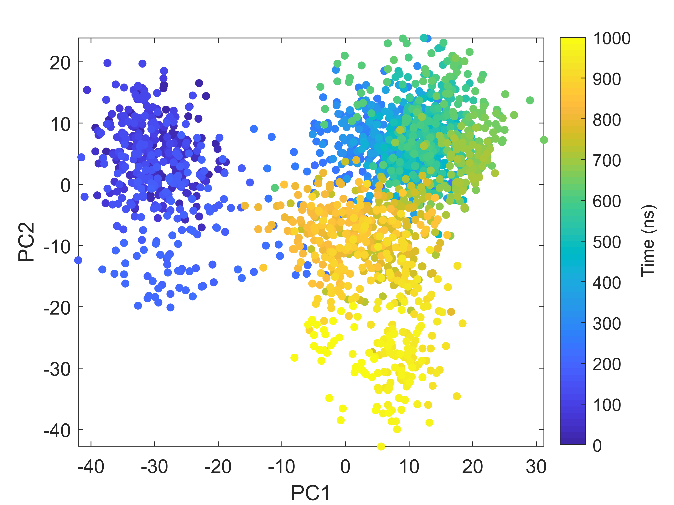** | **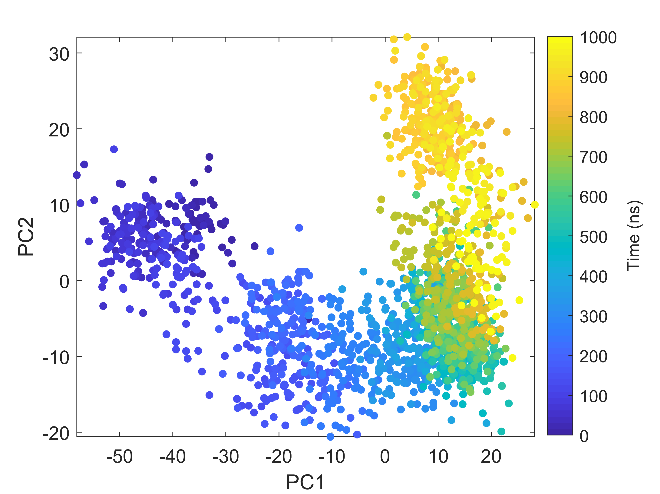** |
| --- | --- |
| **6LU7** | **6LZE** |
| **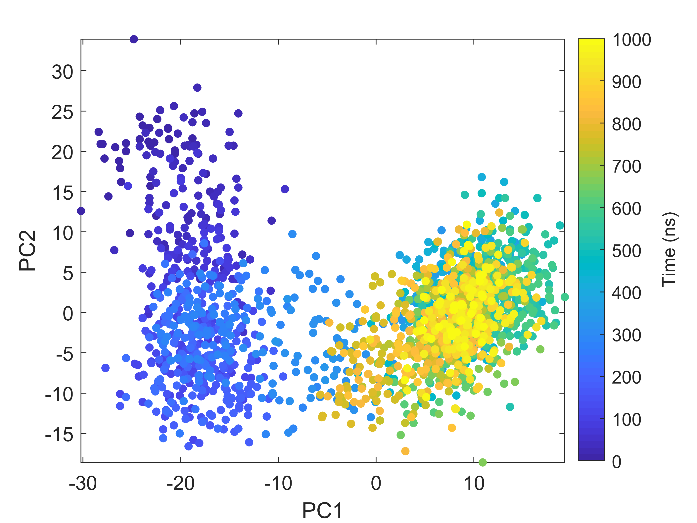** | **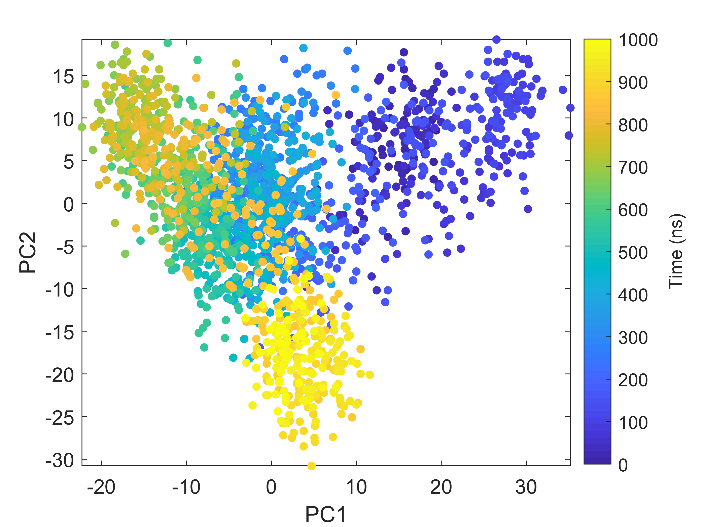** |
| **6Y2F** | **7K6D** |

**Figure S12. Principal components analysis graph (PC1 (principal component 1) vs PC2 (principal component 2)) for C-α of MD trajectories for 6LU7, 6LZE, 6Y2F and 7K6D complexes.**

| **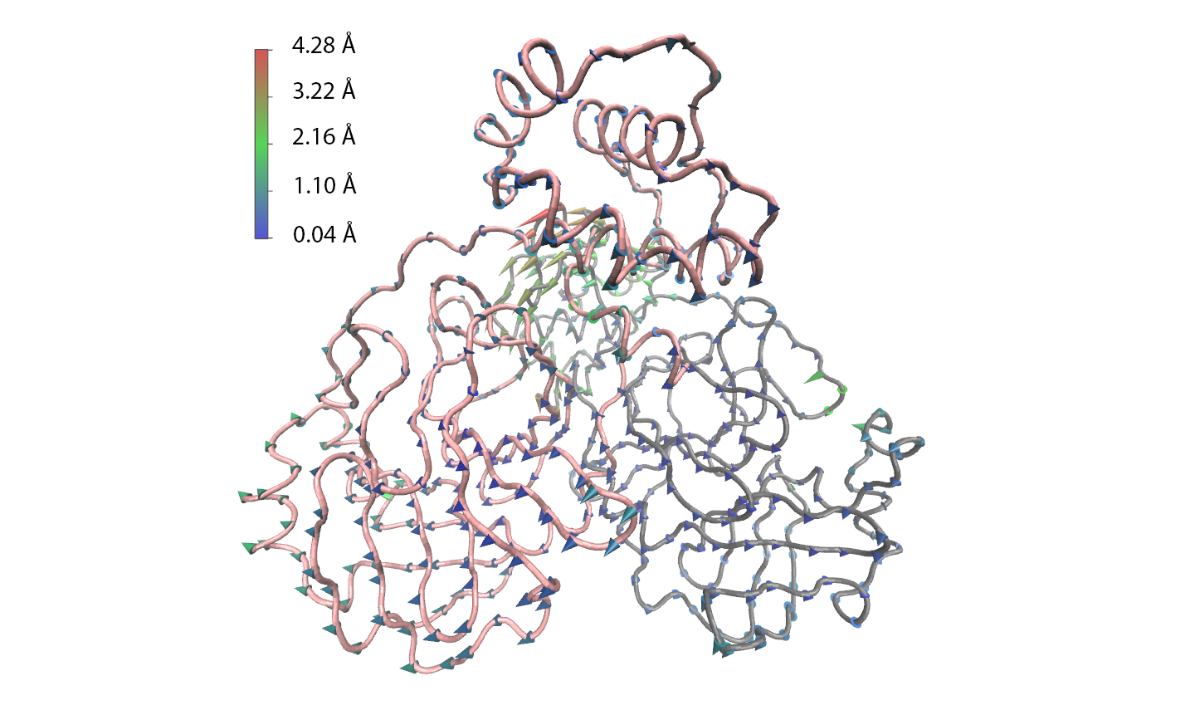** | **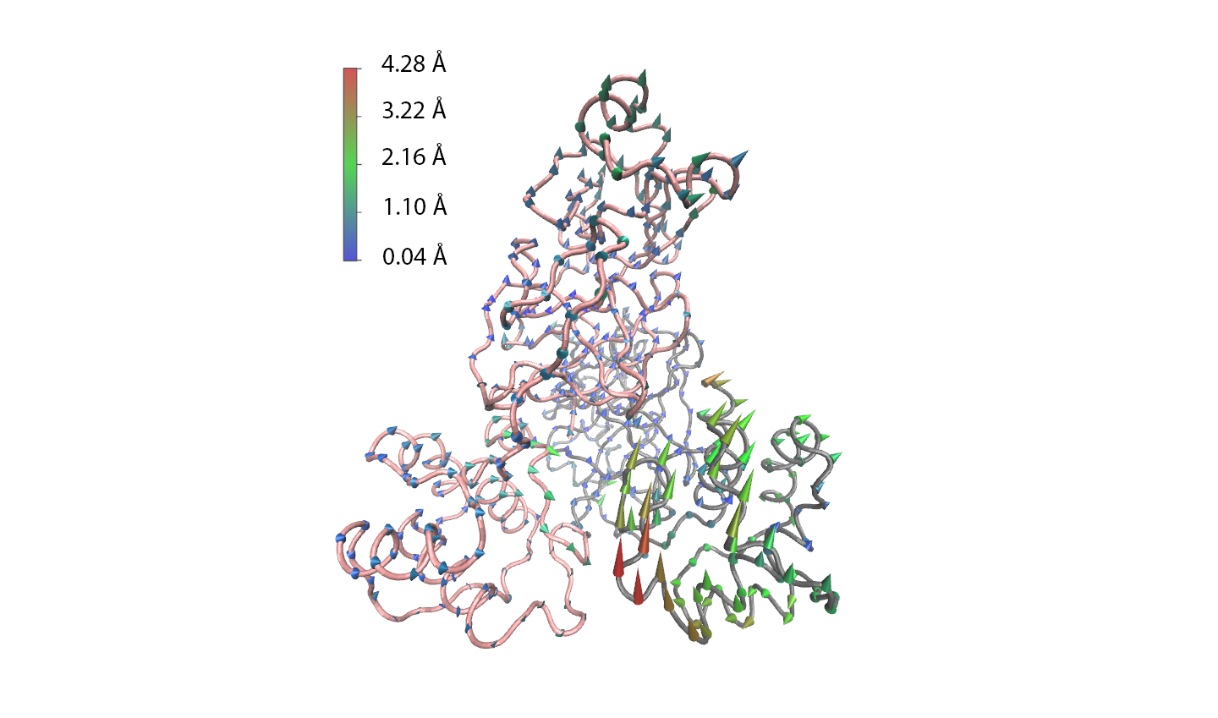** |
| --- | --- |
| **PC1** | |
| **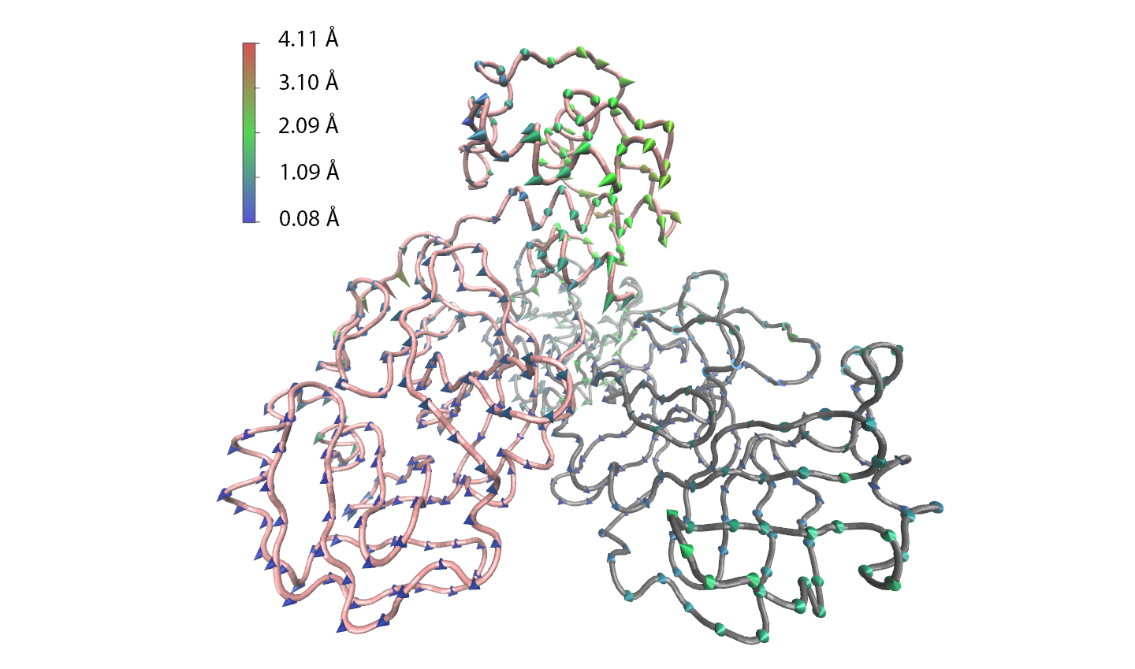** | **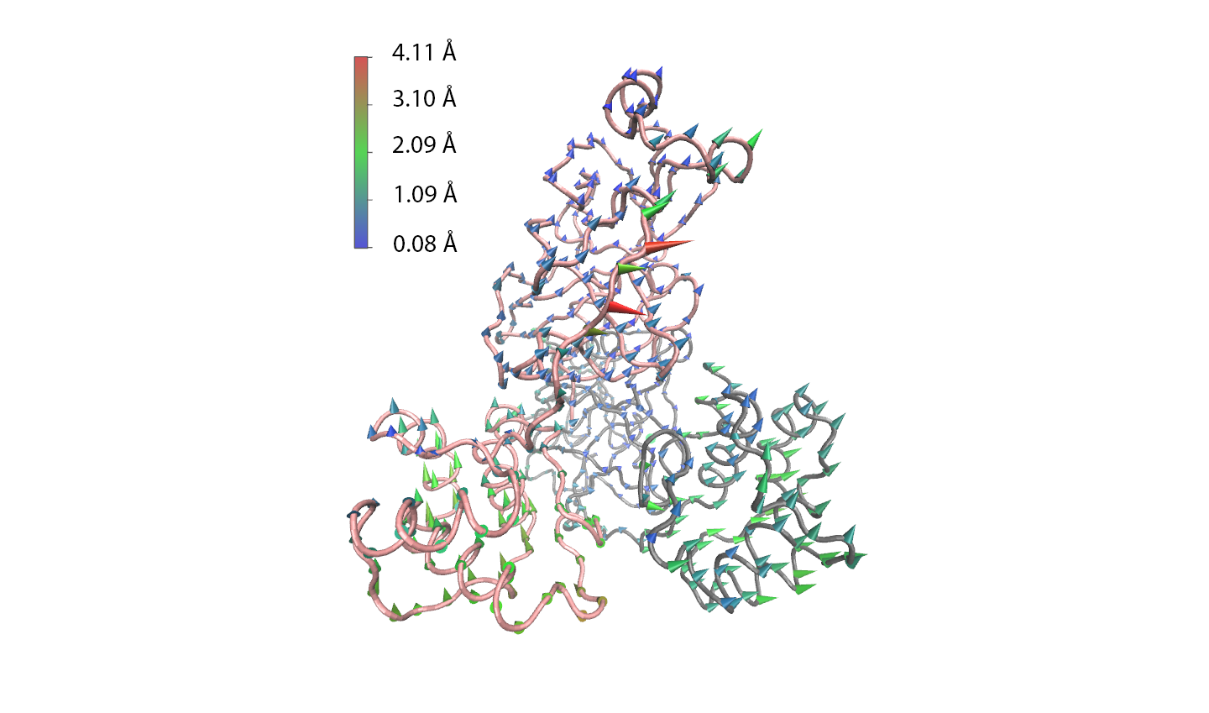** |
| **PC2** | |

**Figure S13. Porcupine plot of protein movements corresponding to PC1 and PC2 for C-α of MD trajectories for 6LU7 complex.**

| 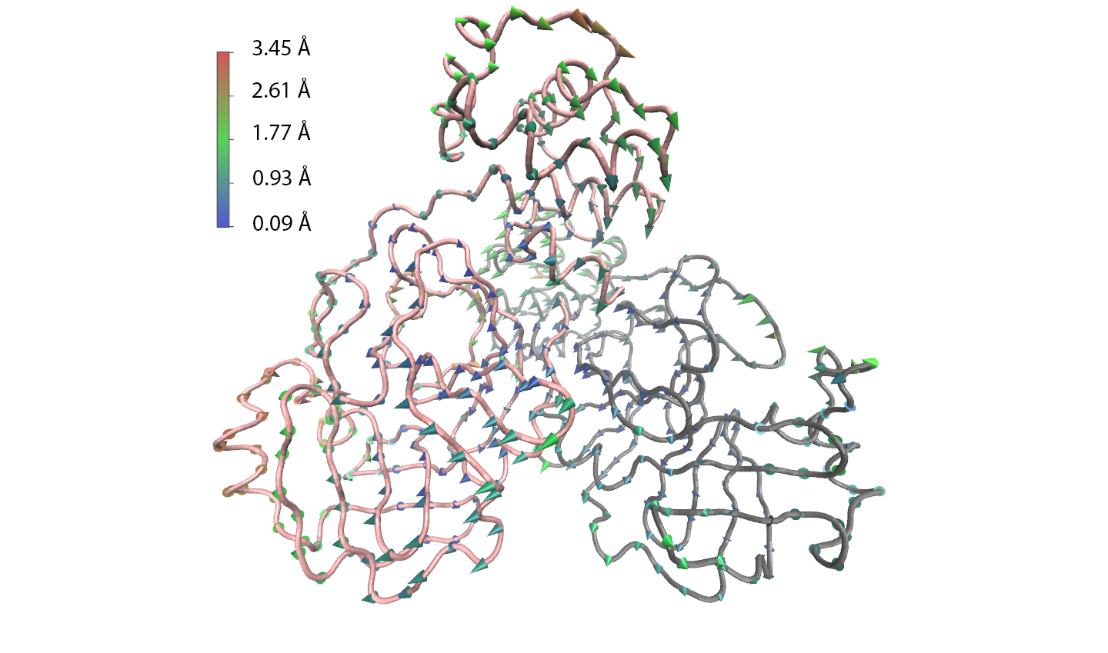 | 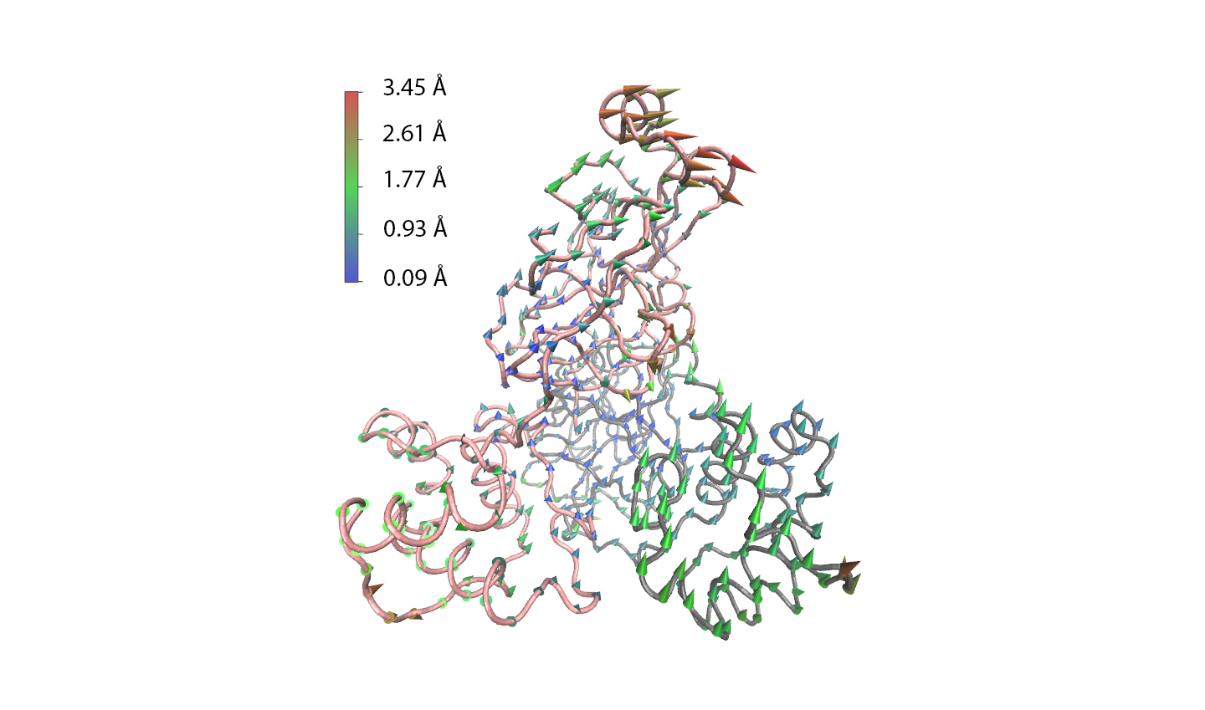 |
| --- | --- |
| **PC1** | |
| 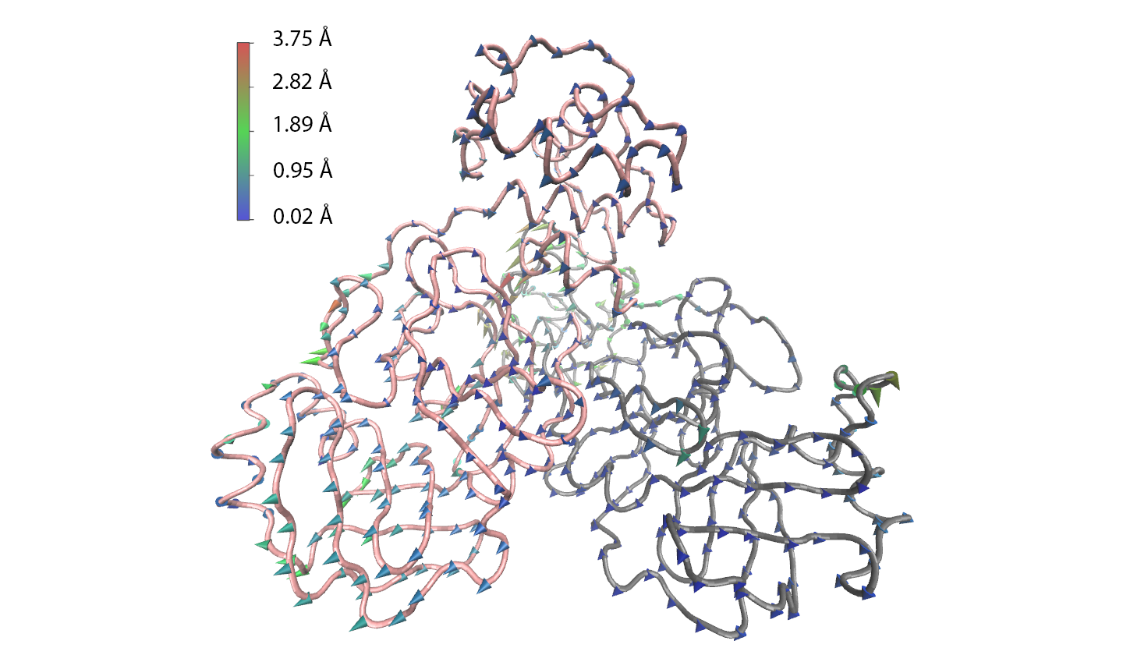 | 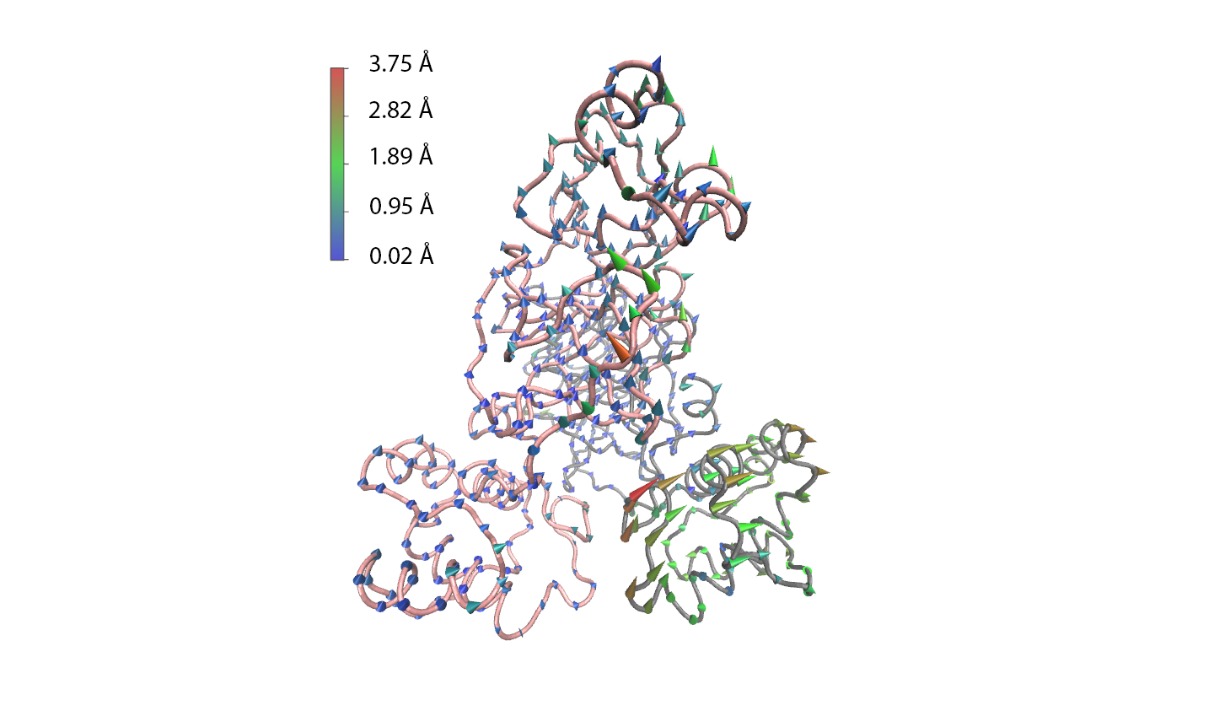 |
| **PC2** | |

**Figure S14. Porcupine plot of protein movements corresponding to PC1 and PC2 for C-α of MD trajectories for 6LZE complex.**

| 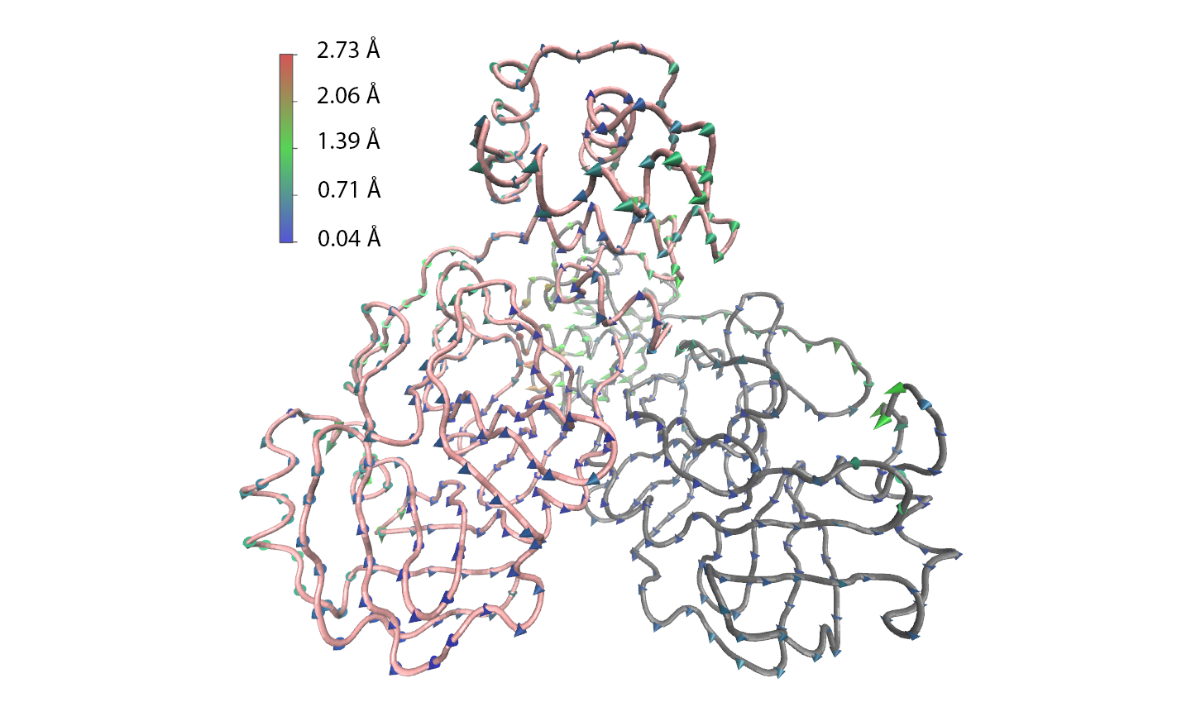 | 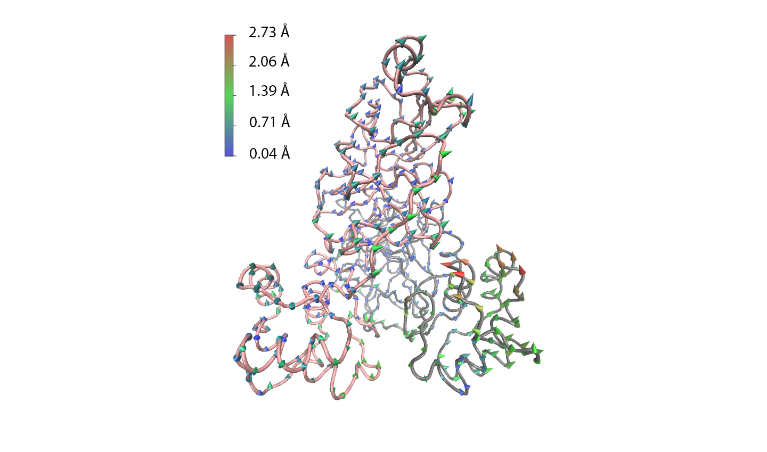 |
| --- | --- |
| **PC1** | |
| 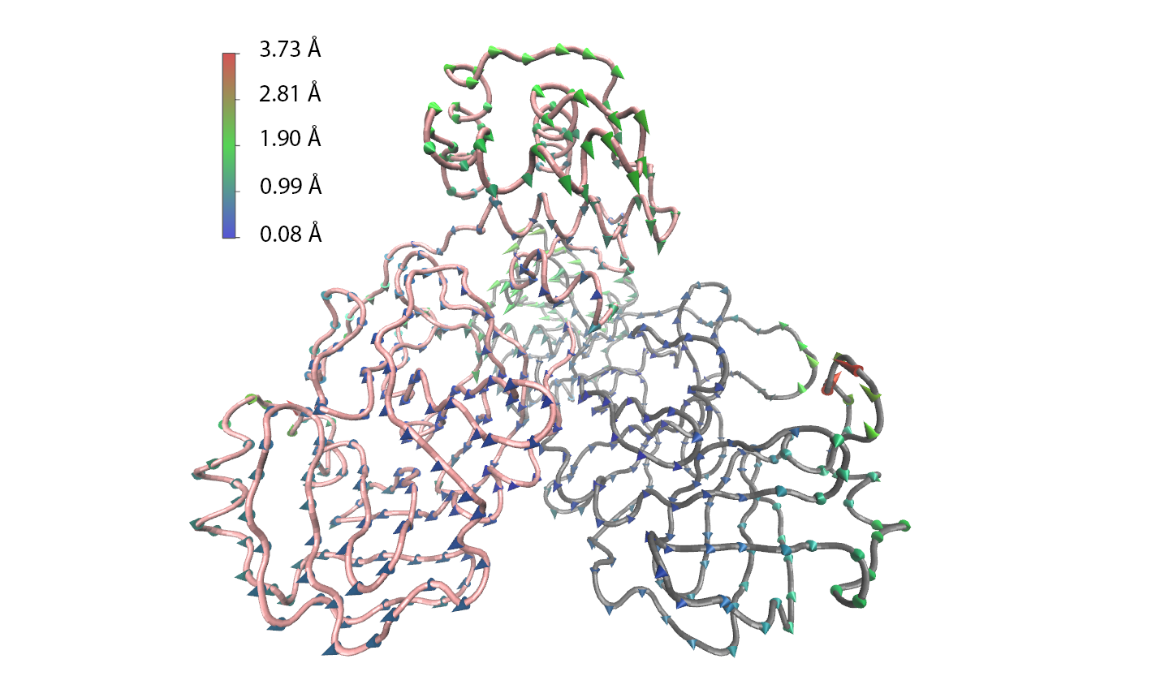 | 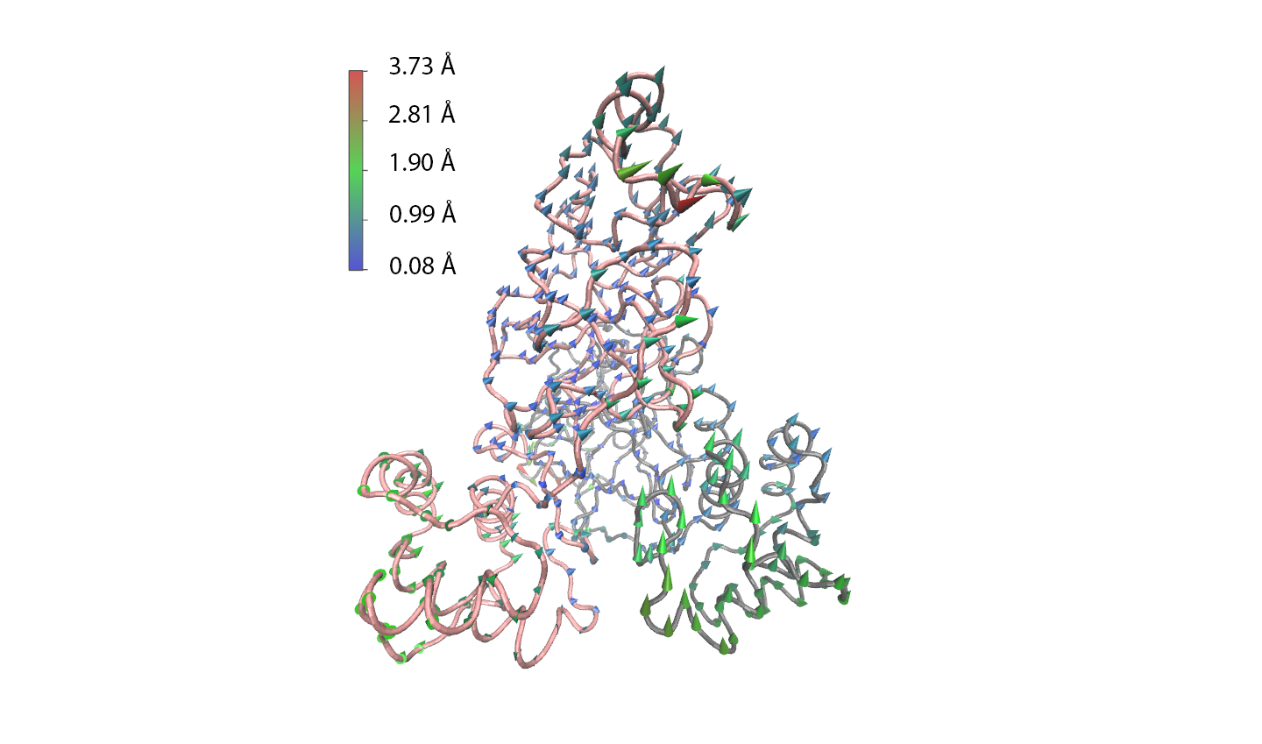 |
| **PC2** | |

**Figure S15. Porcupine plot of protein movements corresponding to PC1 and PC2 for C-α of MD trajectories for 6Y2F complex.**

| 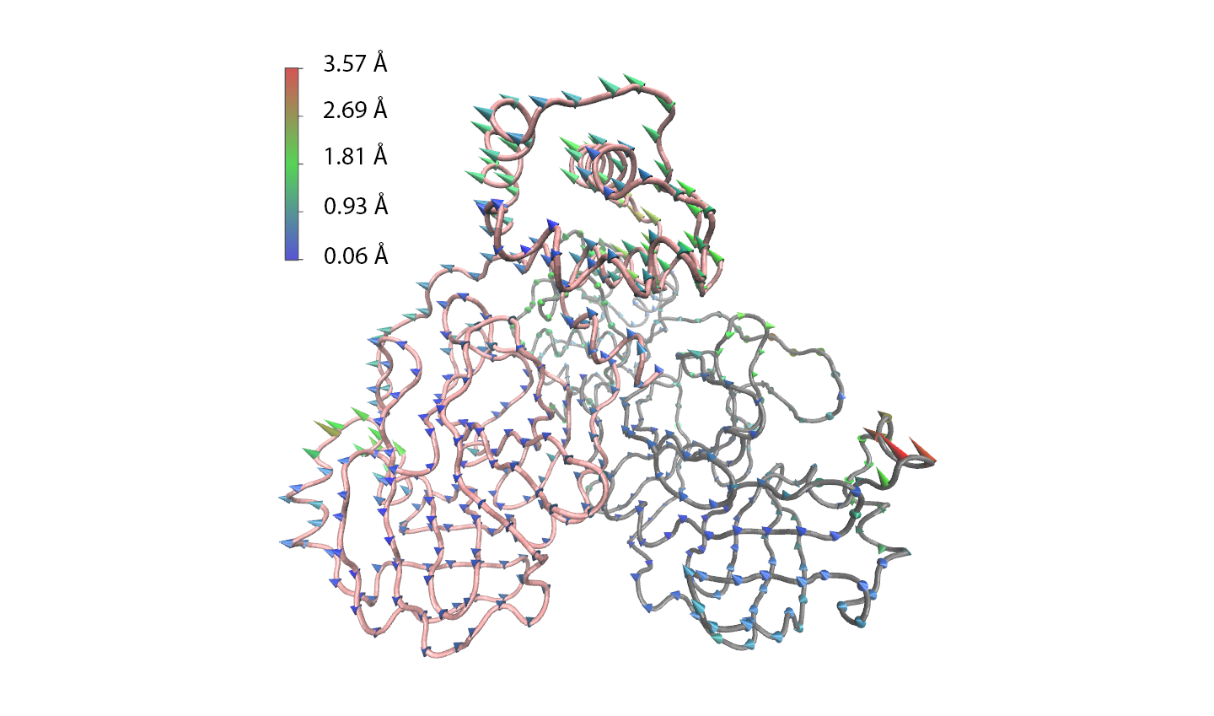 | 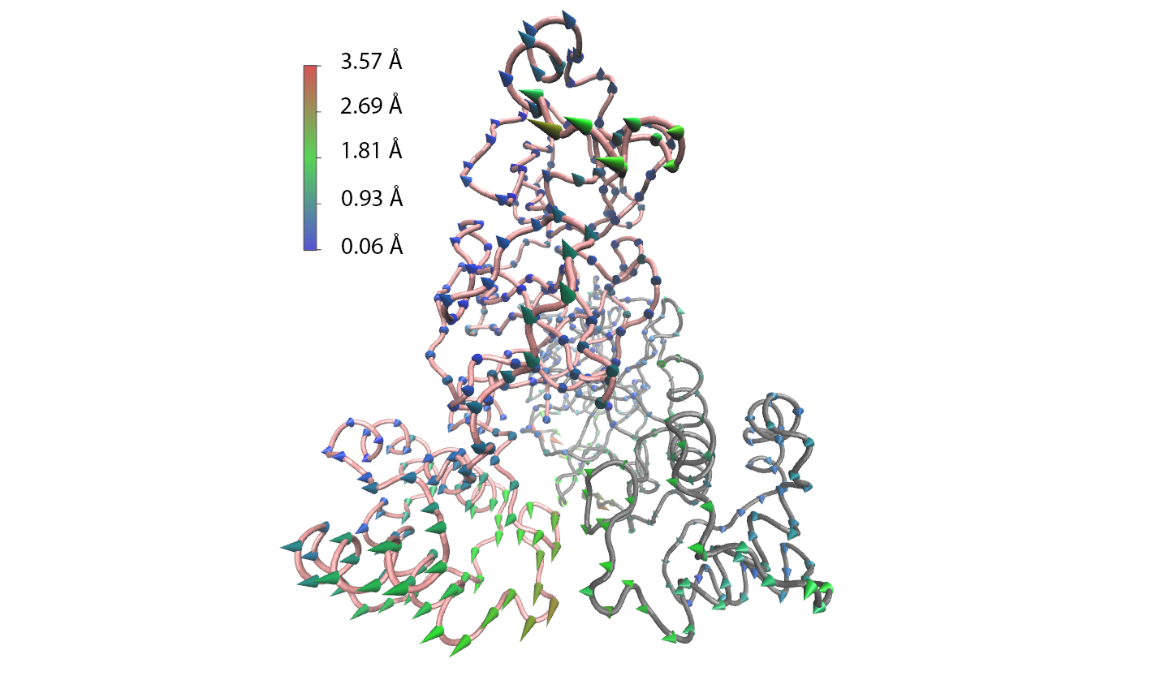 |
| --- | --- |
| **PC1** | |
| 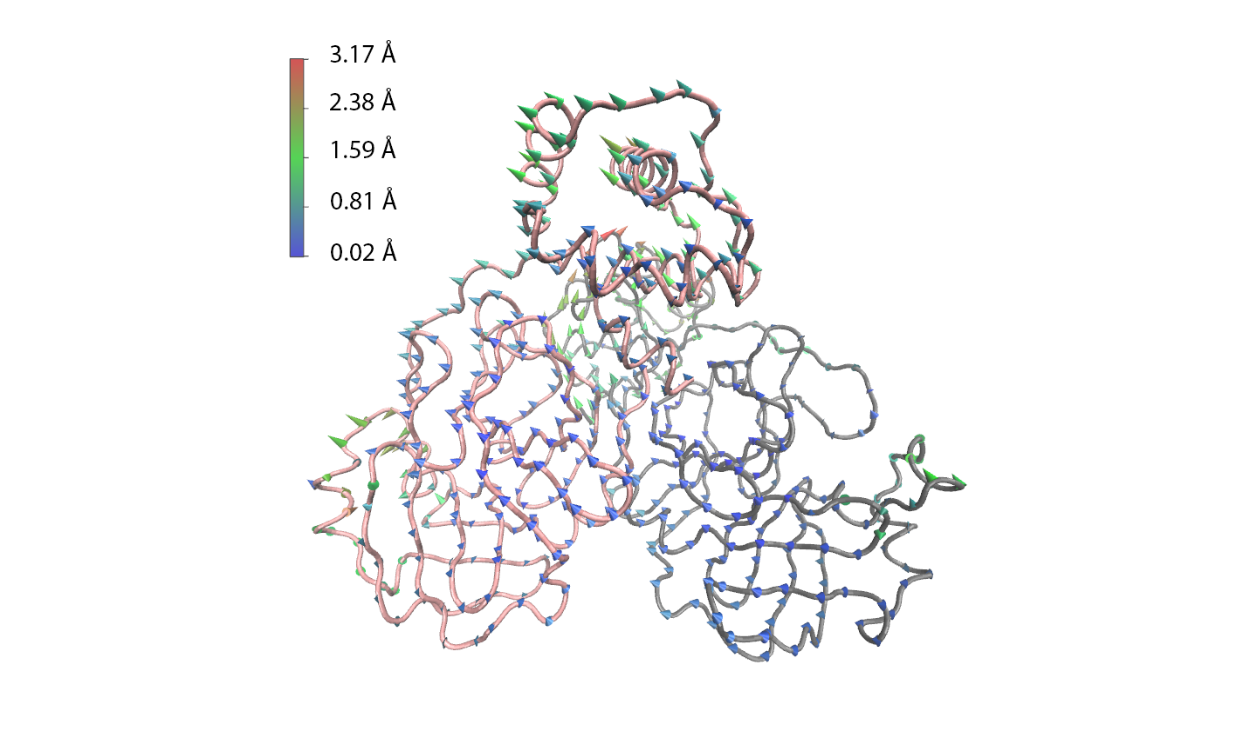 | 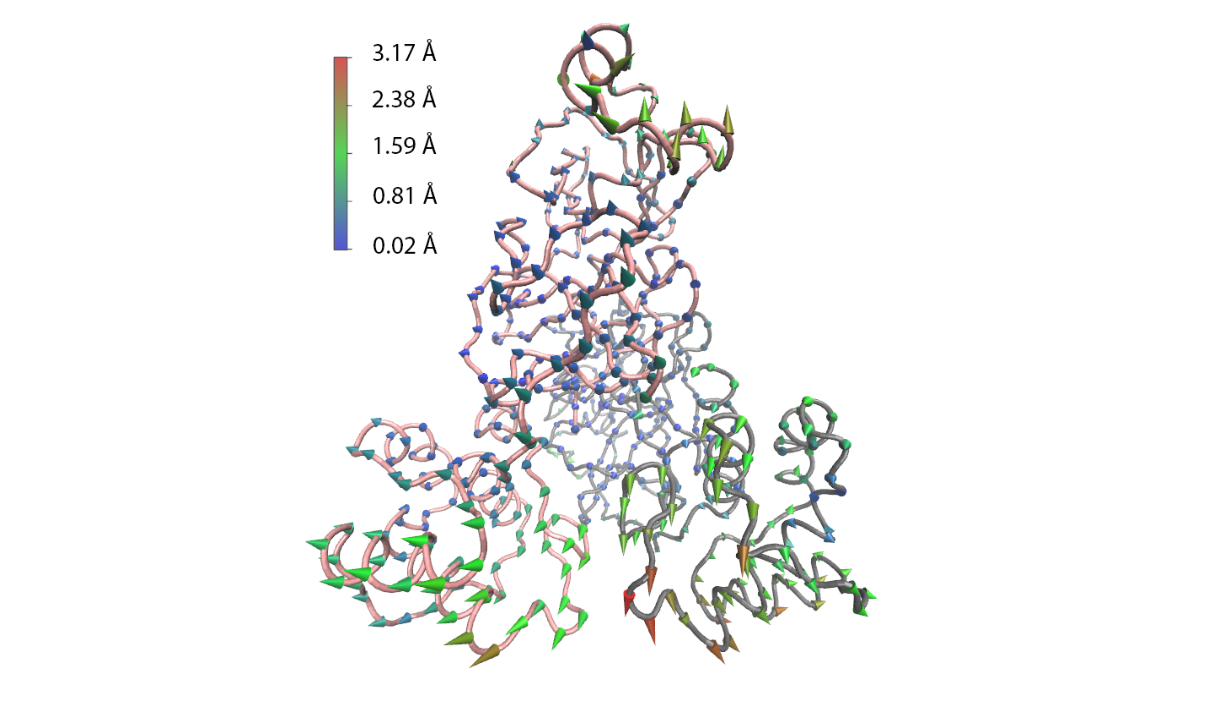 |
| **PC2** | |

**Figure S16. Porcupine plot of protein movements corresponding to PC1 and PC2 for C-α of MD trajectories for 7K6D complex.**

***N-finger H-bond analysis.*** Regarding hydrogen bond analysis, interactions of residues Ser-1 and Arg-4 (**Figure S17**) were inspected for some complexes (**Figure S18, S19, S20, S21**). These residues belong to the N-finger region, which have particular relevance for the interaction between protomers and M^pro^ function^33,34^. While Ser-1 maintained H-bonds with the side-chain of Glu-166 and the backbone of Phe-140 along the MD simulation, Arg-4 was observed to be H-bonded with the side-chain of Glu-290 and Lys-137, as well as the backbone of Gln-127. In some situations, these H-bonds were lost for one protomer but retained in the other protomer, indicating an asymmetric behavior of the protomers concerning hydrogen bonding. Also, H-bond interactions with Ser-1 were observed to be more unstable compared to Arg-4. One reason is the side-chain of Glu-166, which switches from interacting with Ser-1 to interacting with the ligand. This result is in concordance with other studies in which it was found that residues 1-3 are prone to lose hydrogen bonding interactions^35^.

| 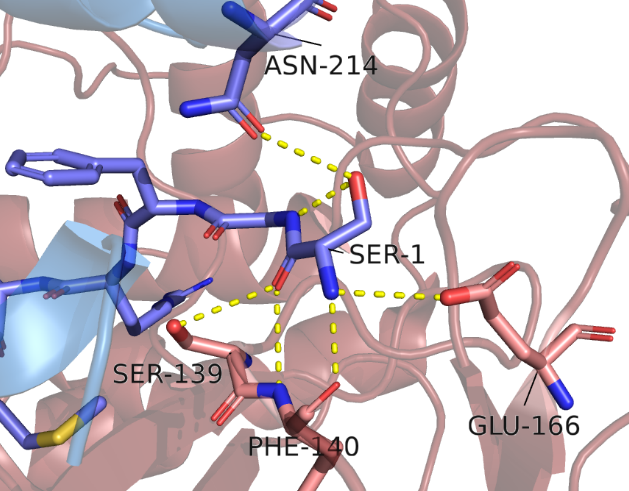  **(A)** | 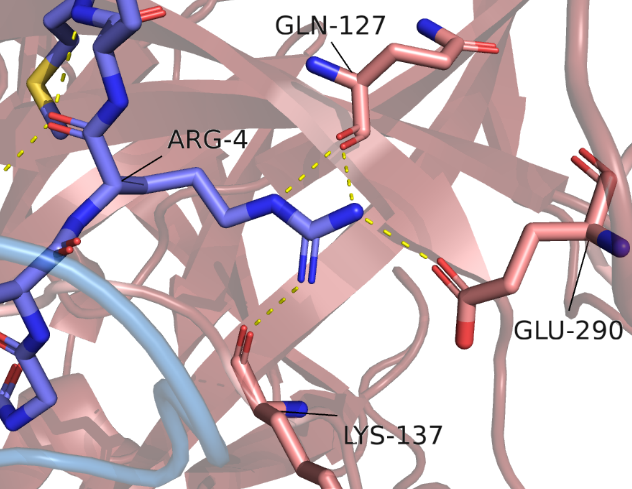  **(B)** |
| --- | --- |

**Figure S17. N-finger interactions of Ser-1 and Arg-4 residues for 1UK3 protein crystal.** Hydrogen bonds in yellow.

| **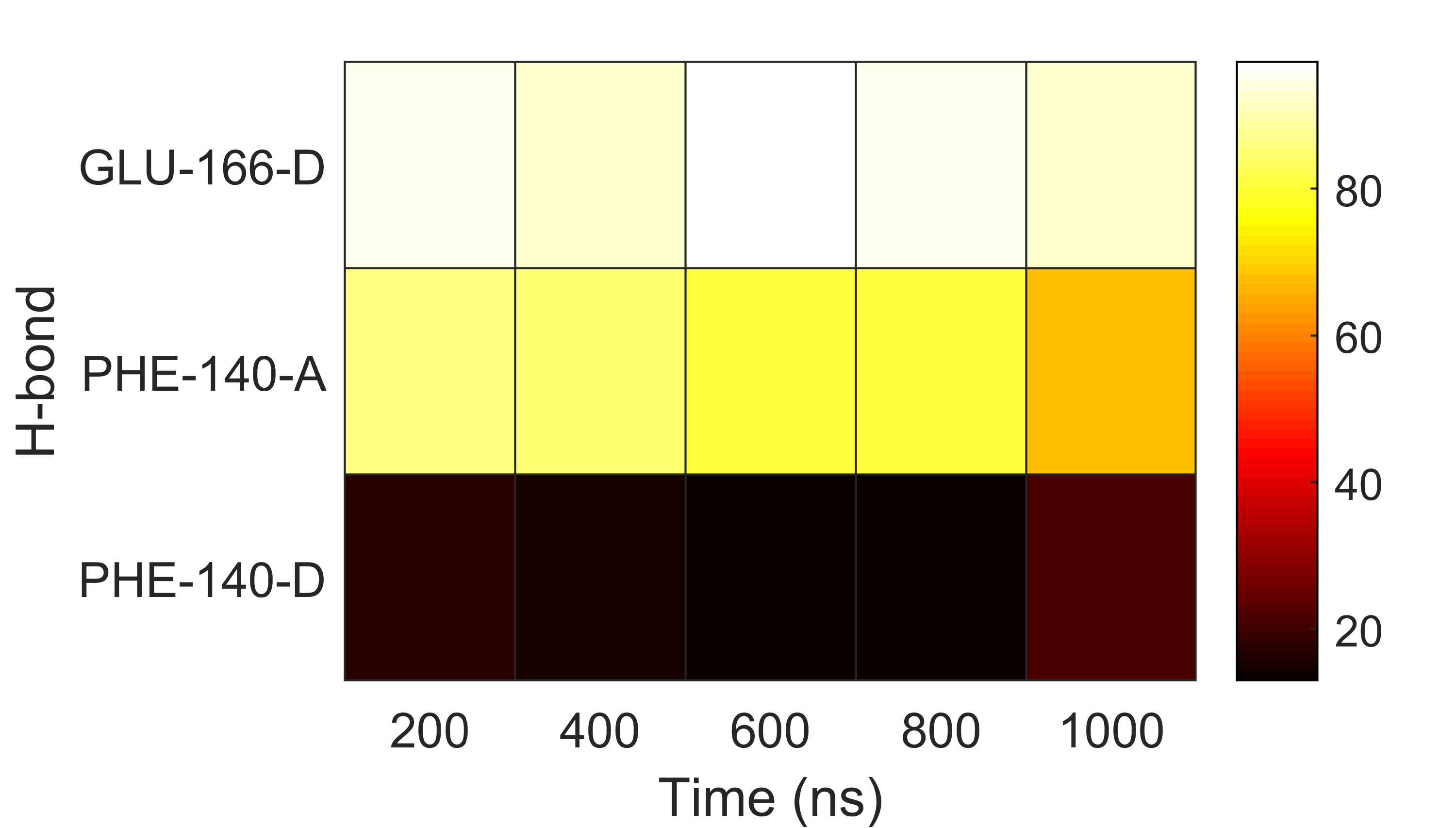** | **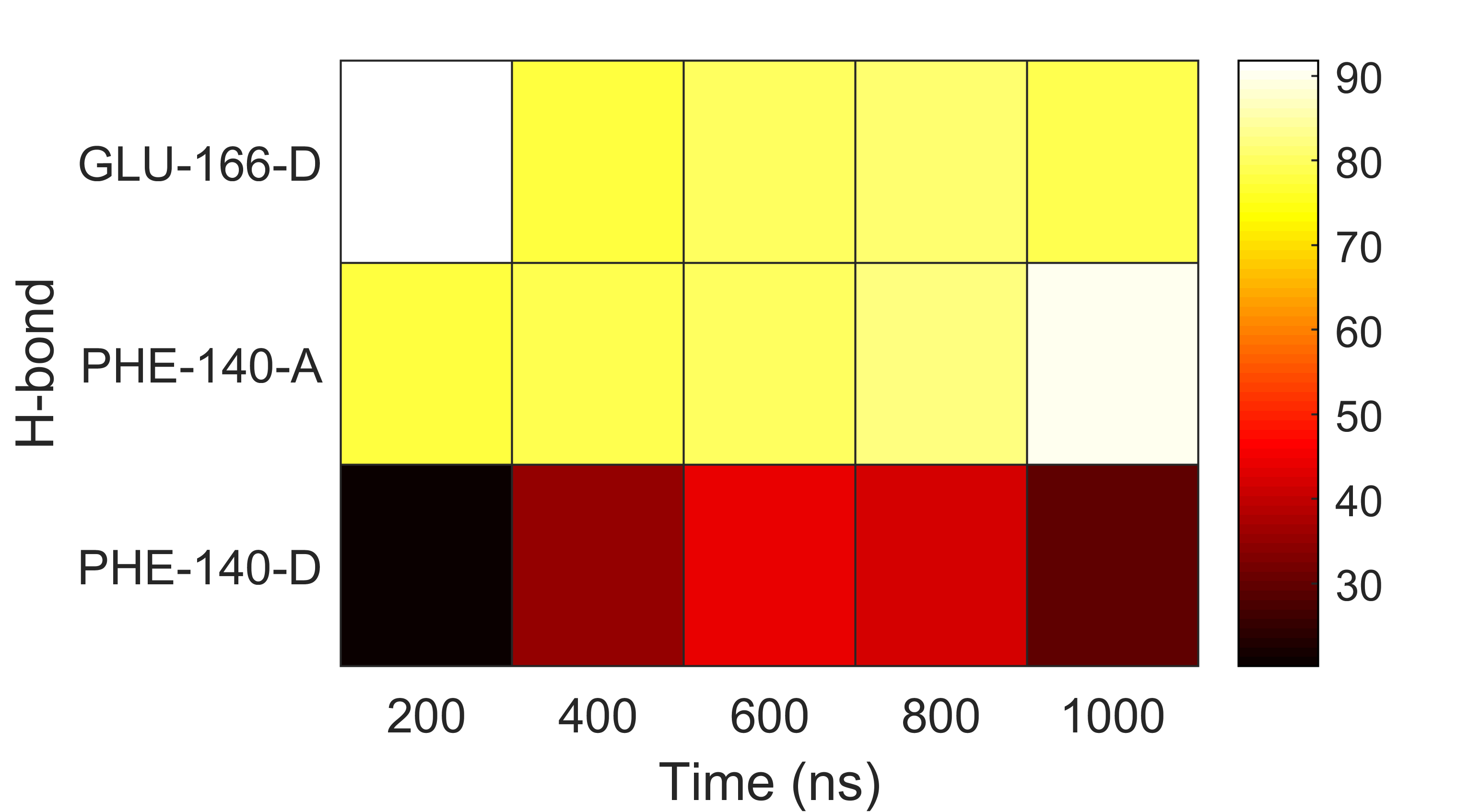** |
| --- | --- |
| **Ser-1 protomer A** | **Ser-1 protomer B** |
| **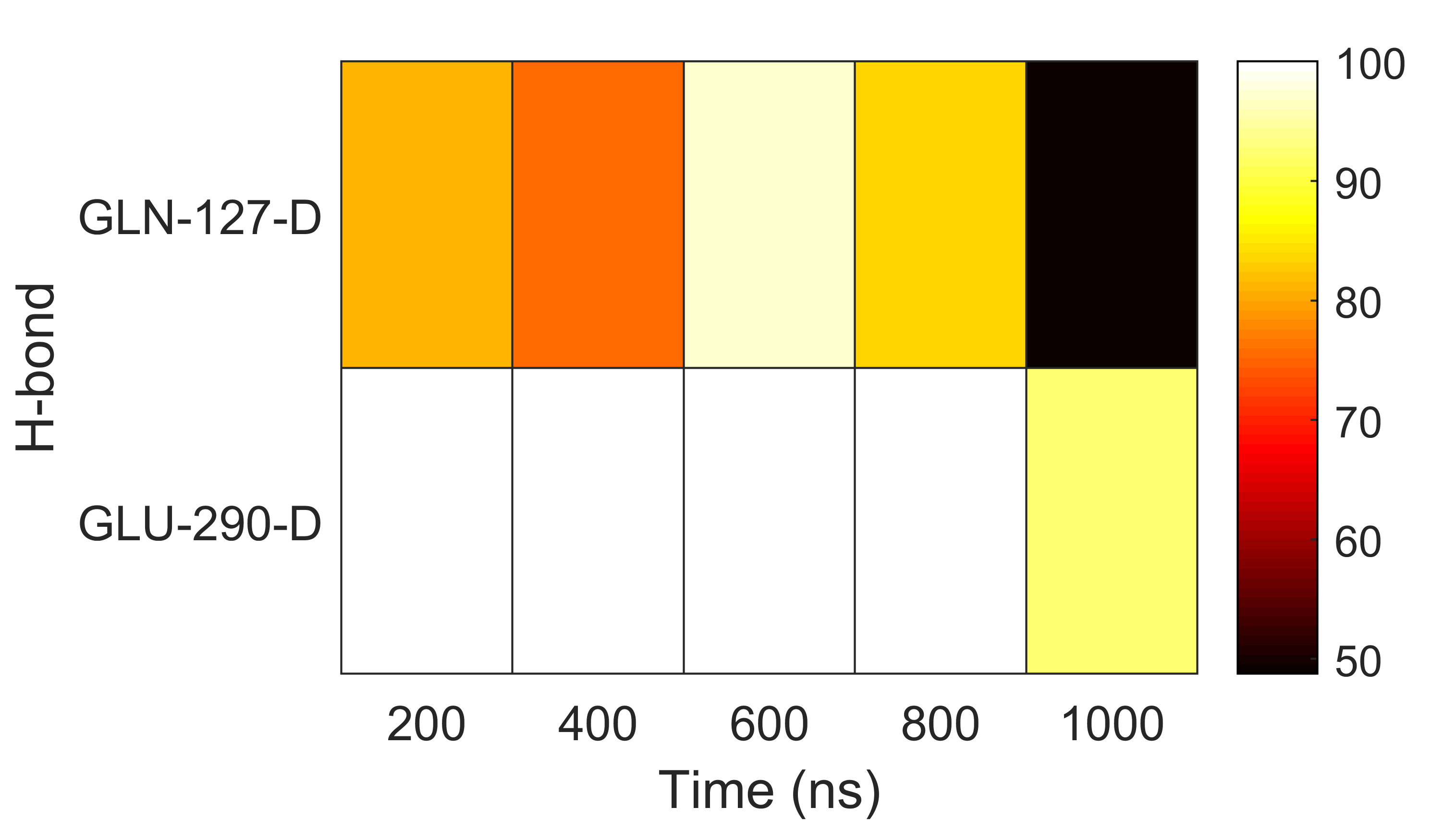** | **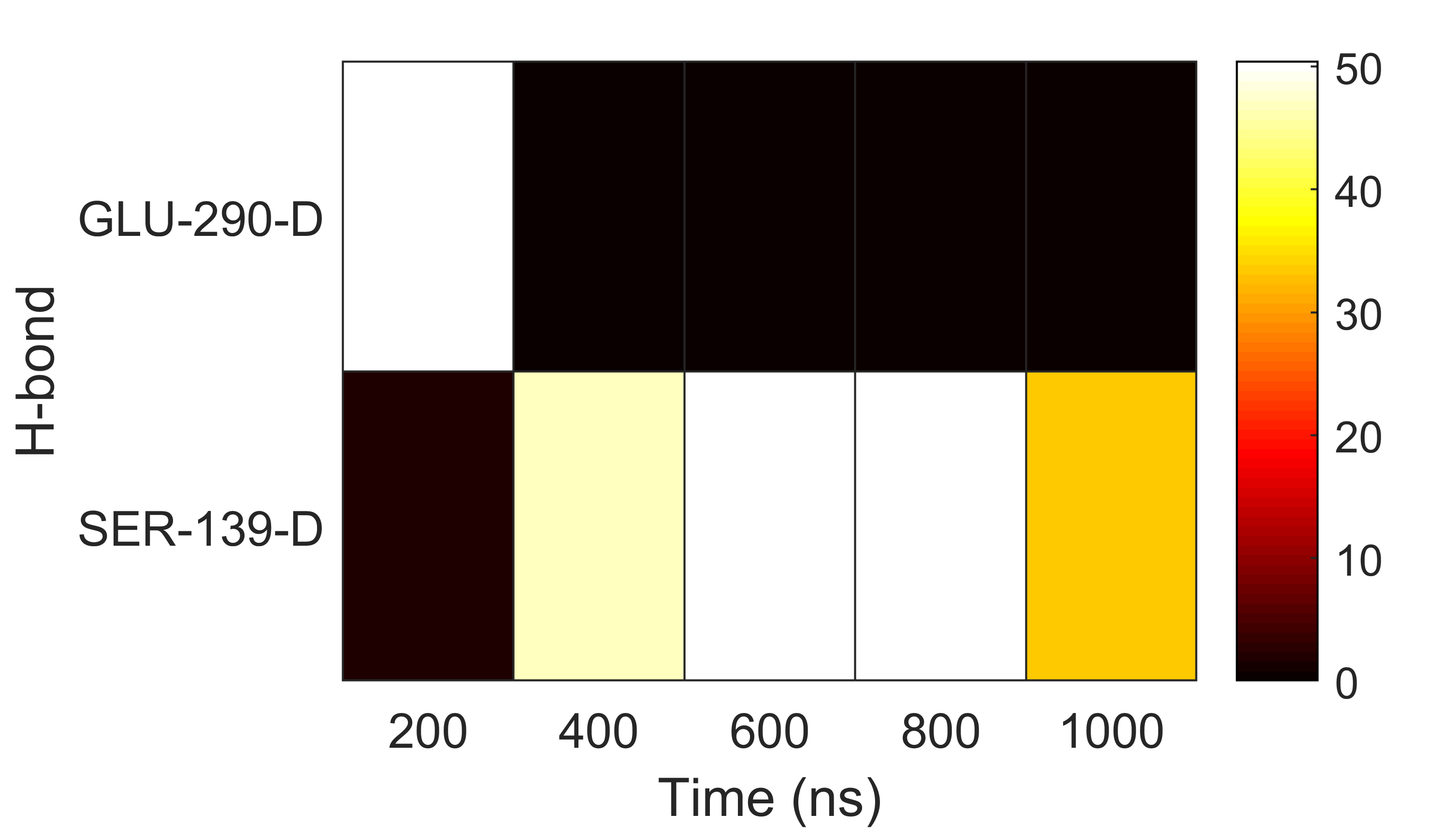** |
| **Arg-4 protomer A** | **Arg-4 protomer B** |

**Figure S18. Occupancy percentages of N-finger hydrogen bonds donors (D) and acceptors (A) for 6LU7 MD simulation.**

| **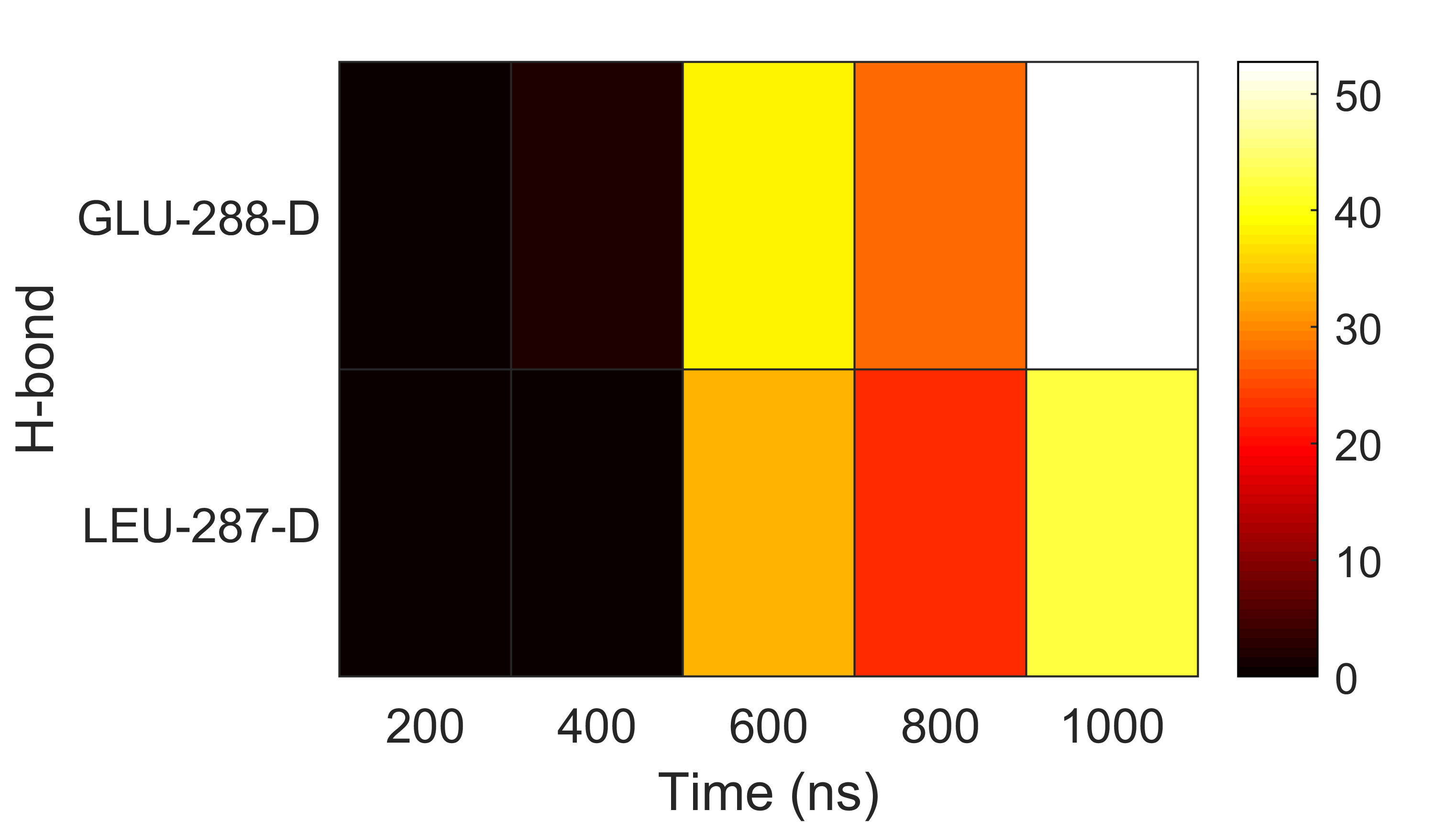** | **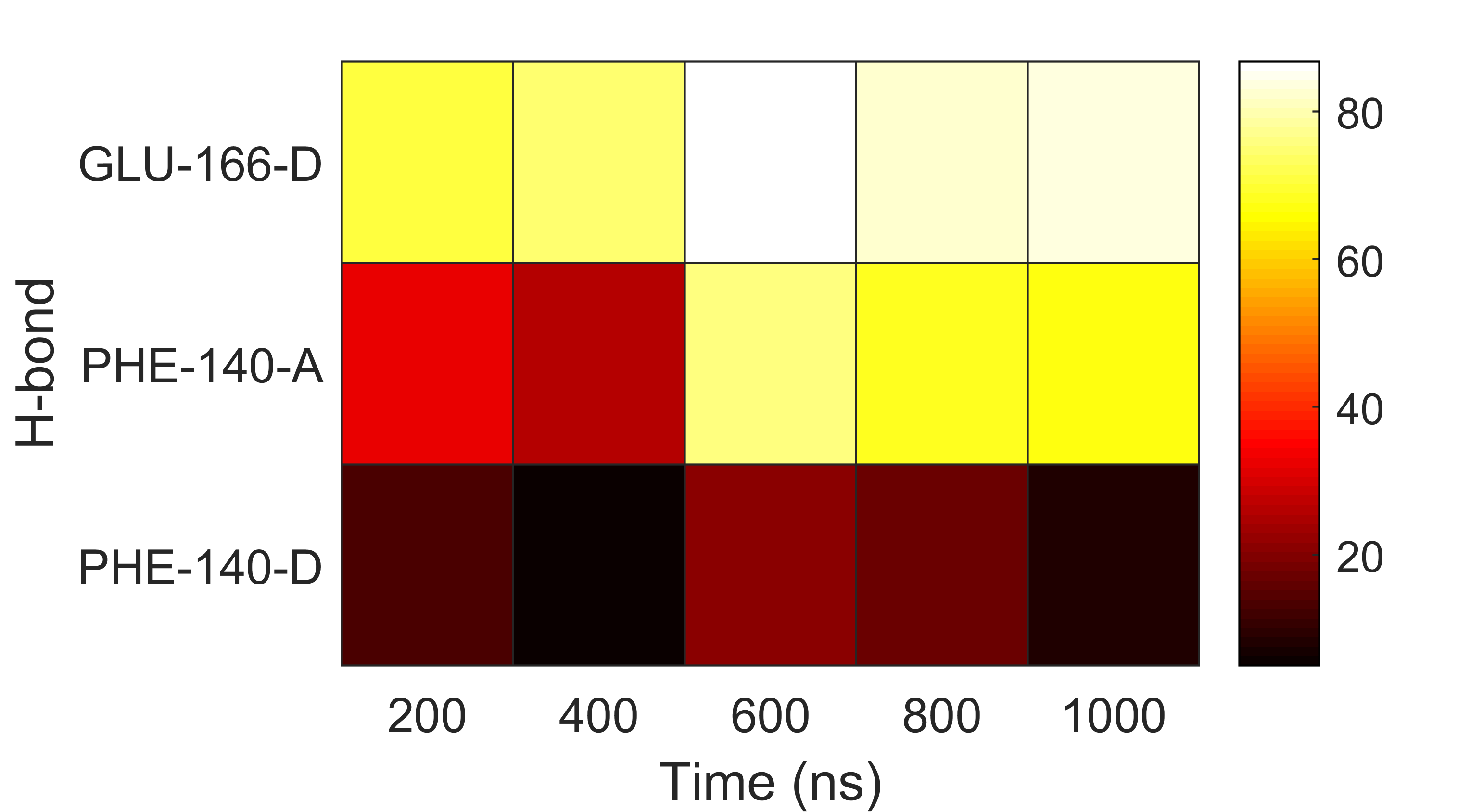** |
| --- | --- |
| **Ser-1 protomer A** | **Ser-1 protomer B** |
| **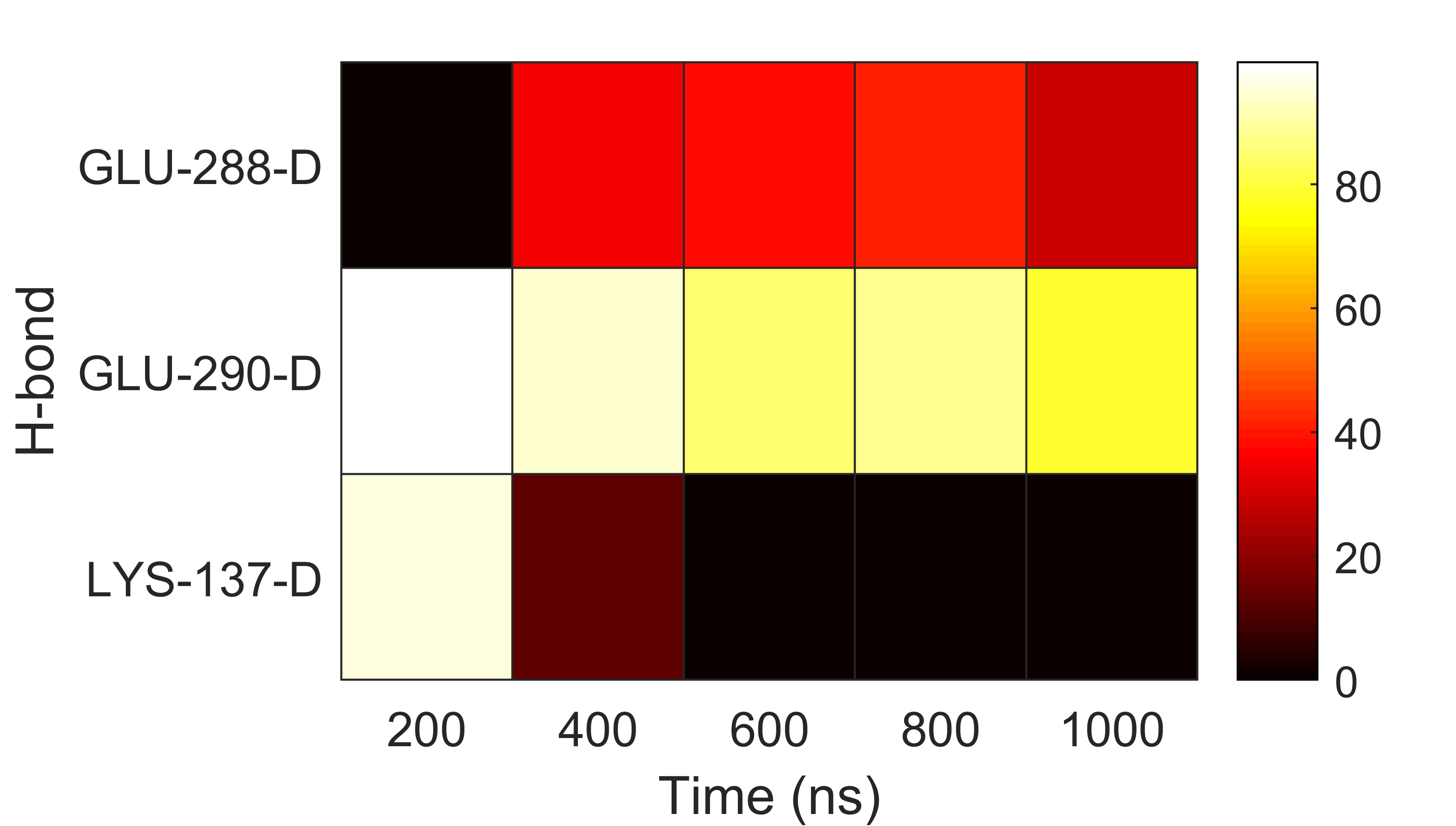** | **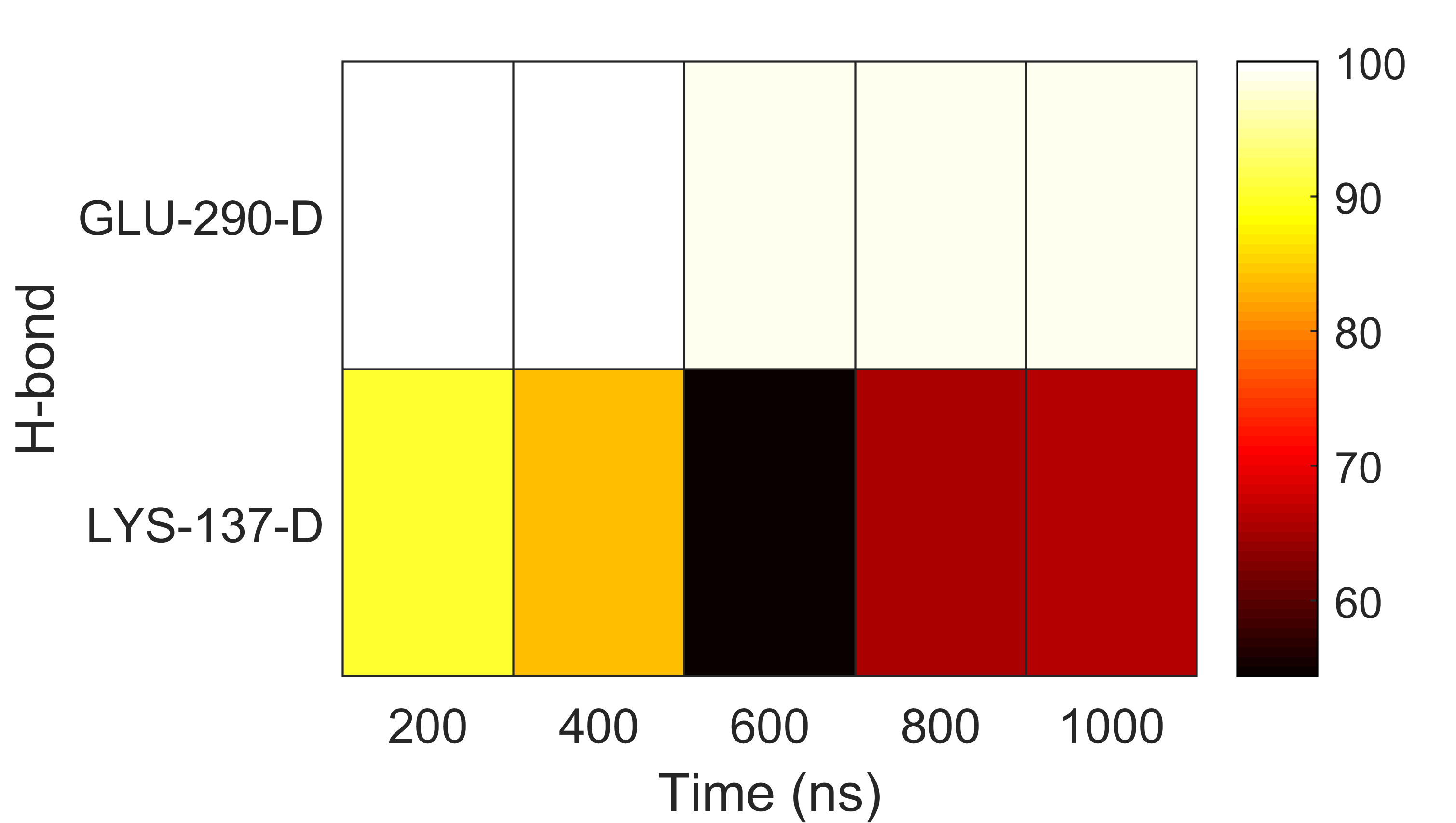** |
| **Arg-4 protomer A** | **Arg-4 protomer B** |

**Figure S19. Occupancy percentages of N-finger hydrogen bonds donors (D) and acceptors (A) for 6Y2F MD simulation.**

| **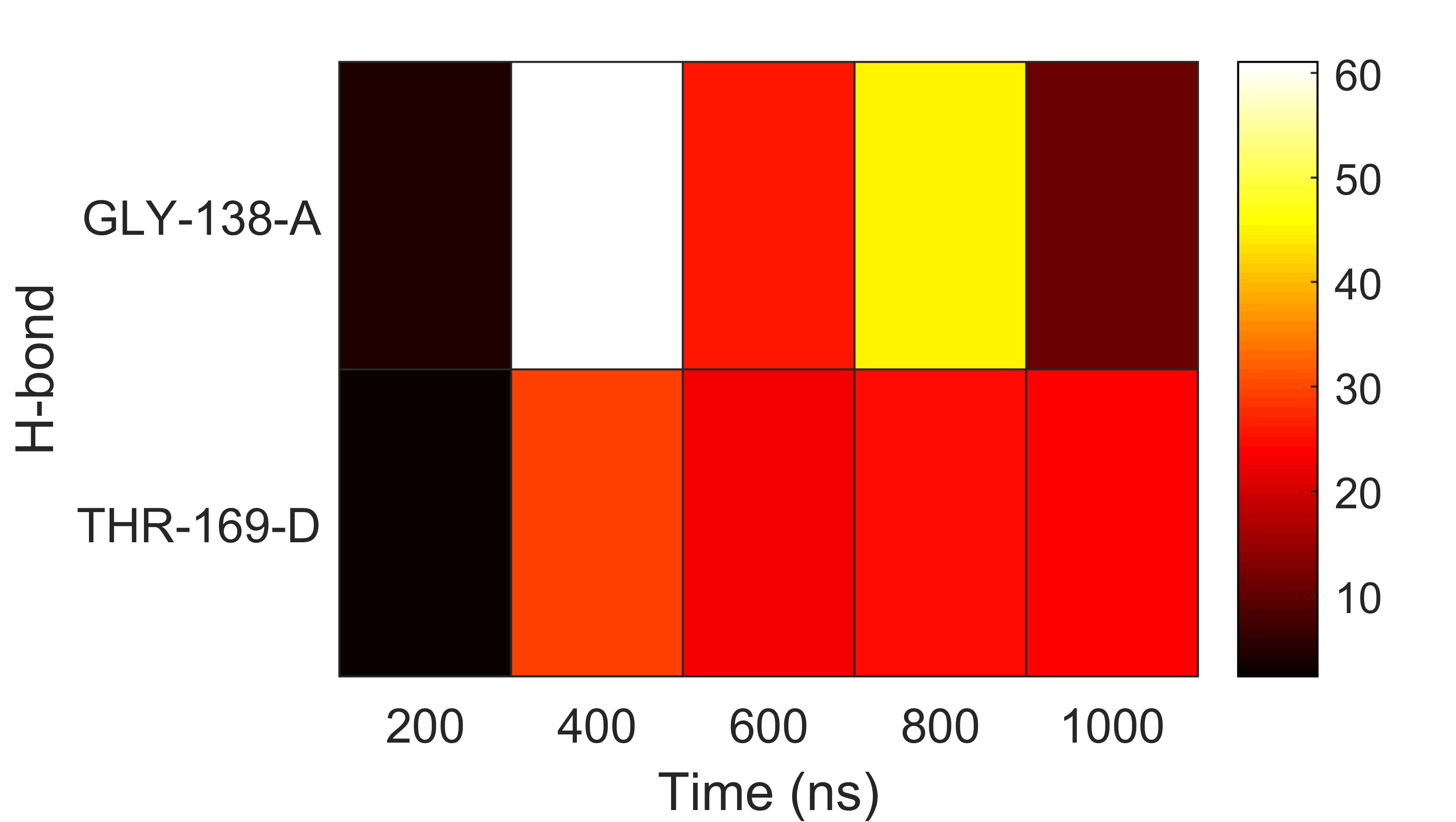** | **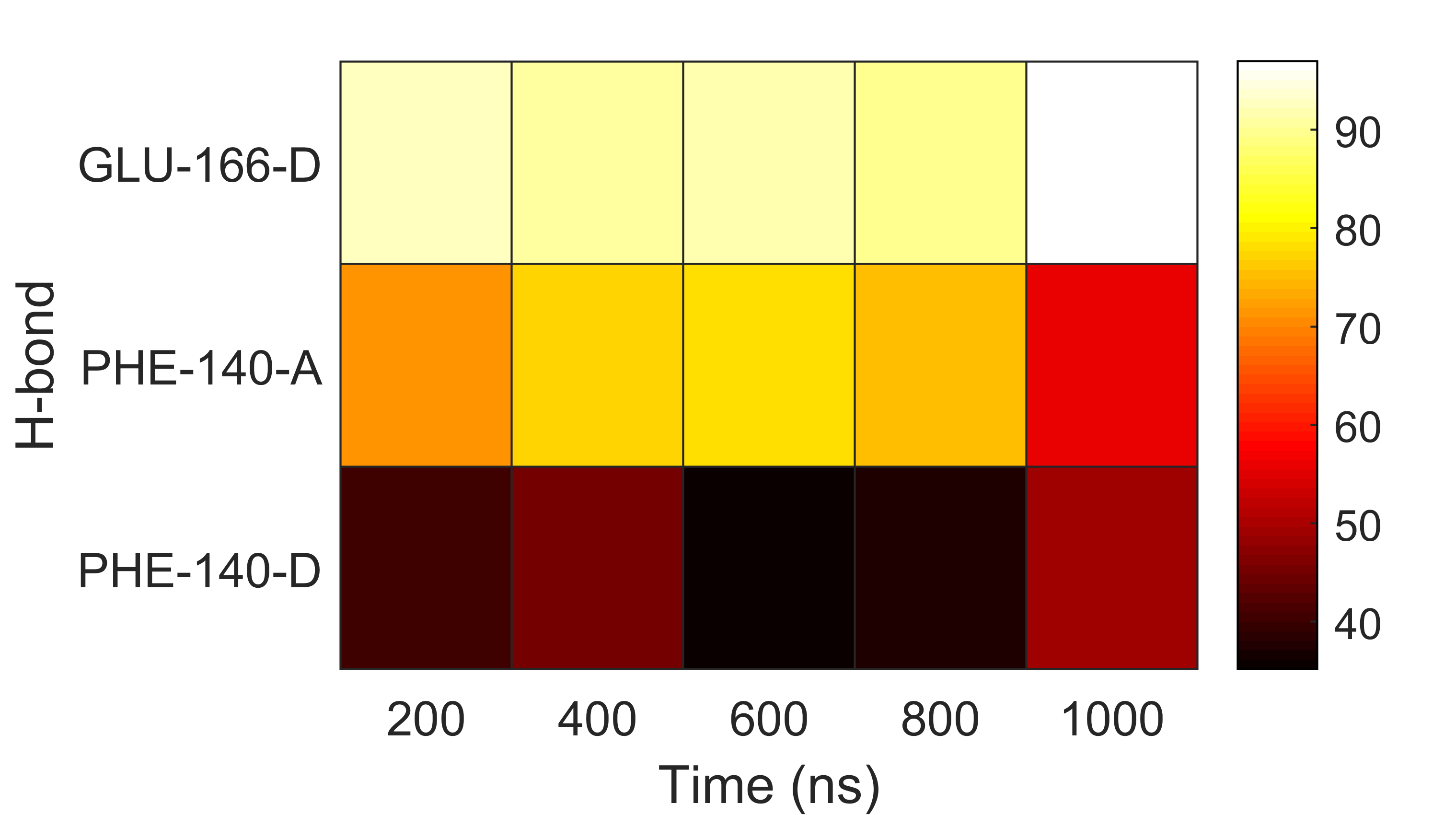** |
| --- | --- |
| **Ser-1 protomer A** | **Ser-1 protomer B** |
| 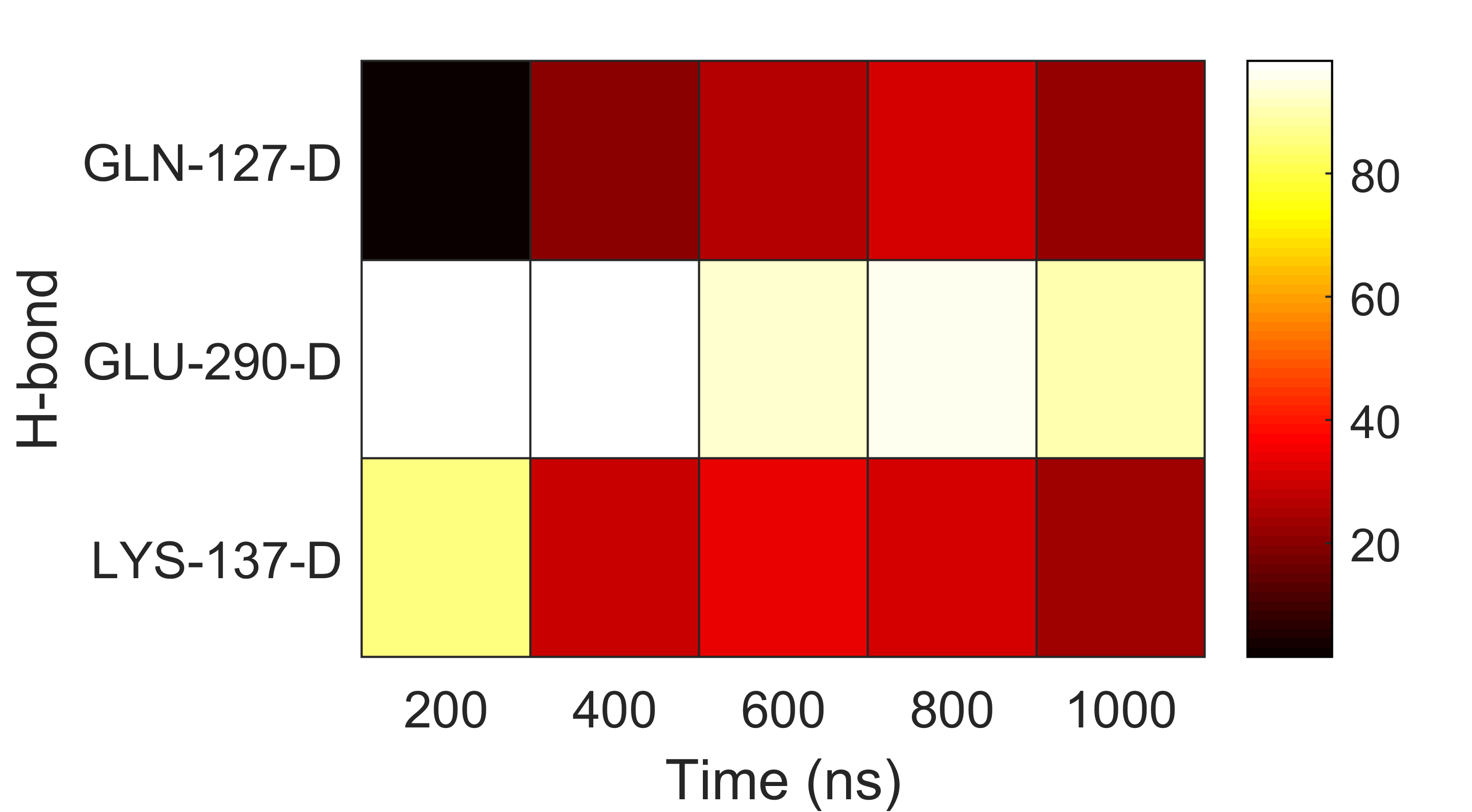 | **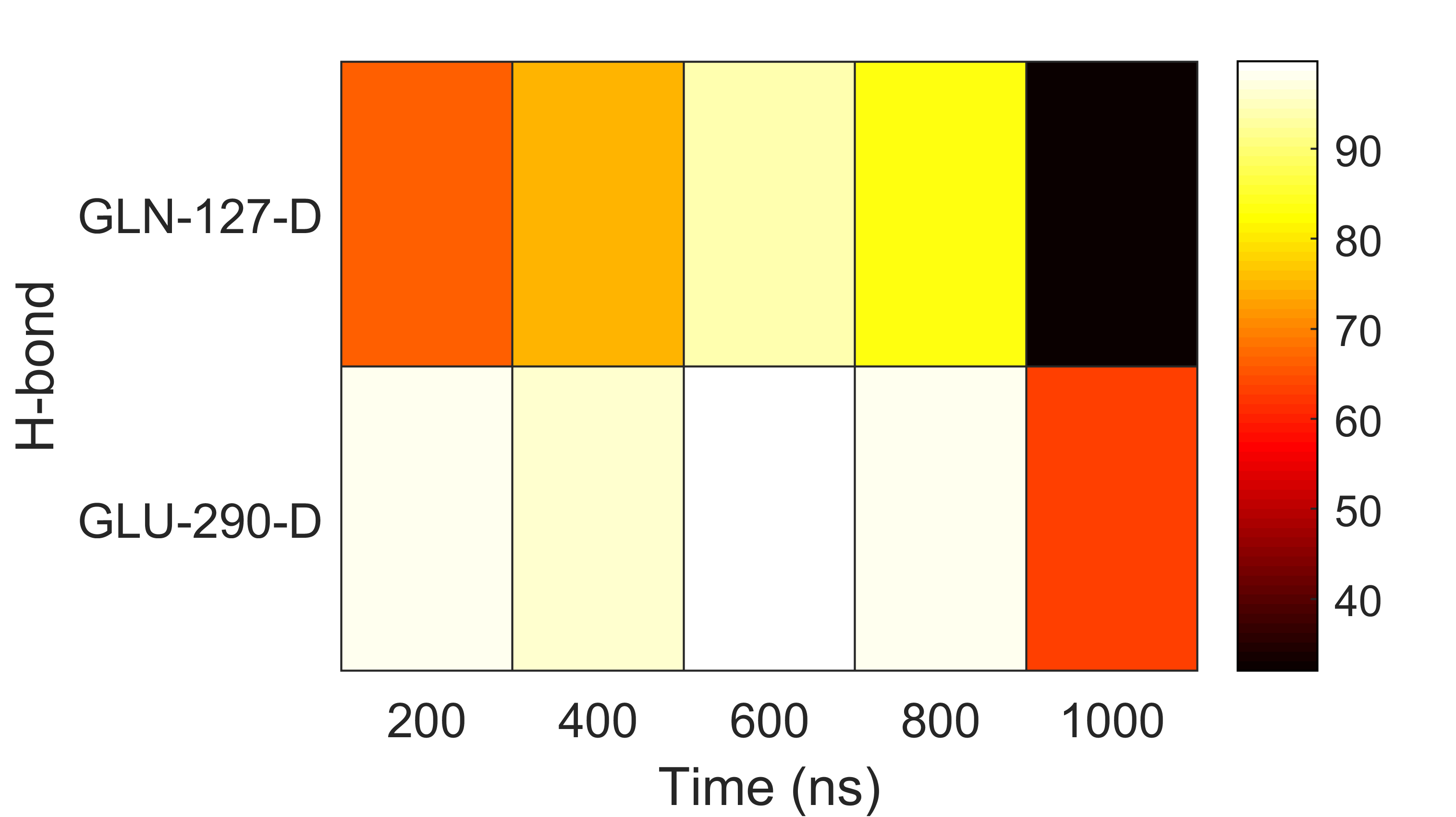** |
| **Arg-4 protomer A** | **Arg-4 protomer B** |

**Figure S20. Occupancy percentages of N-finger hydrogen bonds donors (D) and acceptors (A) for 7K6D MD simulation.**

| **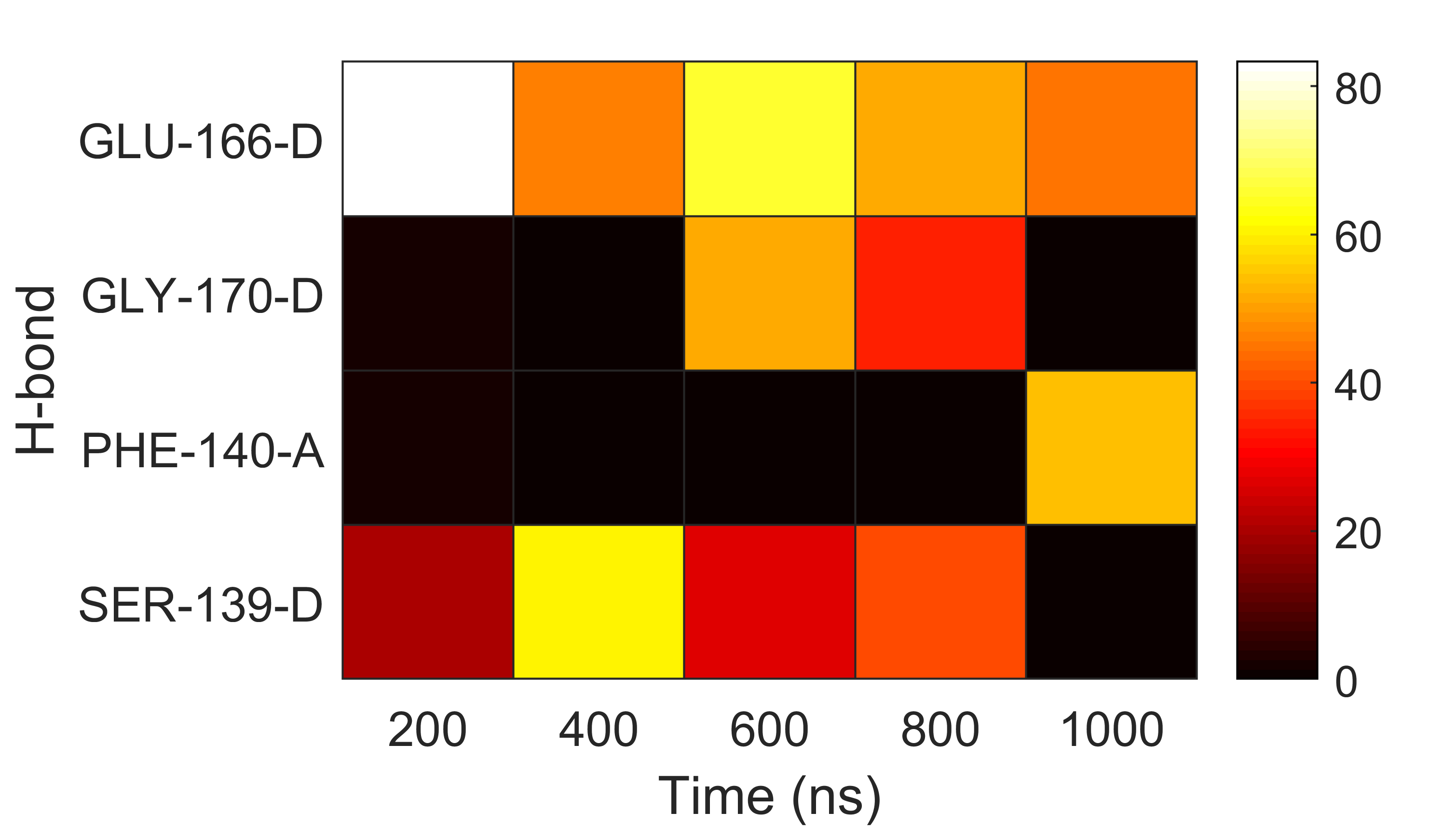** | **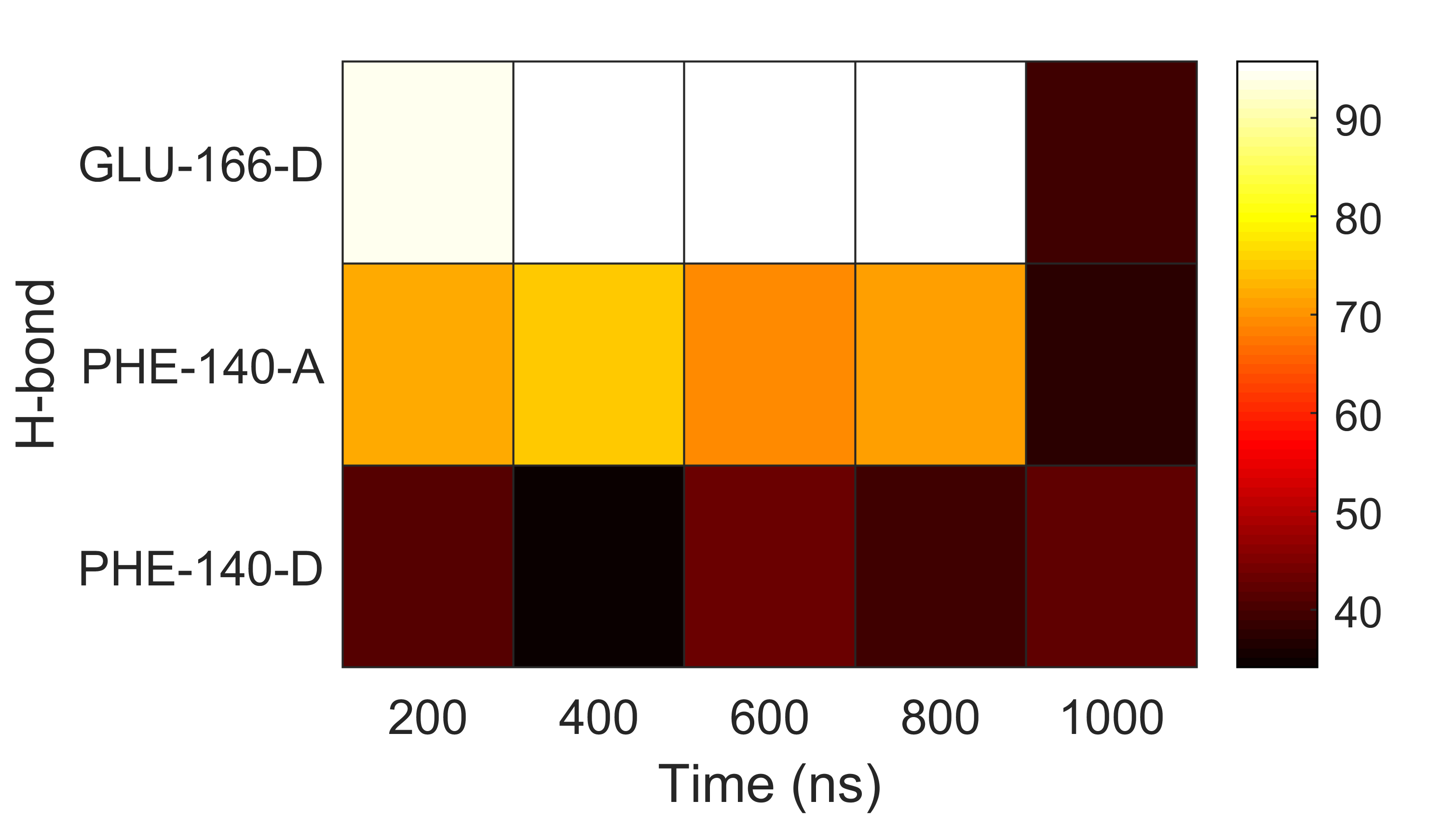** |
| --- | --- |
| **Ser-1 protomer A** | **Ser-1 protomer B** |
| **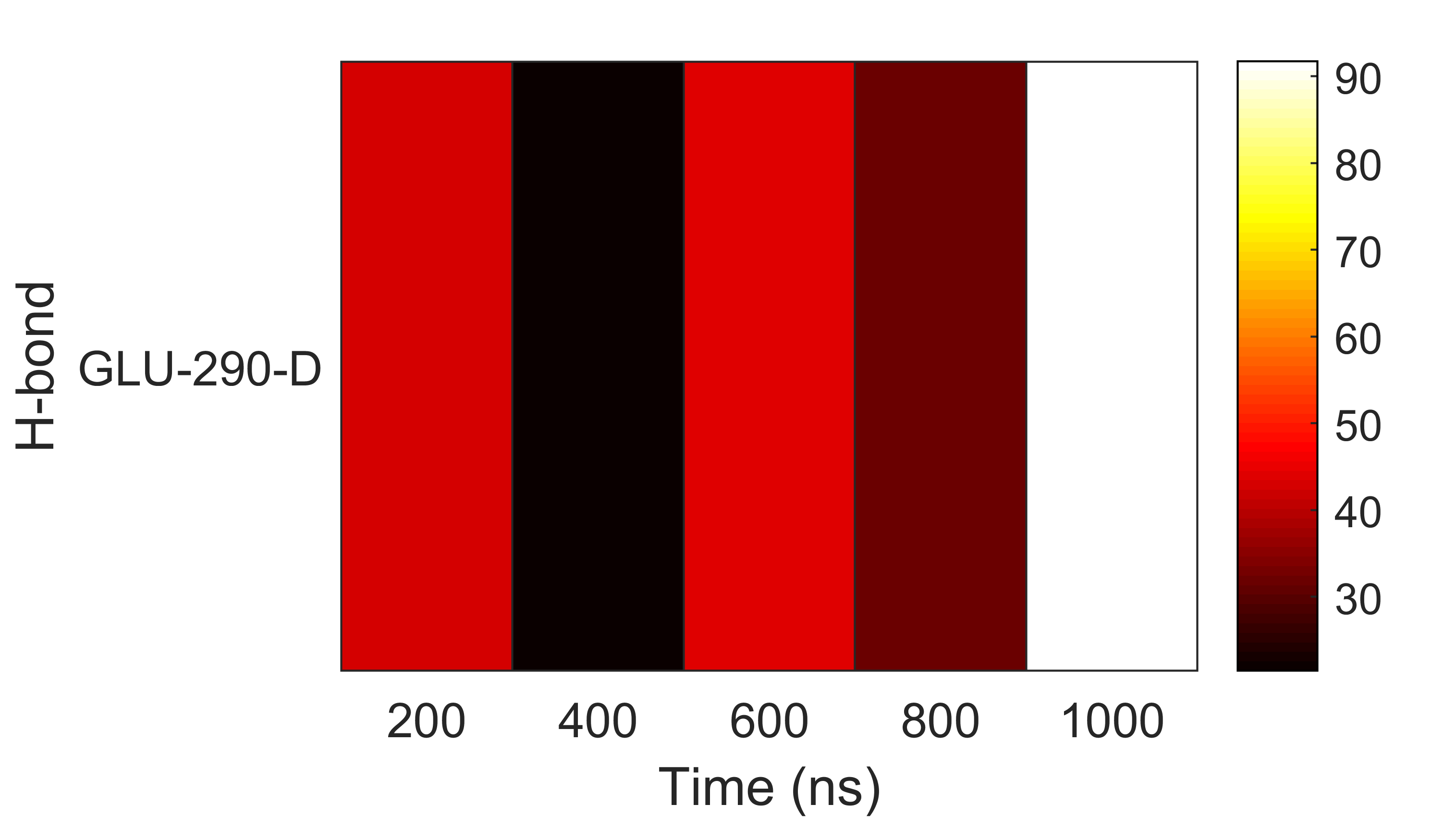** | **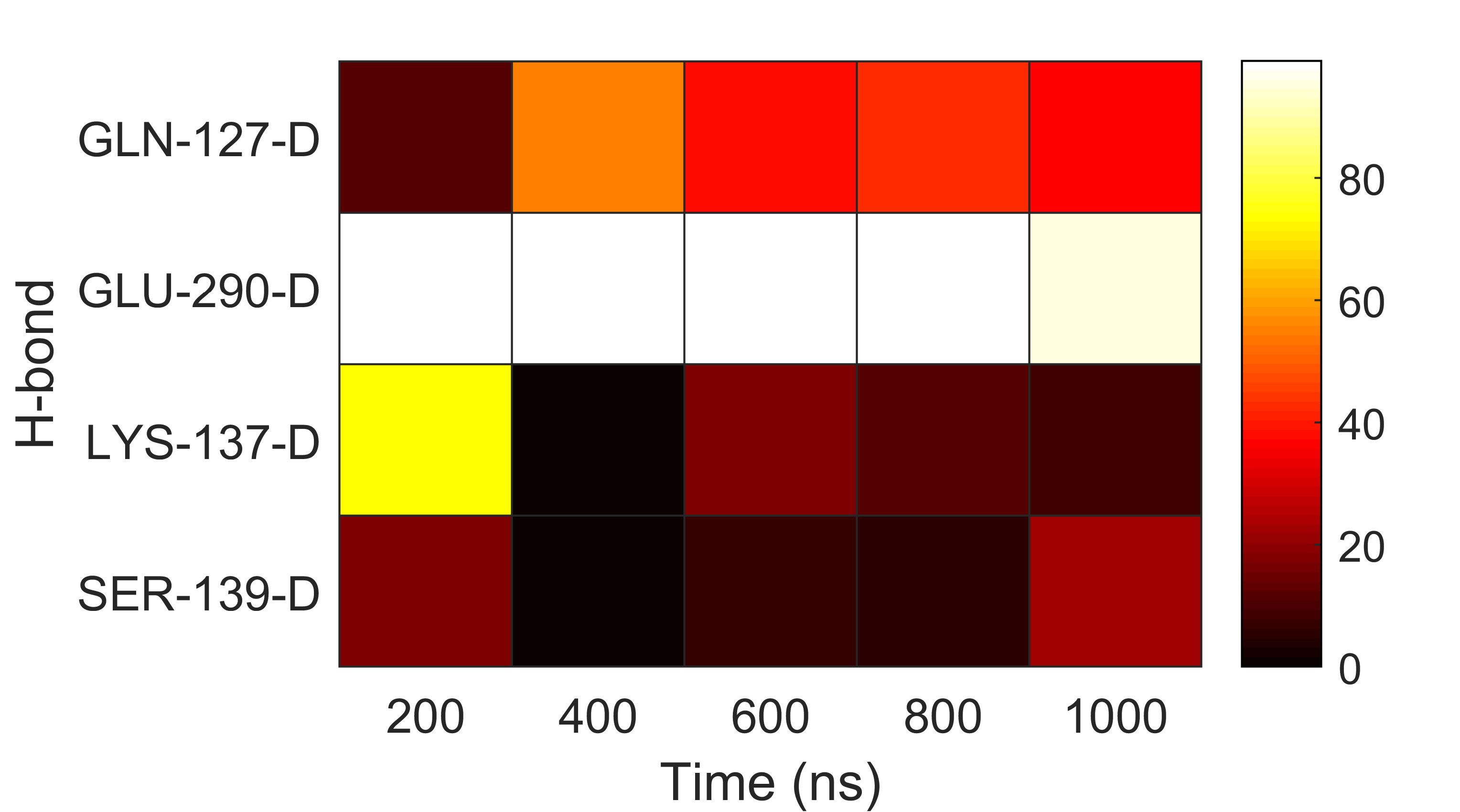** |
| **Arg-4 protomer A** | **Arg-4 protomer B** |

**Figure S21. Occupancy percentages of N-finger hydrogen bonds donors (D) and acceptors (A) for 0026 MD simulation.**

| **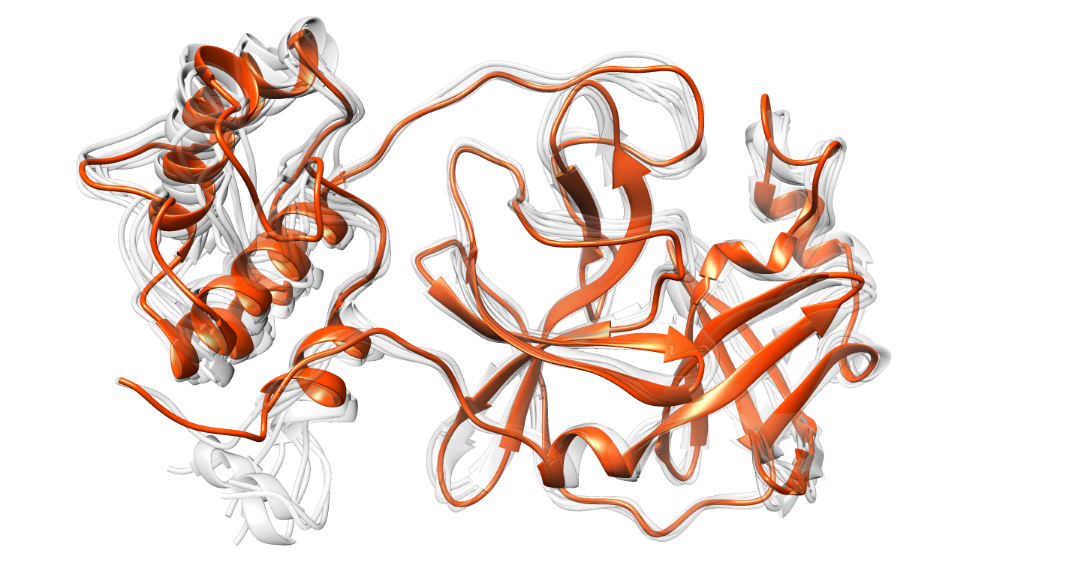** |
| --- |
| **6LU7** |
| **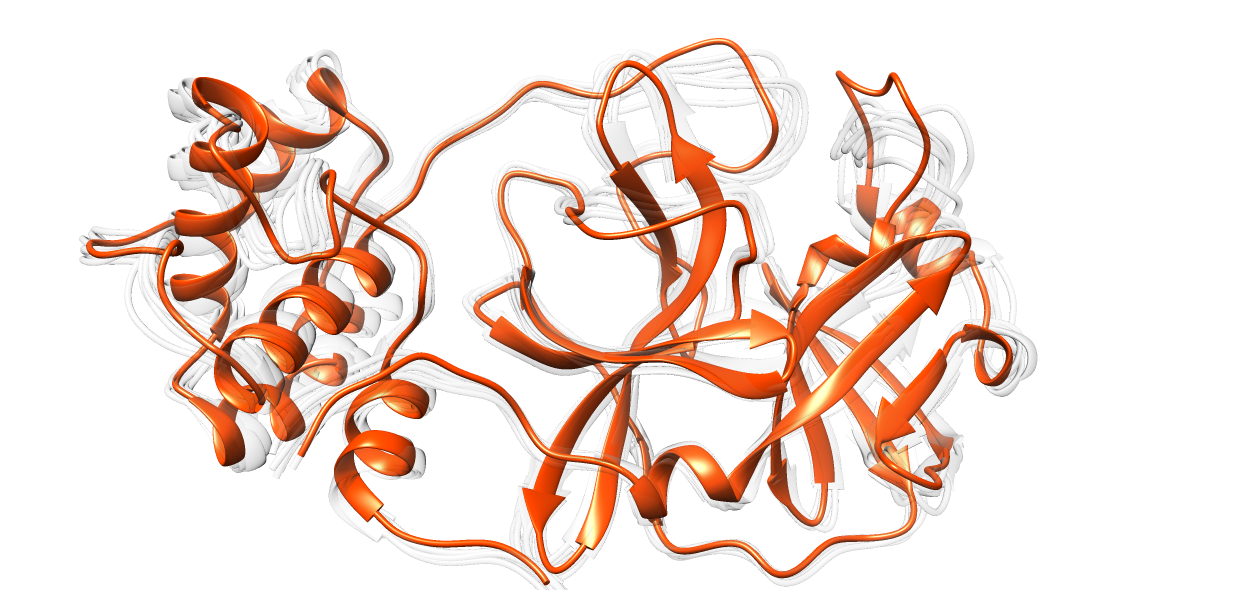** |
| **6Y2F** |
| **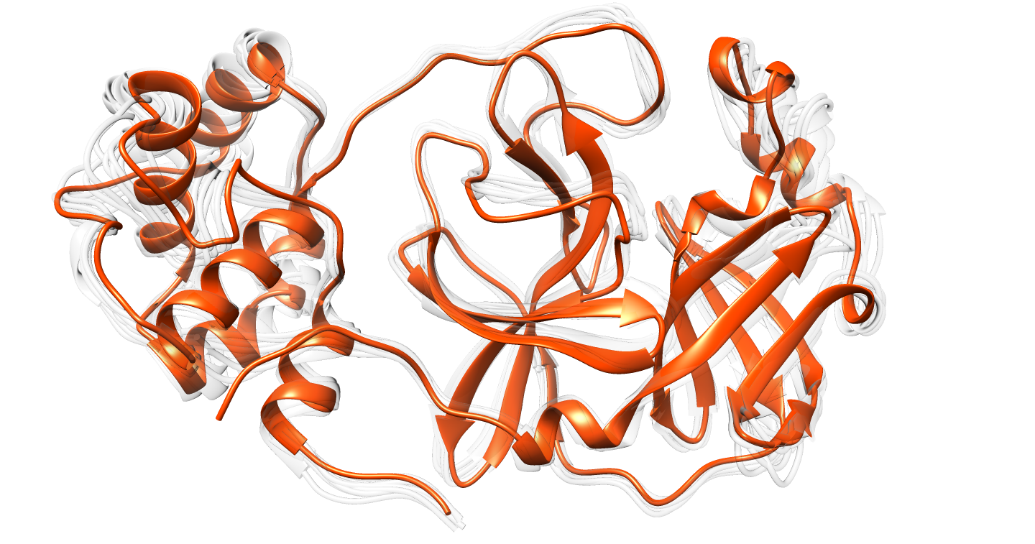** |
| **7K6D** |

**Figure S22. Comparison of 6LU7, 6Y2F and 7K6D crystal structures with several MD frames for one protomer.**

| 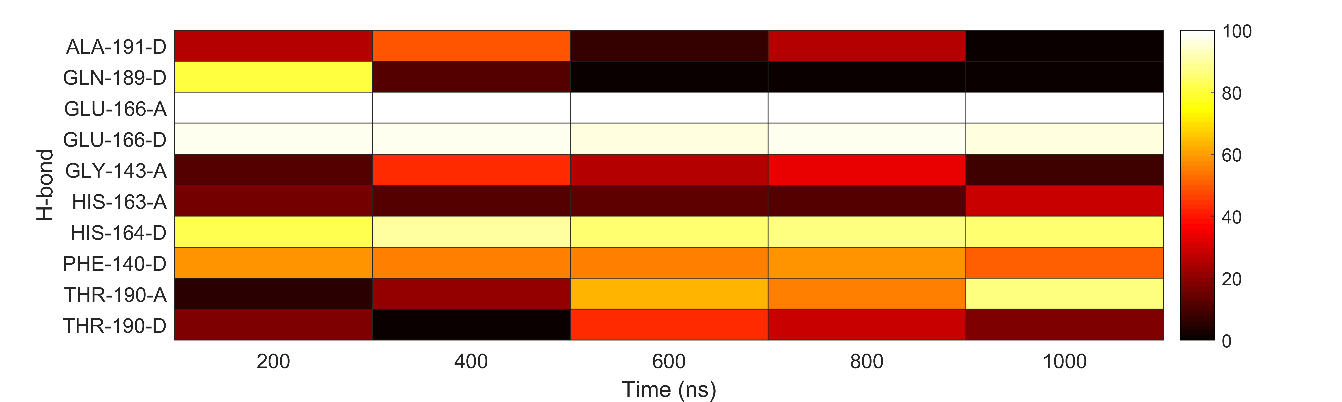  **(A)** |
| --- |
| 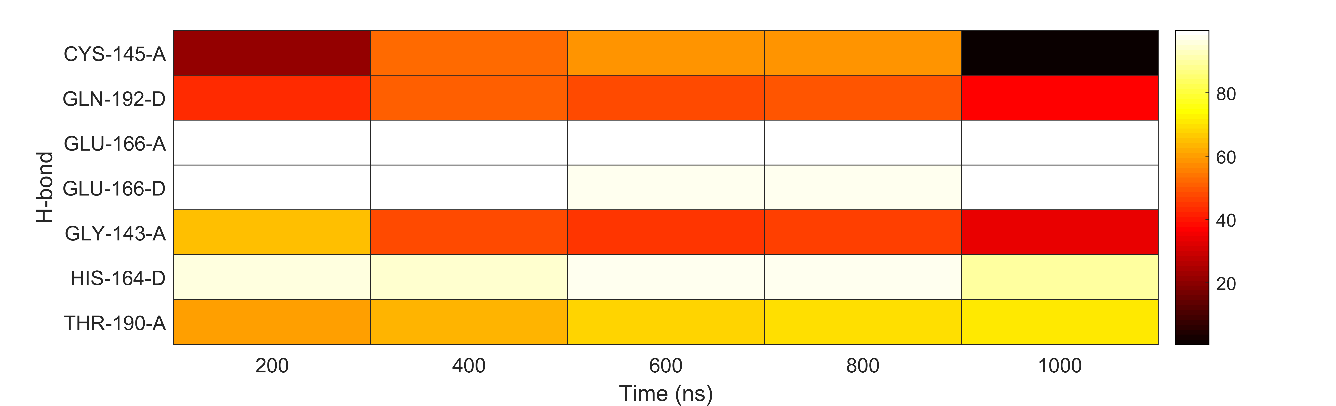  **(B)** |
| 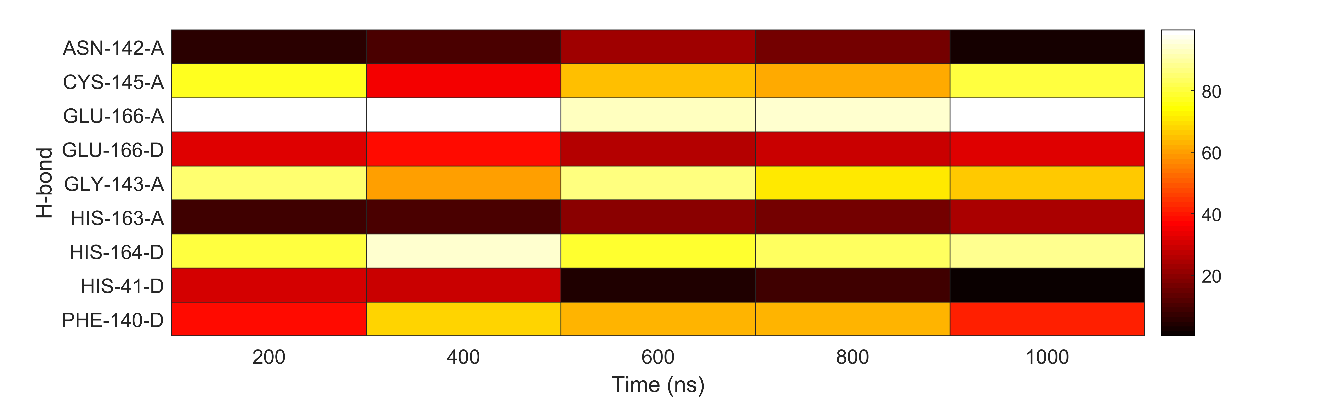  **(C)** |
| 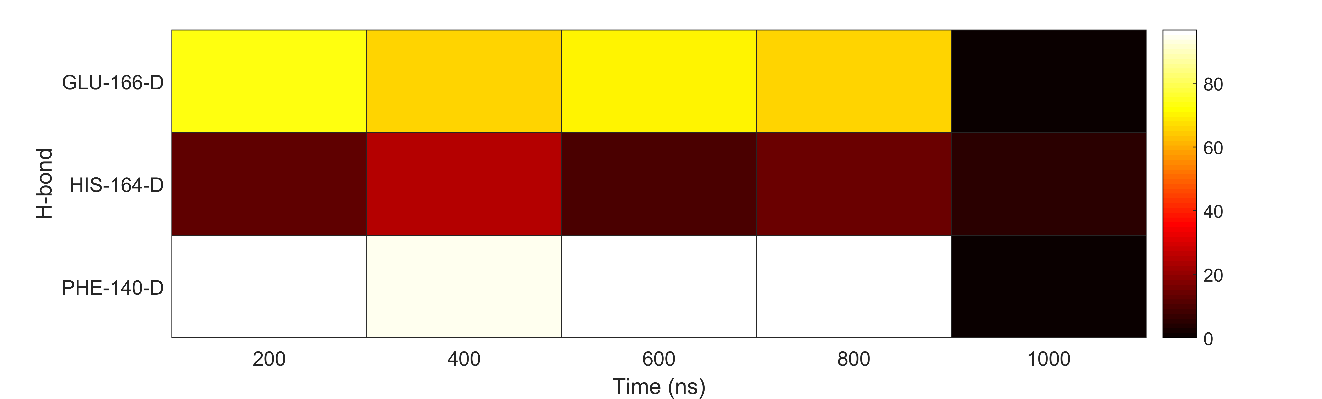  **(D)** |

**Figure S23. Occupancy percentages of protein-ligand hydrogen bonds donors (D) and acceptors (A) for complexes (A) 6LU7, (B) 7K6D, (C) 6Y2F and (D) 0026.**


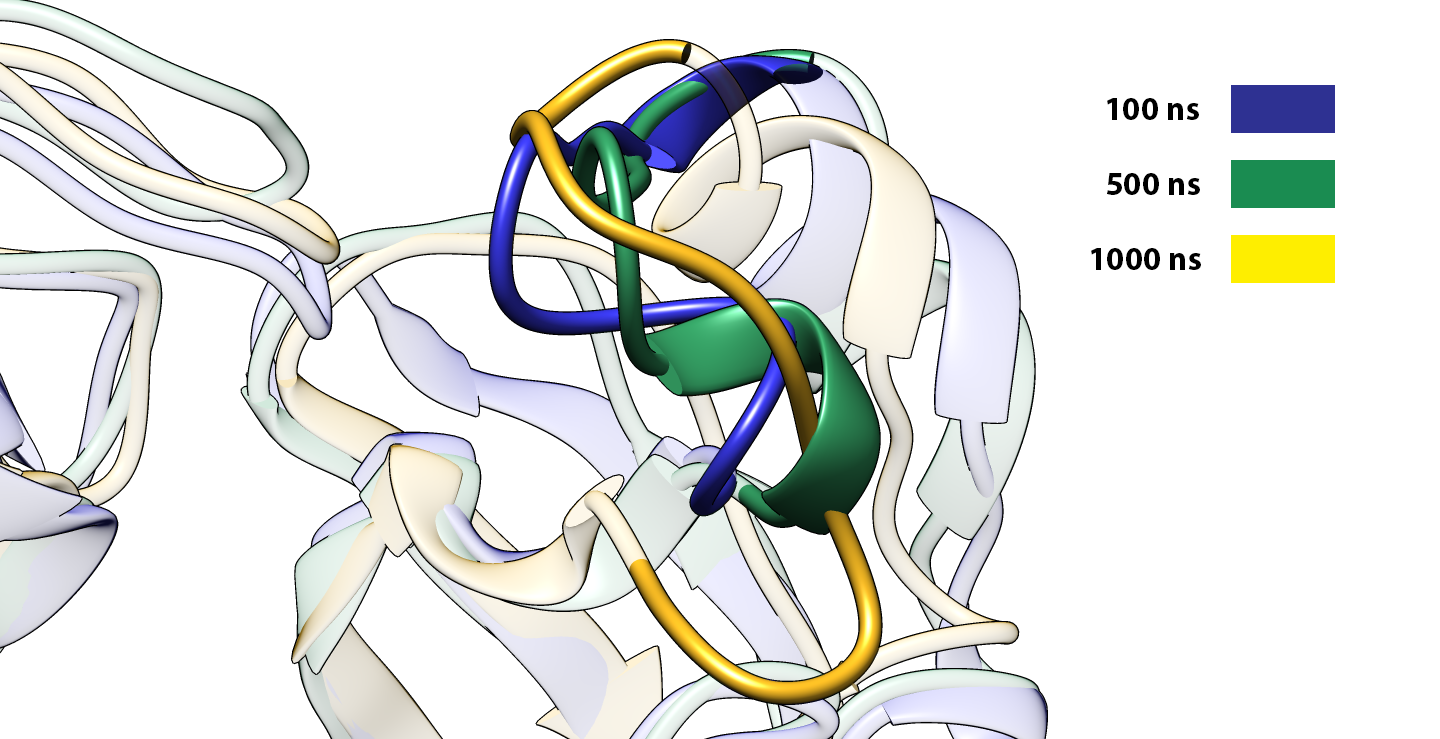


**Figure S24. Movement of residues 45-55 corresponding to simulation times of 100, 500 and 1000 ns for the 7JU7 complex.**

| **Table S8. ΔG MM/GBSA and XP Gscore of the best poses of flexible molecular docking inhibitors from Table S2.**   \| **Ligand** \| **ΔG MM/GBSA (kJ/mol)** \| **XP Gscore (kcal/mol)** \| \| --- \| --- \| --- \| \| **0001** \| ‑71.22 \| ‑4.44 \| \| **0002** \| ‑82.86 \| ‑7.98 \| \| **0003** \| ‑96.17 \| ‑9.07 \| \| **0004** \| ‑100.77 \| ‑8.26 \| \| **0005** \| ‑85.56 \| ‑7.94 \| \| **0006** \| ‑73.68 \| ‑8.55 \| \| **0007** \| ‑103.63 \| ‑12.08 \| \| **0008** \| ‑57.10 \| ‑7.01 \| \| **0009** \| ‑77.70 \| ‑8.66 \| \| **0010** \| ‑84.12 \| ‑7.05 \| \| **0011** \| ‑86.20 \| ‑7.36 \| \| **0012** \| ‑96.01 \| ‑11.60 \| \| **0013** \| ‑86.86 \| ‑8.50 \| \| **0014** \| ‑72.31 \| ‑6.55 \| \| **0015** \| ‑65.66 \| ‑9.07 \| \| **0016** \| ‑88.54 \| ‑11.48 \| | **Table S9. ΔG MM/GBSA and XP Gscore of the best poses of flexible molecular docking inhibitors from Table S3.**   \| **Ligand** \| **ΔG MM/GBSA (kJ/mol)** \| **XP Gscore (kcal/mol)** \| \| --- \| --- \| --- \| \| **0017** \| ‑100.66 \| ‑8.26 \| \| **0018** \| ‑75.03 \| ‑4.94 \| \| **0019** \| ‑91.78 \| ‑9.30 \| \| **0020** \| ‑96.64 \| ‑7.22 \| \| **0021** \| ‑91.70 \| ‑9.97 \| \| **0022** \| ‑48.66 \| ‑4.18 \| \| **0023** \| ‑101.08 \| ‑7.77 \| \| **0024** \| ‑66.24 \| ‑7.01 \| \| **0025** \| ‑61.38 \| ‑5.86 \| \| **0026** \| ‑93.77 \| ‑8.25 \| \| **0027** \| ‑48.49 \| ‑5.10 \| \| **0028** \| ‑67.57 \| ‑5.45 \| \| **0029** \| ‑80.87 \| ‑6.38 \| \| **0030** \| ‑87.95 \| ‑7.86 \| \| **0031** \| ‑67.11 \| ‑6.25 \| \| **0032** \| ‑80.89 \| ‑10.13 \| \| **0033** \| ‑67.20 \| ‑7.19 \| \| **0034** \| ‑90.09 \| ‑6.89 \| \| **0035** \| ‑89.16 \| ‑7.91 \| \| **0036** \| ‑87.02 \| ‑6.41 \| \| **0037** \| ‑92.49 \| ‑8.95 \| \| **0038** \| ‑62.10 \| ‑7.64 \| |
| --- | --- | --- | --- | --- | --- | --- | --- | --- | --- | --- | --- | --- | --- | --- | --- | --- | --- | --- | --- | --- | --- | --- | --- | --- | --- | --- | --- | --- | --- | --- | --- | --- | --- | --- | --- | --- | --- | --- | --- | --- | --- | --- | --- | --- | --- | --- | --- | --- | --- | --- | --- | --- | --- | --- | --- | --- | --- | --- | --- | --- | --- | --- | --- | --- | --- | --- | --- | --- | --- | --- | --- | --- | --- | --- | --- | --- | --- | --- | --- | --- | --- | --- | --- | --- | --- | --- | --- | --- | --- | --- | --- | --- | --- | --- | --- | --- | --- | --- | --- | --- | --- | --- | --- | --- | --- | --- | --- | --- | --- | --- | --- | --- | --- | --- | --- | --- | --- | --- | --- | --- | --- |

| **Table S10. ΔG MM/PBSA for inhibitors from Table S1 with 500 ns MD production. *Ligand left the active site during MD simulation.**   \| **Ligand** \| **ΔG MM/PBSA Protomer A (kJ/mol)** \| **ΔG MM/PBSA Protomer B (kJ/mol)** \| \| --- \| --- \| --- \| \| **6LU7** \| ‑173.276 ± 19.172 \| ‑186.199 ± 17.12 \| \| **6LZE** \| ‑97.429 ± 16.743 \| ‑117.557 ± 14.829 \| \| **6M0K** \| ‑99.146 ± 16.901 \| ‑110.293 ± 14.926 \| \| **6M2N** \| ‑47.370 ± 11.310 \| * \| \| **6W63** \| ‑126.988 ± 13.821 \| ‑122.64 ± 12.931 \| \| **6WTK** \| ‑93.694 ± 26.402 \| ‑123.667 ± 16.190 \| \| **6XMK** \| ‑135.979 ± 14.544 \| ‑121.119 ± 17.797 \| \| **6XR3** \| ‑116.419 ± 16.658 \| ‑159.382 ± 17.410 \| \| **6Y2F** \| ‑152.062 ± 20.176 \| ‑149.913 ± 16.724 \| \| **7C8R** \| ‑131.757 ± 20.071 \| ‑175.211 ± 22.528 \| \| **7C8T** \| ‑179.599 ± 15.006 \| ‑164.866 ± 20.509 \| \| **7JU7** \| ‑283.911 ± 33.858 \| ‑236.876 ± 20.951 \| \| **7JYC** \| ‑136.69 ± 26.327 \| ‑123.062 ± 16.566 \| \| **7K40** \| ‑128.047 ± 18.914 \| ‑83.409 ± 18.888 \| \| **7K6D** \| ‑233.124 ± 21.096 \| ‑157.725 ± 22.151 \| | **Table S11. ΔG MM/PBSA for inhibitors from Table S2 with 500 ns MD production. *Ligand left the active site during MD simulation.**   \| **Ligand** \| **ΔG MM/PBSA Protomer A (kJ/mol)** \| **ΔG MM/PBSA Protomer B (kJ/mol)** \| \| --- \| --- \| --- \| \| **0001** \| 1.685 ± 27.160 \| 22.424 ± 26.177 \| \| **0002** \| ‑109.753 ± 18.279 \| ‑115.632 ± 15.324 \| \| **0003** \| ‑103.912 ± 19.587 \| ‑146.032 ± 19.626 \| \| **0004** \| ‑156.915 ± 15.031 \| ‑158.694 ± 14.926 \| \| **0005** \| ‑107.458 ± 23.132 \| ‑90.057 ± 19.664 \| \| **0006** \| ‑96.925 ± 21.596 \| ‑121.353 ± 18.427 \| \| **0007** \| ‑99.988 ± 19.642 \| ‑164.767 ± 16.788 \| \| **0008** \| * \| ‑57.445 ± 13.056 \| \| **0009** \| ‑108.126 ± 21.081 \| ‑111.303 ± 19.747 \| \| **0010** \| ‑238.365 ± 21.342 \| ‑248.230 ± 19.860 \| \| **0011** \| ‑121.76 ± 19.671 \| ‑113.318 ± 21.196 \| \| **0012** \| ‑241.699 ± 23.550 \| ‑253.039 ± 26.988 \| \| **0013** \| ‑124.291 ± 20.220 \| ‑136.841 ± 19.327 \| \| **0014** \| ‑97.119 ± 21.969 \| ‑102.044 ± 21.927 \| \| **0015** \| ‑45.196 ± 14.005 \| ‑73.806 ± 18.655 \| \| **0016** \| ‑81.154 ± 21.895 \| ‑93.341 ± 25.476 \| |
| --- | --- | --- | --- | --- | --- | --- | --- | --- | --- | --- | --- | --- | --- | --- | --- | --- | --- | --- | --- | --- | --- | --- | --- | --- | --- | --- | --- | --- | --- | --- | --- | --- | --- | --- | --- | --- | --- | --- | --- | --- | --- | --- | --- | --- | --- | --- | --- | --- | --- | --- | --- | --- | --- | --- | --- | --- | --- | --- | --- | --- | --- | --- | --- | --- | --- | --- | --- | --- | --- | --- | --- | --- | --- | --- | --- | --- | --- | --- | --- | --- | --- | --- | --- | --- | --- | --- | --- | --- | --- | --- | --- | --- | --- | --- | --- | --- | --- | --- | --- | --- |

**Table S12. ΔG MM/PBSA for inhibitors from Table S3 with 500 ns MD production. *** **Ligand left the active site during MD simulation.**

| **Ligand** | **ΔG MM/PBSA Protomer A (kJ/mol)** | **ΔG MM/PBSA Protomer B (kJ/mol)** |
| --- | --- | --- |
| **0017** | ‑104 ± 19.327 | ‑101.239 ± 26.477 |
| **0018** | ‑104.762 ± 19.517 | ‑89.039 ± 17.099 |
| **0019** | ‑120.041 ± 26.095 | ‑138.489 ± 25.458 |
| **0020** | ‑131.461 ± 21.373 | ‑163.708 ± 23.625 |
| **0021** | ‑116.613 ± 19.81 | ‑129.601 ± 25.023 |
| **0022** | ‑83.607 ± 18.698 | * |
| **0023** | ‑100.638 ± 19.948 | ‑97.15 ± 18.358 |
| **0024** | * | ‑109.569 ± 16.313 |
| **0025** | ‑82.079 ± 20.78 | ‑88.096 ± 20.299 |
| **0026** | ‑149.412 ± 17.307 | ‑162.825 ± 16.43 |
| **0027** | 54.698 ± 22.629 | * |
| **0028** | 40.7 ± 19.353 | 28.312 ± 19.607 |
| **0029** | ‑86.753 ± 17.09 | ‑95.21 ± 16.293 |
| **0030** | ‑206.388 ± 18.047 | ‑210.406 ± 16.451 |
| **0031** | 62.55 ± 24.932 | 55.49 ± 26.658 |
| **0032** | * | 68.892 ± 24.206 |
| **0033** | * | ‑97.368 ± 14.717 |
| **0034** | ‑139.518 ± 18.489 | ‑142.902 ± 16.641 |
| **0035** | ‑159.685 ± 19.736 | ‑145.666 ± 21.781 |
| **0036** | ‑140.003 ± 16.565 | ‑120.356 ± 20.728 |
| **0037** | ‑107.283 ± 24.423 | * |
| **0038** | ‑112.678 ± 13.293 | ‑89.737 ± 16.967 |

**Table S13. First ΔG MM/PBSA ranking (affinity ranking) for inhibitors.**

| **Position** | **Ligand** | **ΔG MM/PBSA (kcal/mol)** |
| --- | --- | --- |
| **1** | 0010 | ‑54.58 |
| **2** | 0012 | ‑52.14 |
| **3** | 7JU7 | ‑51.61 |
| **4** | 7K6D | ‑50.68 |
| **5** | 0031 | ‑46.36 |
| **6** | 6LU7 | ‑40.41 |
| **7** | 7C8T | ‑39.34 |
| **8** | 7C8R | ‑36.49 |
| **9** | 0007 | ‑35.37 |
| **10** | 0026 | ‑34.99 |
| **11** | 0004 | ‑34.36 |
| **12** | 6XR3 | ‑33.93 |
| **13** | 0020 | ‑33.48 |
| **14** | 0037 | ‑33.45 |
| **15** | 6Y2F | ‑31.83 |
| **16** | 0027 | ‑31.65 |
| **17** | 0003 | ‑30.21 |
| **18** | 0036 | ‑30.18 |
| **19** | 0038 | ‑29.51 |
| **20** | 6XMK | ‑29.02 |
| **21** | 0013 | ‑28.09 |
| **22** | 0019 | ‑27.02 |
| **23** | 6W63 | ‑26.22 |
| **24** | 7K40 | ‑26.08 |
| **25** | 6WTT | ‑25.69 |
| **26** | 7JYC | ‑25.45 |
| **27** | 0006 | ‑24.60 |
| **28** | 6LZE | ‑24.55 |
| **29** | 0011 | ‑24.40 |

**Table S14. ΔG MM/PBSA for inhibitors from Table S11 with 1000 ns MD production excluding ligands with high movement on the active site.**

| **Ligand** | **ΔG MM/PBSA Protomer A (kJ/mol)** | **ΔG MM/PBSA Protomer B (kJ/mol)** |
| --- | --- | --- |
| 12 | -252.376 ± 25.872 | -252.338 ± 25.878 |
| 7JU7 | -280.077 ± 35.917 | -237.803 ± 19.333 |
| 7K6D | -230.567 ± 21.056 | -151.087 ± 23.12 |
| 30 | -208.846 ± 17.98 | -211.059 ± 16.62 |
| 6LU7 | -175.281 ± 18.814 | -184.308 ± 17.746 |
| 7C8R | -131.833 ± 19.619 | -176.2 ± 21.893 |
| 7C8T | -171.871 ± 19.231 | -171.6 ± 19.909 |
| 26 | -149.776 ± 17.363 | -162.631 ± 16.34 |
| 7 | -91.795 ± 29.451 | -161.345 ± 16.096 |
| 4 | -156.919 ± 15.06 | -158.447 ± 15.083 |
| 6XR3 | -117.476 ± 16.882 | -158.218 ± 17.094 |
| 35 | -159.21 ± 19.179 | -143.556 ± 22.533 |
| 6Y2F | -153.167 ± 18.068 | -150.399 ± 17.036 |
| 3 | -104.577 ± 18.938 | -145.175 ± 19.48 |
| 13 | -112.323 ± 22.519 | -142.127 ± 19.176 |
| 6XMK | -130.561 ± 15.519 | -118.836 ± 16.808 |
| 6W63 | -124.042 ± 13.909 | -125.148 ± 12.523 |
| 6 | -101.988 ± 22.349 | -127.055 ± 16.777 |
| 7JYC | -145.445 ± 25.567 | -125.971 ± 16.75 |
| 7K40 | -124.393 ± 17.694 | -71.654 ± 30.293 |
| 19 | -119.743 ± 26.738 | -131.483 ± 25.321 |
| 6WTK | -87.26 ± 23.398 | -121.161 ± 17.115 |
| 6LZE | -92.909 ± 20.148 | -117.696 ± 14.342 |

**Table S15. Second ΔG MM/PBSA ranking (affinity ranking) for inhibitors.**

| **Position** | **Ligand** | **ΔG MM/PBSA (kcal/mol)** |
| --- | --- | --- |
| **1** | **12** | -54.14 |
| **2** | **7JU7** | -52.22 |
| **3** | **7K6D** | -50.07 |
| **4** | **30** | -46.47 |
| **5** | **6LU7** | -39.81 |
| **6** | **7C8R** | -36.88 |
| **7** | **7C8T** | -36.48 |
| **8** | **26** | -34.96 |
| **9** | **7** | -34.72 |
| **10** | **4** | -34.26 |
| **11** | **6XR3** | -33.73 |
| **12** | **35** | -33.47 |
| **13** | **6Y2F** | -31.87 |
| **14** | **3** | -30.04 |
| **15** | **13** | -29.39 |
| **16** | **6XMK** | -27.50 |
| **17** | **6W63** | -26.92 |
| **18** | **6** | -26.36 |
| **19** | **7JYC** | -26.10 |
| **20** | **7K40** | -25.50 |
| **21** | **19** | -25.37 |
| **22** | **6WTK** | -24.87 |
| **23** | **6LZE** | -24.70 |

| 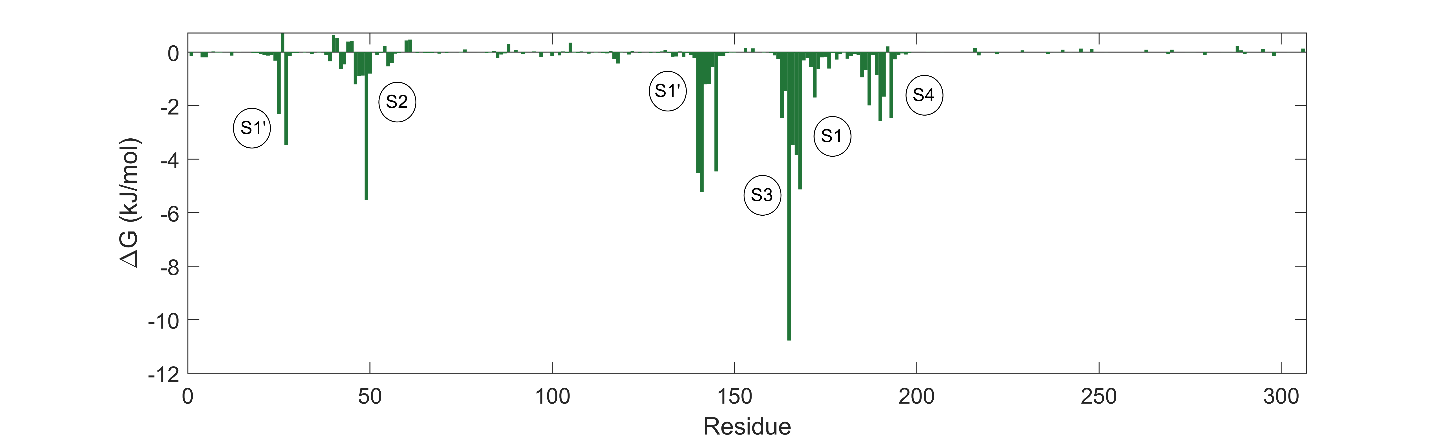  **(A)** |
| --- |
| 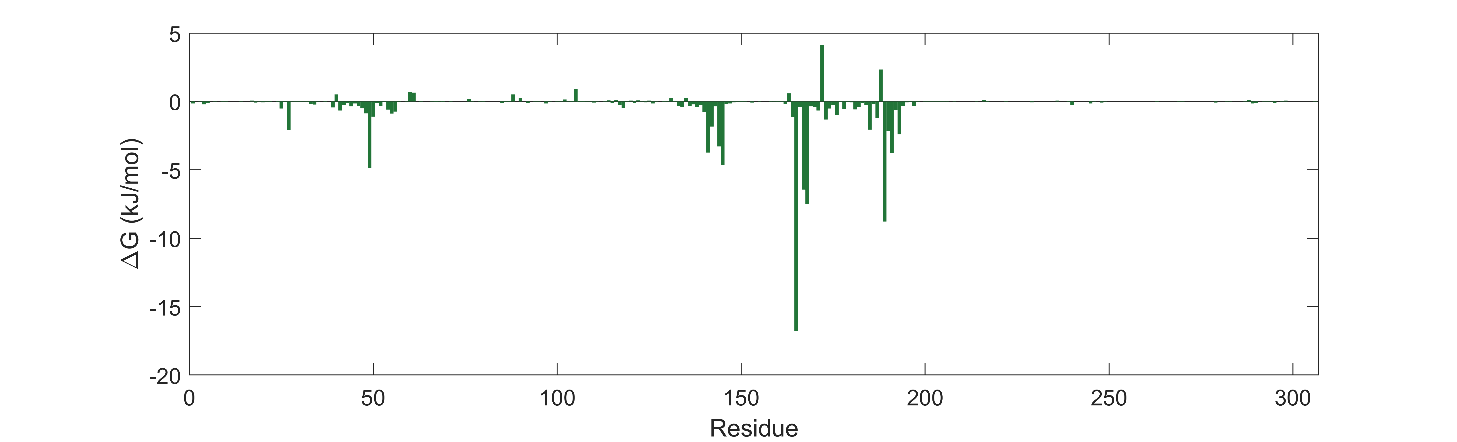  **(B)** |
| 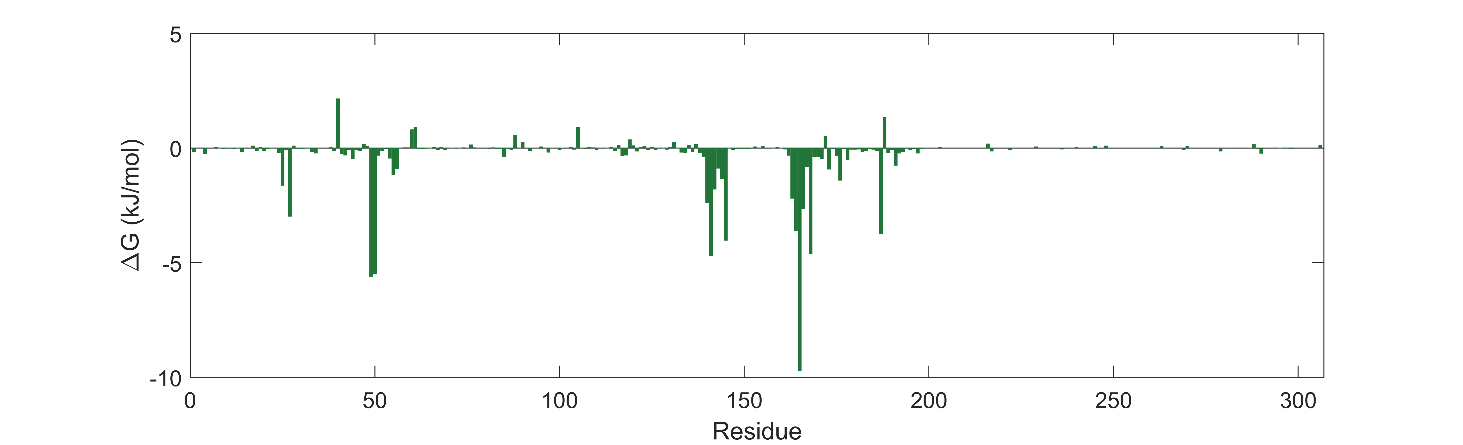  **(C)** |
| 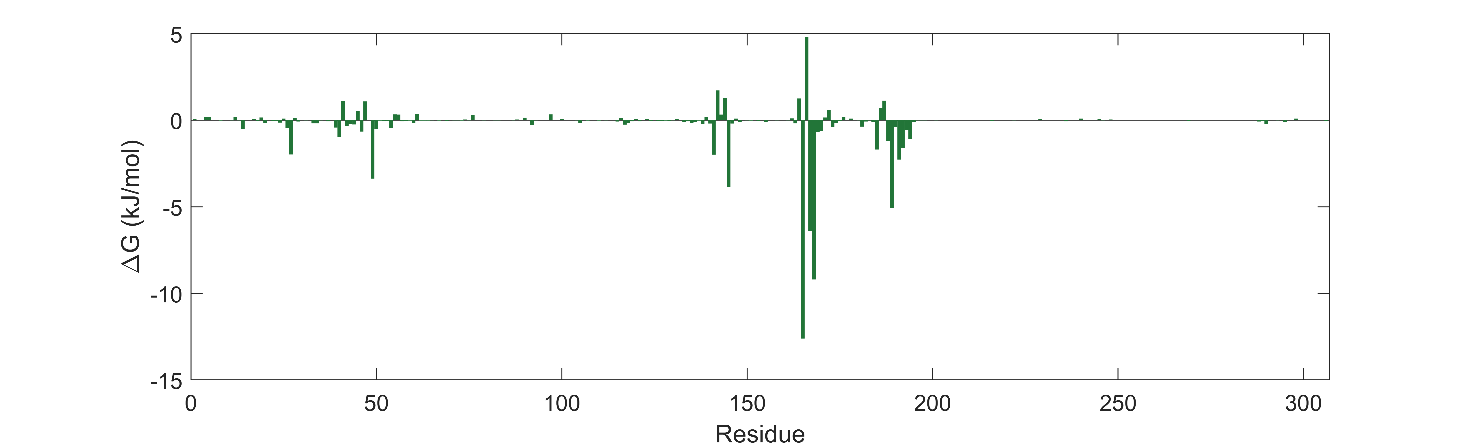  **(D)** |

**Figure S25. Residue contribution profile of protein-ligand binding energy for complexes (A) 6LU7, (B) 7K6D, (C) 6Y2F and (D) 0026.**

| 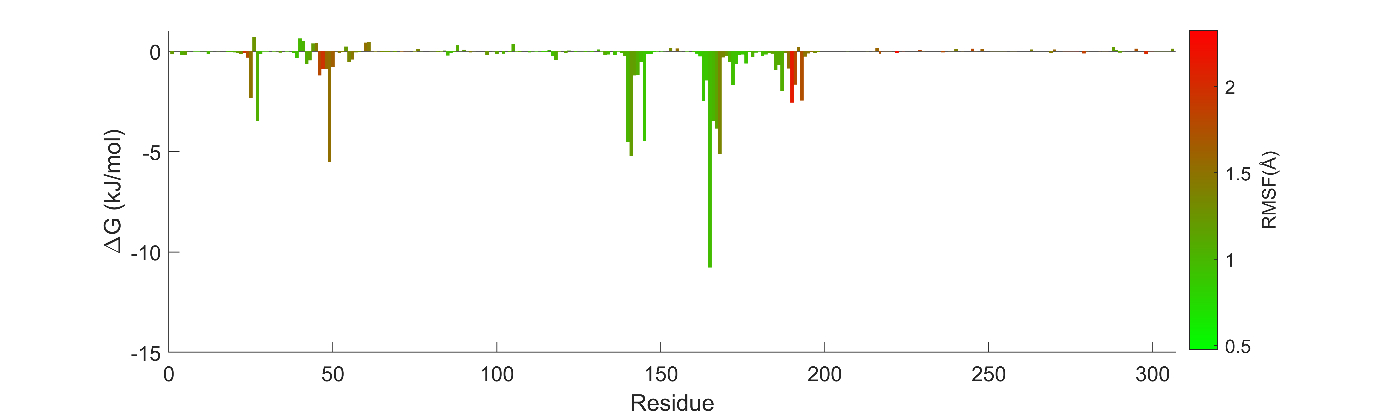 |
| --- |
| 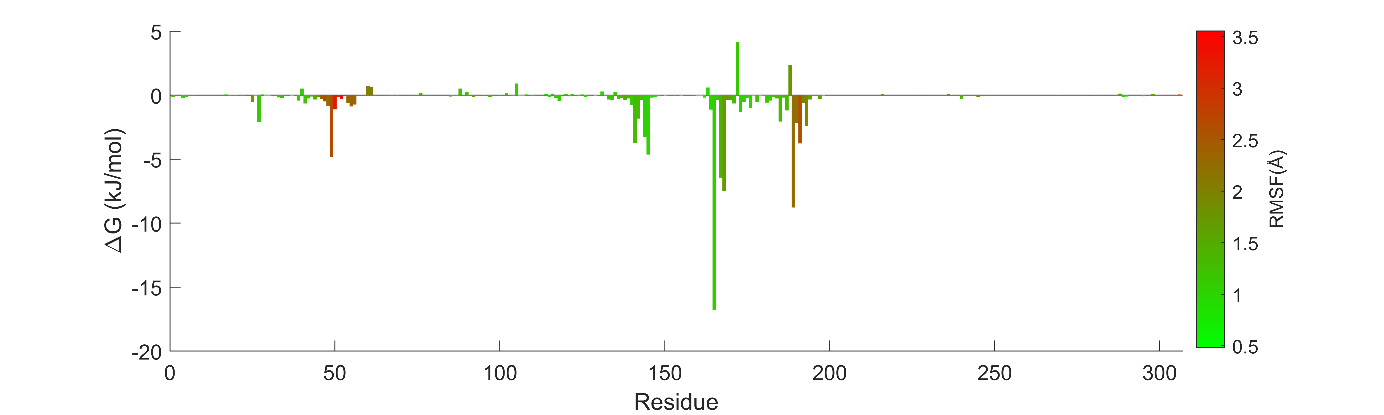  **(A)**  **(B)** |
| 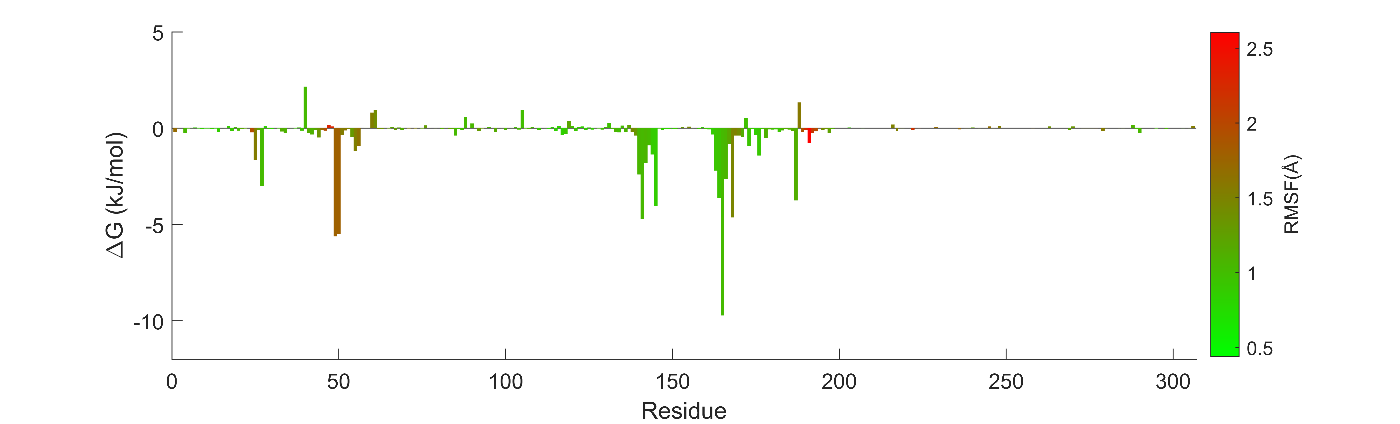 |
| 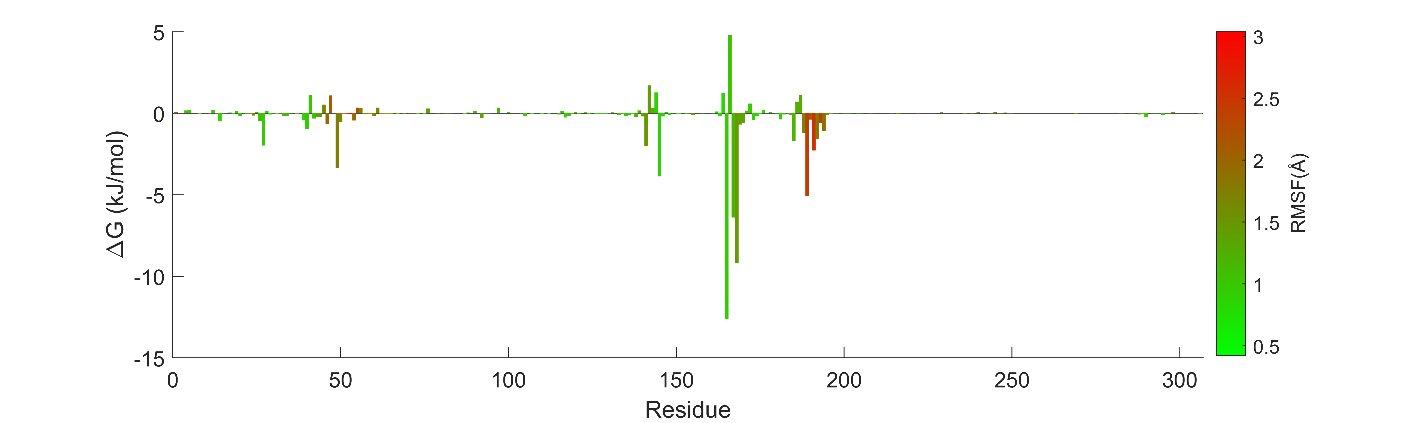  **(C)**  **(D)** |

**Figure S26. Residue contribution profile of protein-ligand binding energy and RMSF C-α values for complexes (A) 6LU7, (B) 7K6D, (C) 6Y2F and (D) 0026.**

| **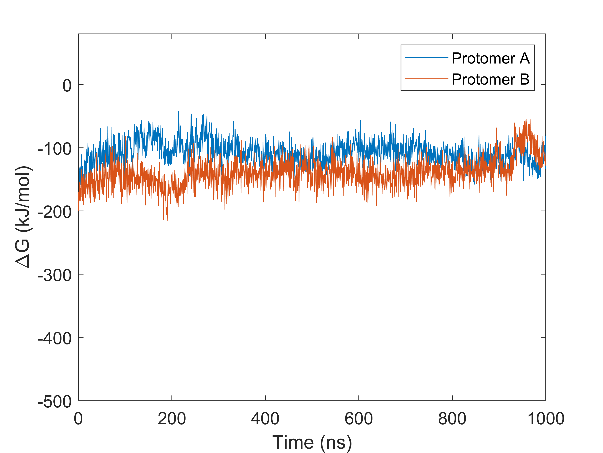** | **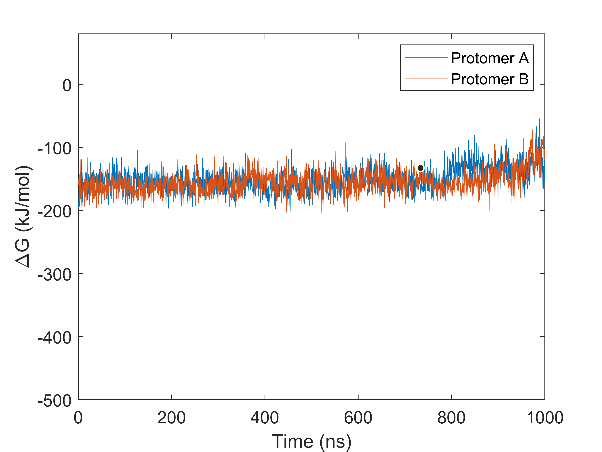** |
| --- | --- |
| **0003** | **0004** |
| **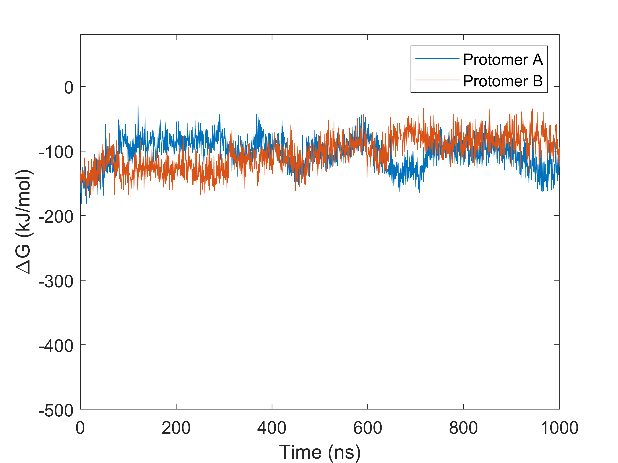** | **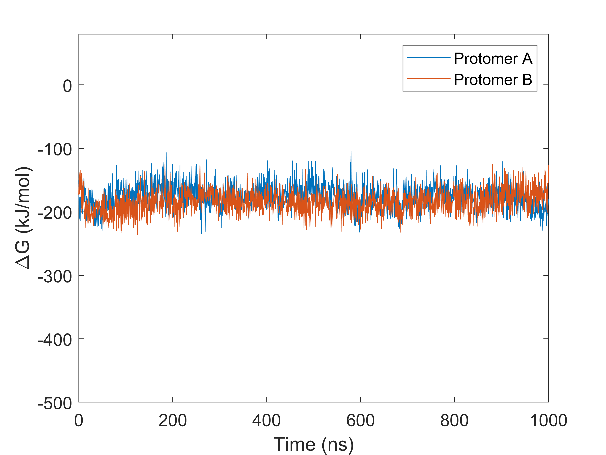** |
| **0006** | **6LU7** |
| **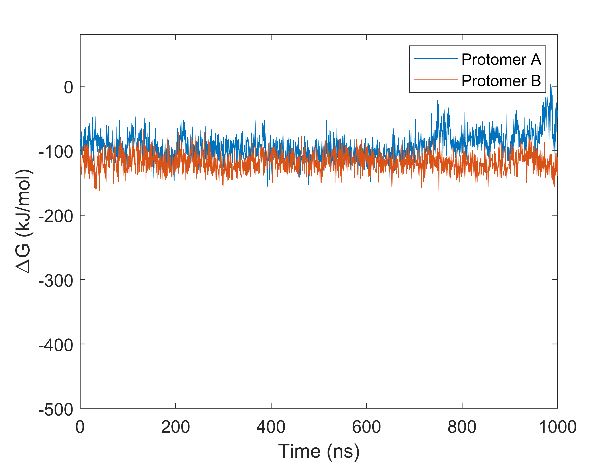** | **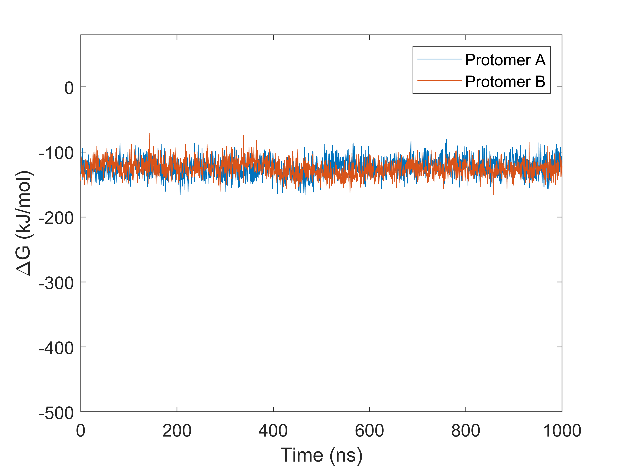** |
| **6LZE** | **6W63** |

**Figure S27. MM/PBSA ligand-protein binding energy vs MD simulation time for 0003, 0004, 0006, 6LU7, 6LZE and 6W63 complexes.**

| **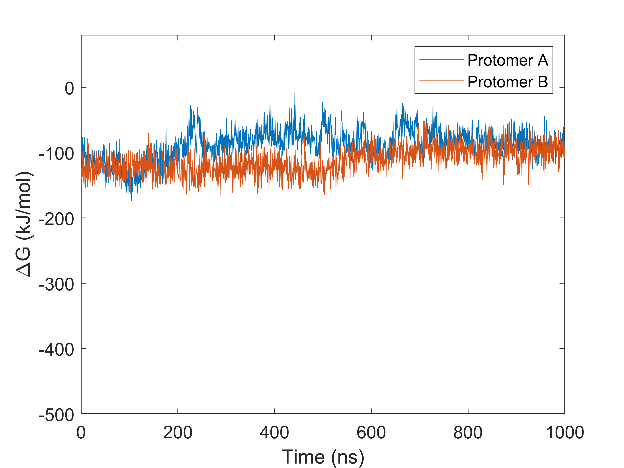** | **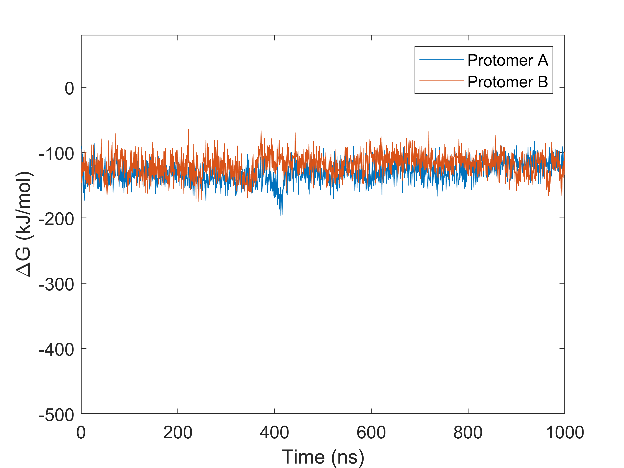** |
| --- | --- |
| **6WTK** | **6XMK** |
| **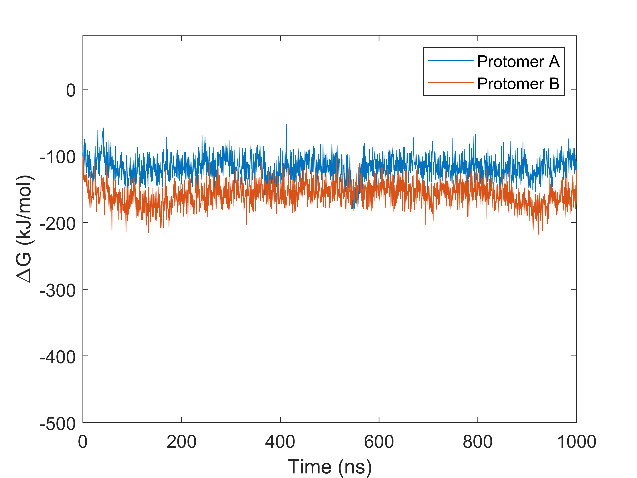** | **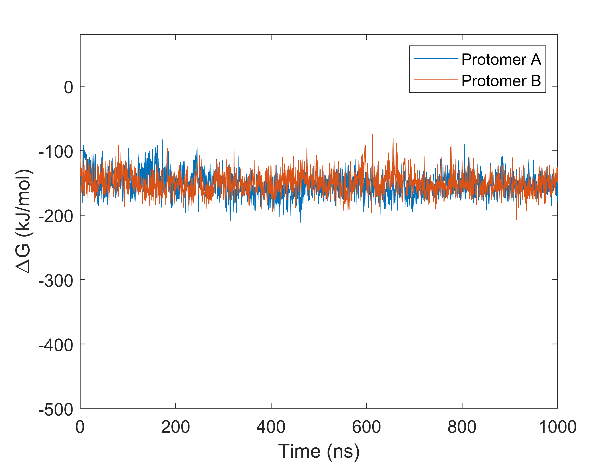** |
| **6XR3** | **6Y2F** |
| **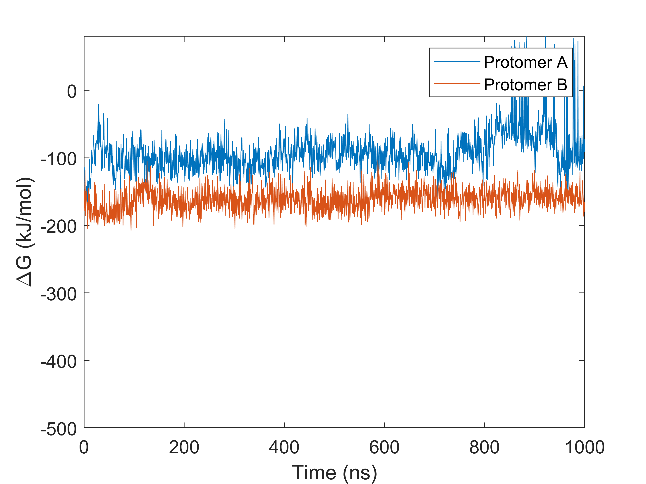** | **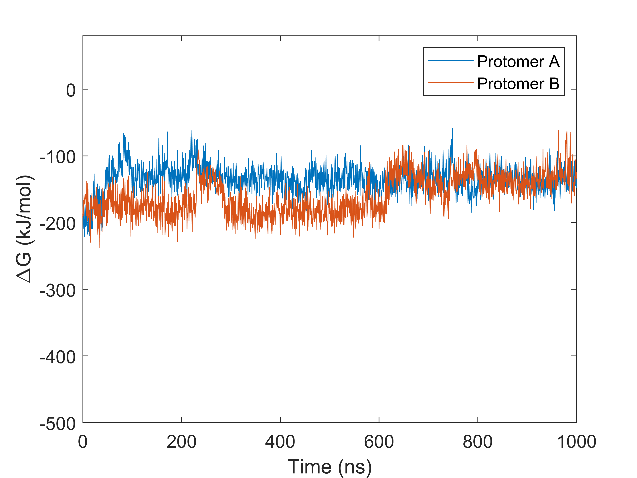** |
| **0007** | **7C8R** |

**Figure S28. MM/PBSA ligand-protein binding energy vs MD simulation time for 6WTK, 6XMK, 6XR3, 6Y2F, 0007 and 7C8R complexes.**

| **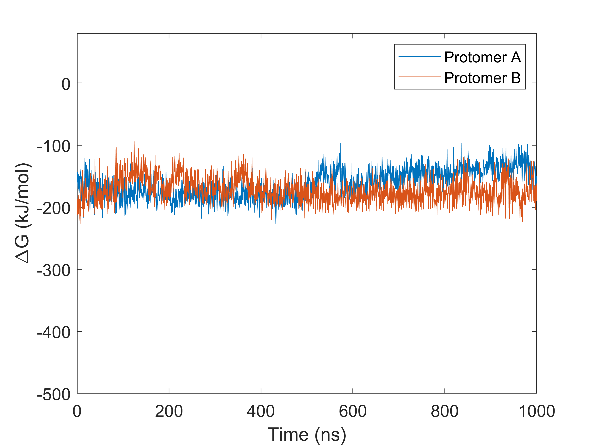** | **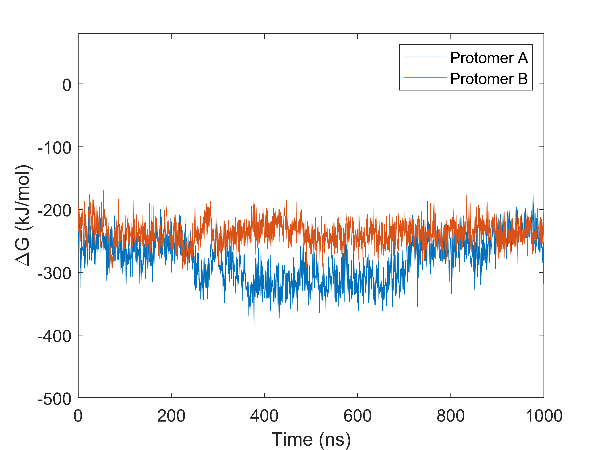** |
| --- | --- |
| **7C8T** | **7JU7** |
| **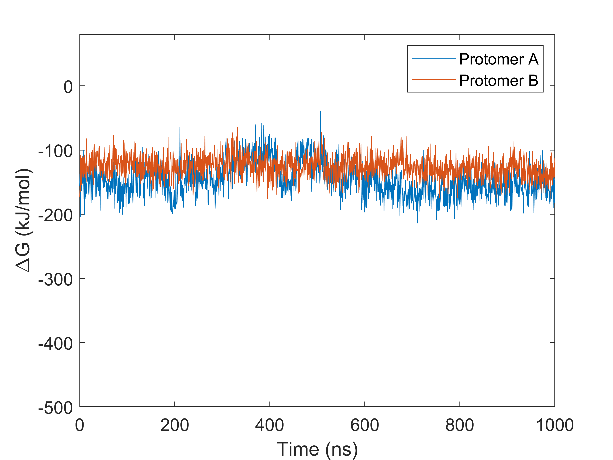** | **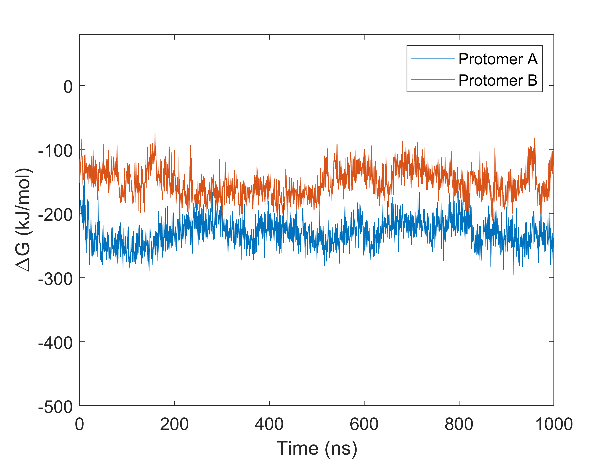** |
| **7JYC** | **7K6D** |
| **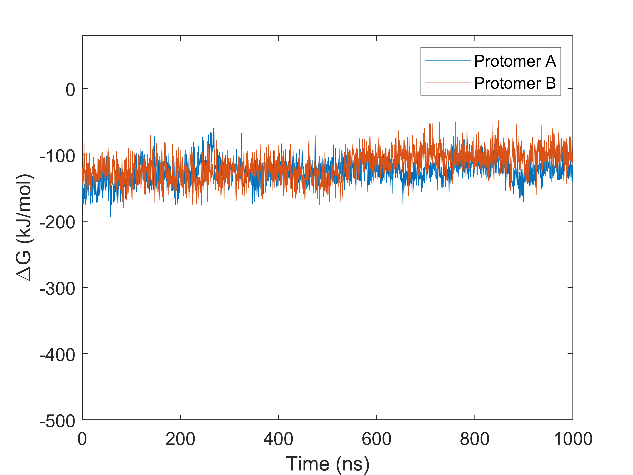** | **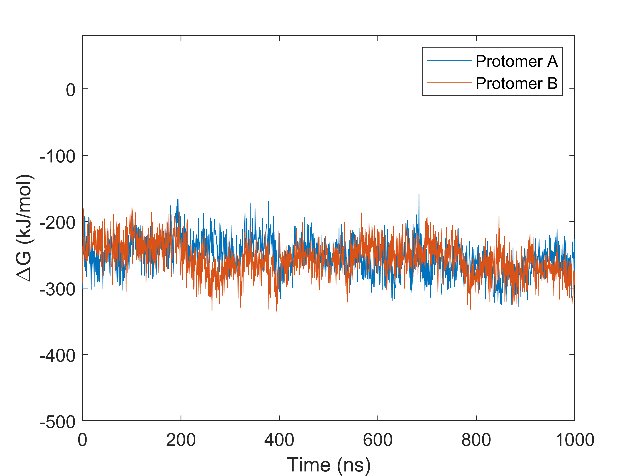** |
| **7K40** | **0012** |

**Figure S29. MM/PBSA ligand-protein binding energy vs MD simulation time for 7C8T, 7JU7, 7JYC, 7K6D, 7K40 and 0012 complexes.**

| **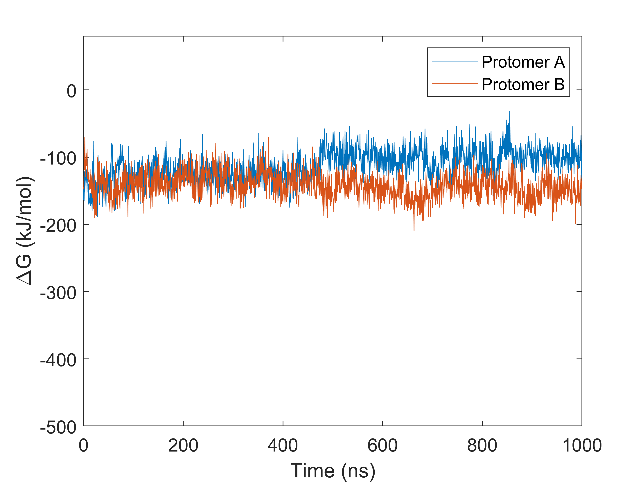** | **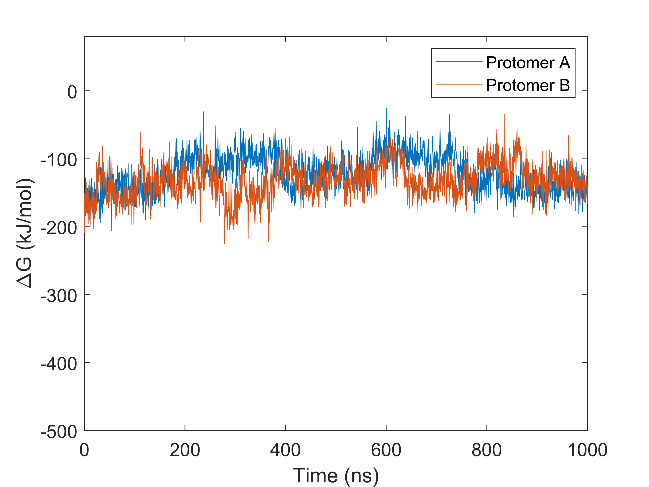** |
| --- | --- |
| **0013** | **0019** |
| **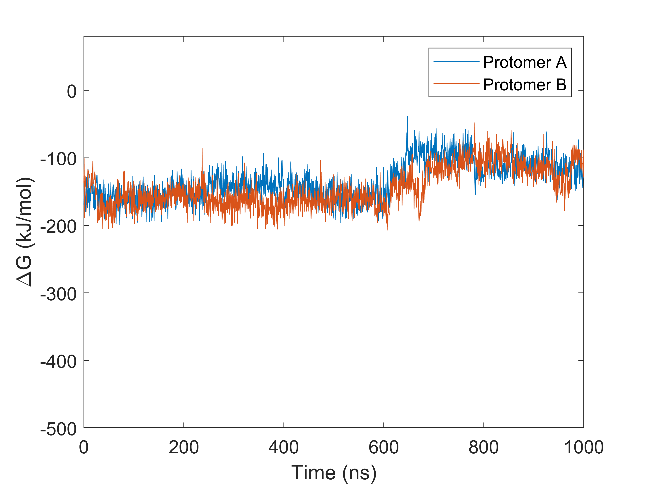** | **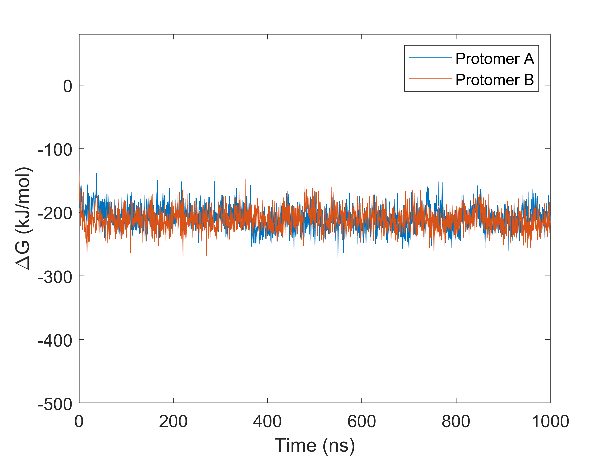** |
| **0026** | **0030** |
| **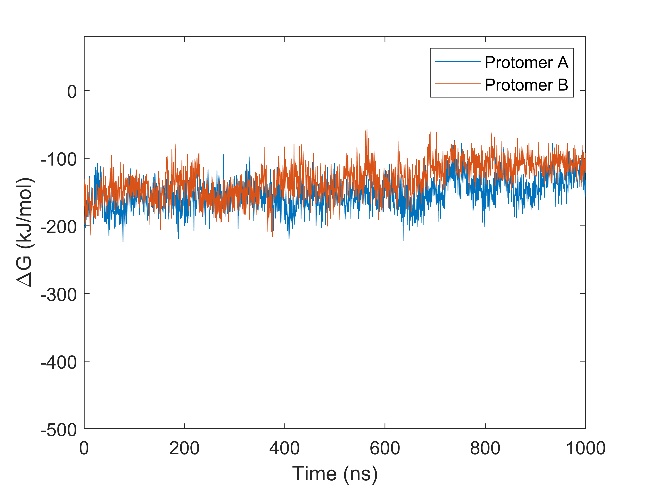** | |
| **0035** | |

**Figure S30. MM/PBSA ligand-protein binding energy vs MD simulation time for 0013, 0019, 0026, 0030 and 0035 complexes.**

| 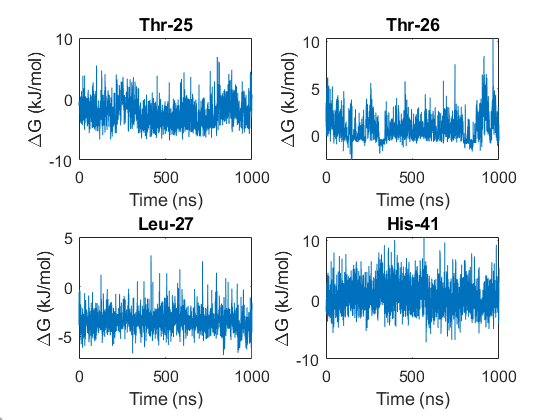 | 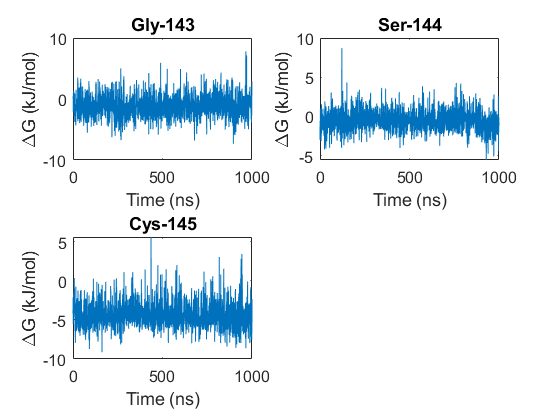 |
| --- | --- |
| 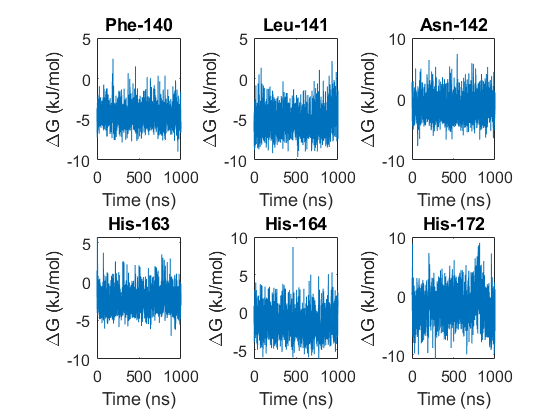 |  |
|  |  |

**Figure S31. MM/PBSA ligand-protein binding energy contributions of several active site residues vs MD simulation time for 6LU7 system.**

|  |  |
| --- | --- |
|  |  |
|  |  |

**Figure S32. MM/PBSA ligand-protein binding energy contributions of several active site residues vs MD simulation time for 6Y2F system.**

|  |  |
| --- | --- |
|  |  |
|  |  |

**Figure S33. MM/PBSA ligand-protein binding energy contributions of several active site residues vs MD simulation time for 7K6D system.**

|  |  |
| --- | --- |
|  |  |
|  |  |

**Figure S34. MM/PBSA ligand-protein binding energy contributions of several active site residues vs MD simulation time for 0026 system.**

| **Table S16. Number of ligand-pose-clusters obtained for each protein-ligand complex.** | **Table S17. Number of protein-ligand structures contained in each active-site-conformation-cluster (ASCC).** |
| --- | --- |
| \| **Complex** \| **Number of ligand-pose-clusters** \| \| --- \| --- \| \| **12** \| 8 \| \| **7JU7** \| 8 \| \| **7K6D** \| 8 \| \| **30** \| 8 \| \| **6LU7** \| 7 \| \| **7C8T** \| 8 \| \| **7C8R** \| 7 \| \| **7** \| 8 \| \| **26** \| 8 \| \| **4** \| 8 \| \| **6XR3** \| 8 \| \| **35** \| 8 \| \| **6Y2F** \| 8 \| \| **3** \| 8 \| \| **6XMK** \| 8 \| \| **13** \| 8 \| \| **19** \| 8 \| \| **6W63** \| 8 \| \| **7K40** \| 8 \| \| **6WTK** \| 8 \| \| **7JYC** \| 8 \| \| **6** \| 7 \| \| **6LZE** \| 8 \| | \| **ASCC** \| **Protein-ligand structures of each cluster** \| \| --- \| --- \| \| **1** \| 6 \| \| **2** \| 10 \| \| **3** \| 16 \| \| **4** \| 11 \| \| **5** \| 9 \| \| **6** \| 2 \| \| **7** \| 5 \| \| **8** \| 16 \| \| **9** \| 9 \| \| **10** \| 12 \| \| **11** \| 10 \| \| **12** \| 12 \| \| **13** \| 15 \| \| **14** \| 12 \| \| **15** \| 14 \| \| **16** \| 6 \| \| **17** \| 4 \| \| **18** \| 12 \| |

|  |  |
| --- | --- |
| **0003** | **0004** |
|  |  |
| **0006** | **6LU7** |

**Figure S35. Ligand-protein interaction diagrams with interaction frequency percentages of representative structures (Figure S5) for 0003, 0004, 0006 and 6LU7 complexes.** Representative structures from MD simulations were taken for each ligand and for these structures the present interactions were obtained using LigPlot+. The frequencies of occurrence of the interactions were calculated. Hydrophobic interactions are not necessarily located at the sites where the interaction takes place. HBA (red), HBD (green) and hydrophobic (yellow).

|  |  |
| --- | --- |
| **6LZE** | **6W63** |
|  |  |
| **6WTK** | **6XMK** |

**Figure S36. Ligand-protein interaction diagrams with interaction frequency percentages of representative structures (Figure S5) for 6LZE, 6W63, 6WTK and 6XMK complexes.** Representative structures from MD simulations were taken for each ligand and for these structures the present interactions were obtained using LigPlot+. The frequencies of occurrence of the interactions were calculated. Hydrophobic interactions are not necessarily located at the sites where the interaction takes place. HBA (red), HBD (green) and hydrophobic (yellow).

|  |  |
| --- | --- |
| **6XR3** | **6Y2F** |
|  |  |
| **0007** | **7C8R** |

**Figure S37. Ligand-protein interaction diagrams with interaction frequency percentages of representative structures (Figure S5) for 6XR3, 6Y2F, 0007 and 7C8R complexes.** Representative structures from MD simulations were taken for each ligand and for these structures the present interactions were obtained using LigPlot+. The frequencies of occurrence of the interactions were calculated. Hydrophobic interactions are not necessarily located at the sites where the interaction takes place. HBA (red), HBD (green) and hydrophobic (yellow).

|  |  |
| --- | --- |
| **7C8T** | **7JU7** |
|  |  |
| **7JYC** | **7K6D** |

**Figure S38. Ligand-protein interaction diagrams with interaction frequency percentages of representative structures (Figure S5) for 7C8T, 7JU7, 7JYC and 7K6D complexes.** Representative structures from MD simulations were taken for each ligand and for these structures the present interactions were obtained using LigPlot+. The frequencies of occurrence of the interactions were calculated. Hydrophobic interactions are not necessarily located at the sites where the interaction takes place. HBA (red), HBD (green) and hydrophobic (yellow).

|  |  |
| --- | --- |
| **7K40** | **0013** |
|  |  |
| **0019** | **0026** |

**Figure S39. Ligand-protein interaction diagrams with interaction frequency percentages of representative structures (Figure S5) for 7K40, 0013, 0019 and 0026 complexes.** Representative structures from MD simulations were taken for each ligand and for these structures the present interactions were obtained using LigPlot+. The frequencies of occurrence of the interactions were calculated. Hydrophobic interactions are not necessarily located at the sites where the interaction takes place. HBA (red), HBD (green) and hydrophobic (yellow).

|  |  |
| --- | --- |
| **0012** | **0035** |
|  | |
| **0030** | |

**Figure S40. Ligand-protein interaction diagrams with interaction frequency percentages of representative structures (Figure S5) for 0012, 0035 and 0030 complexes.** Representative structures from MD simulations were taken for each ligand and for these structures the present interactions were obtained using LigPlot+. The frequencies of occurrence of the interactions were calculated. Hydrophobic interactions are not necessarily located at the sites where the interaction takes place. HBA (red), HBD (green) and hydrophobic (yellow).

|  |  |
| --- | --- |
| **Hydrophobic Met-49, His-41 (6LZE)** | **Hydrophobic Met-49, Met-165 (6LU7)** |
|  | |
| **HBD/HBA Glu-166 (6LU7)** | |

**Figure S41. Ligand poses from MD simulations corresponding to interactions of crucial residues for S2/S3 subsite.**

|  |  |
| --- | --- |
| **Hydrophobic Leu-141 (6Y2F)** | **HBD/HBA Phe-140, His-163 (6Y2F)** |
|  |  |
| **HBD/HBA Asn-142 (0003)** | **HBD His-164 (6Y2F)** |

**Figure S42. Ligand poses from MD simulations corresponding to interactions of crucial residues for S1 subsite.**

|  |  |
| --- | --- |
| **HBA Gln-189 (0003)** | **HBD Gln-189 (0035)** |
|  |  |
| **HBA Gln-192 (0030)** | **HBD Gln-192 (0035)** |
|  |  |
| **HBA Thr-190 (0026)** | **HBD Thr-190 (6LU7)** |
|  | |
| **Hydrophobic Pro-168, Leu-167, Ala-191, Ala-193 (6LU7)** | |

**Figure S43. Ligand poses from MD simulations corresponding to interactions of crucial residues for S4 subsite.**

|  |  |
| --- | --- |
| **HBA Cys-145, Ser-144, Gly-143 (6XMK)** | **Hydrophobic His-41, Thr-25, Thr-26, Leu-27 (6Y2F)** |

**Figure S44. Ligand poses from MD simulations corresponding to interactions of crucial residues for S1’ subsite.**

**Figure S45. Subsite contribution profile of protein-ligand binding energy for complexes (A) 6LU7, (B) 7K6D, (C) 6Y2F and (D) 0026.** S4 hydrophobic (Leu-167, Pro-168, Ala-191 and Ala-193), S3/S4 HBA/HBD (Glu-166, Gln-189, Thr-190 and Gln-192), S2 (Met-49 and Met-165), S1 (Phe-140, Leu-141, Asn-142, His-163, His-164 and His-172), S1’ oxyanion hole (Gly-143, Ser-144, Cys-145), S1’ hydrophobic (Thr-25, Thr-26, Leu-27 and His-41).

**Table S18. Protein-ligand binding energy contribution values (kJ/mol) of important active site residues for complexes 6LU7, 7K6D, 6Y2F and 0026.**

| **Residues** | **6LU7** | **6Y2F** | **7K6D** | **26** |
| --- | --- | --- | --- | --- |
| **Thr-25** | -2.3 | -1.6 | -0.5 | 0.1 |
| **Thr-26** | 0.7 | -0.1 | 0.0 | -0.5 |
| **Leu-27** | -3.5 | -3.0 | -2.1 | -2.0 |
| **His-41** | 0.5 | -0.3 | -0.7 | 1.1 |
| **Gly-143** | -1.2 | -0.9 | -0.4 | 0.3 |
| **Ser-144** | -0.5 | -1.4 | -3.3 | 1.3 |
| **Cys-145** | -4.5 | -4.0 | -4.6 | -3.9 |
| **Phe-140** | -4.5 | -2.4 | -0.8 | -0.2 |
| **Leu-141** | -5.2 | -4.7 | -3.7 | -2.0 |
| **Asn-142** | -1.2 | -1.8 | -1.8 | 1.7 |
| **His-163** | -2.5 | -2.2 | 0.6 | -0.2 |
| **His-164** | -1.5 | -3.6 | -1.1 | 1.3 |
| **His-172** | -1.7 | 0.5 | 4.1 | 0.6 |
| **Met-49** | -5.5 | -5.6 | -4.9 | -3.4 |
| **Met-165** | -10.8 | -9.7 | -16.8 | -12.6 |
| **Glu-166** | -3.5 | -2.6 | -0.4 | 4.8 |
| **Gln-189** | -0.9 | -0.2 | -8.8 | -5.1 |
| **Thr-190** | -2.6 | -0.1 | -2.2 | -0.4 |
| **Gln-192** | 0.2 | -0.2 | -0.6 | -1.6 |
| **Leu-167** | -3.9 | -0.8 | -6.4 | -6.4 |
| **Pro-168** | -5.1 | -4.6 | -7.5 | -9.2 |
| **Ala-191** | -1.7 | -0.8 | -3.8 | -2.3 |
| **Ala-193** | -2.5 | -0.2 | -2.4 | -0.6 |

|  |  |
| --- | --- |
|  |  |
|  | |

**Figure S46. Protein-ligand binding energy contribution graphs (kJ/mol) of important active site residues for complexes 6LU7, 7K6D, 6Y2F and 0026.**

|  |  |  |  |
| --- | --- | --- | --- |
| 1 (6Y2F-6) | 2 (6W63-5) | 3 (0003-5) | 4 (6XMK-7) |
|  |  |  |  |
| 5 (7JU7-5) | 6 (6WTK-2) | 7 (0007-1) | 8 (0035-4) |
|  |  |  |  |
| 9 (6LU7-2) | 10 (7K6D-1) | 11 (7JYC-2) | 12 (6XR3-4) |
|  |  |  |  |
| 13 (7C8T-3) | 14 (0006-7) | 15 (0013-5) | 16 (0006-4) |
|  | |  | |
| 17 (0035-5) | | 18 (6LZE-3) | |

**Figure S47. Representative structure (centroid) for each active-site-conformation-cluster (18).**

|  |  |  |  |
| --- | --- | --- | --- |
| *1* | *2* | *3* | *4* |
|  |  |  |  |
| 5 | *6* | *7* | *8* |
|  |  |  |  |
| 9 | 10 | 11 | 12 |
|  |  |  |  |
| 13 | 14 | 15 | 16 |
|  | |  | |
| 17 | | 18 | |

**Figure S48. Overlapped pharmacophore models (181) for each active-site-conformation-cluster (18).**

|  |  |  |  |
| --- | --- | --- | --- |
| 1 **(4)** | 2 **(4)** | 3 **(3)** (NCM-4) | 4 **(5)** (NCM-1) |
|  |  |  |  |
| 5 **(6)** (NCM-2) | 6 **(2)** | 7 **(4)** (NCM-5) | 8 **(6)** |
|  |  |  |  |
| 9 **(4)** | 10 **(6)** | 11 **(7)** | 12 **(5)** |
|  |  |  |  |
| 13 **(5)** | 14 **(4)** | 15 **(7)** | 16 **(4)** |
|  | |  | |
| 17 **(6)** (NCM-3) | | 18 **(5)** | |

**Figure S49. Non-covalent pharmacophore models (NCM).** Number of optional features in blue. HBA (red), HBD (green), hydrophobic (yellow), residue bonding point (orange), and optional feature (transparent).

|  |  |  |  |
| --- | --- | --- | --- |
| 1 **(4)** (CM-3) | 2 **(4)** | 3 **(3)** (CM-4) | 4 **(5)** (CM-1) |
|  |  |  |  |
| 5 **(6)** (CM-5) | 6 **(2)** | 7 **(4)** (CM-2) | 8 **(6)** |
|  |  |  |  |
| 9 **(4)** | 10 **(6)** | 11 **(7)** | 12 **(5)** |
|  |  |  |  |
| 13 **(5)** | 14 **(4)** | 15 **(7)** | 16 **(4)** |
|  | |  | |
| 17 **(6)** | | 18 **(5)** | |

**Figure S50. Covalent pharmacophore models (CM).** Number of optional features in blue. HBA (red), HBD (green), hydrophobic (yellow), residue bonding point (orange), and optional feature (transparent).

|  |  |
| --- | --- |
| **NCM-4** | **NCM-1** |
|  |  |
| **NCM-2** | **NCM-5** |
|  | |
| **NCM-3** | |

**Figure S51. Best non-covalent pharmacophore models (NCM) with** **residues directly involved in the pharmacophoric features detected by LigandScout.** HBA (red), HBD (green), hydrophobic (yellow), residue bonding point (orange), and optional feature (*).

|  |  |
| --- | --- |
| **CM-3** | **CM-4** |
|  |  |
| **CM-1** | **CM-5** |
|  | |
| **CM-2** | |

**Figure S52. Best covalent pharmacophore models (CM) with** **residues directly involved in the pharmacophoric features detected by LigandScout.** HBA (red), HBD (green), hydrophobic (yellow), residue bonding point (orange), and optional feature (*).

**Table S19. First validation for Non-covalent pharmacophore models.** Active site conformation cluster (ASCC). True positive (TP). False positive (FP). Area under ROC curve (AUC), Balanced accuracy (BACC). 23 actives and 575 decoys.

| **ASCC** | **Max omitted features** | **TP** | **FP** | **AUC** | **BACC** |
| --- | --- | --- | --- | --- | --- |
| 5 | 1 | 14 | 178 | 0.64 | 0.65 |
| 3 | 1 | 14 | 206 | 0.63 | 0.63 |
| 7 | 1 | 7 | 38 | 0.62 | 0.62 |
| 17 | 1 | 20 | 374 | 0.65 | 0.61 |
| 4 | 1 | 19 | 353 | 0.67 | 0.61 |
| 9 | 1 | 12 | 178 | 0.62 | 0.61 |
| 8 | 2 | 8 | 81 | 0.61 | 0.60 |
| 10 | 1 | 7 | 81 | 0.58 | 0.58 |
| 1 | 1 | 20 | 414 | 0.58 | 0.57 |
| 10 | 2 | 15 | 292 | 0.60 | 0.57 |
| 7 | 2 | 16 | 322 | 0.58 | 0.57 |
| 14 | 2 | 18 | 403 | 0.61 | 0.54 |
| 7 | 3 | 23 | 543 | 0.57 | 0.53 |
| 8 | 3 | 12 | 268 | 0.57 | 0.53 |
| 3 | 2 | 21 | 493 | 0.43 | 0.53 |
| 4 | 2 | 23 | 544 | 0.70 | 0.53 |
| 12 | 1 | 11 | 249 | 0.51 | 0.52 |
| 17 | 2 | 23 | 562 | 0.45 | 0.51 |
| 6 | 1 | 23 | 564 | 0.54 | 0.51 |
| 1 | 2 | 23 | 566 | 0.62 | 0.51 |
| 9 | 3 | 23 | 567 | 0.49 | 0.51 |
| 16 | 5 | 23 | 574 | 0.57 | 0.50 |
| 16 | 4 | 21 | 524 | 0.60 | 0.50 |
| 6 | 2 | 23 | 575 | 0.60 | 0.50 |
| 11 | 1 | 18 | 459 | 0.56 | 0.49 |
| 11 | 2 | 22 | 562 | 0.49 | 0.49 |
| 15 | 4 | 19 | 489 | 0.52 | 0.49 |
| 15 | 5 | 20 | 516 | 0.47 | 0.49 |
| 2 | 1 | 19 | 494 | 0.49 | 0.48 |
| 12 | 2 | 19 | 501 | 0.50 | 0.48 |
| 13 | 4 | 13 | 353 | 0.44 | 0.48 |
| 18 | 5 | 10 | 280 | 0.47 | 0.47 |
| 2 | 2 | 21 | 557 | 0.50 | 0.47 |
| 9 | 2 | 18 | 483 | 0.51 | 0.47 |
| 13 | 5 | 17 | 459 | 0.41 | 0.47 |
| 14 | 3 | 19 | 517 | 0.58 | 0.46 |
| 5 | 2 | 17 | 475 | 0.53 | 0.46 |
| 18 | 4 | 15 | 429 | 0.49 | 0.45 |

**Table S20. First validation for Covalent pharmacophore models.** Active site conformation cluster (ASCC). True positive (TP). False positive (FP). Area under ROC curve (AUC), Balanced accuracy (BACC). 19 actives and 475 decoys.

| **ASCC** | **Max omitted features** | **TP** | **FP** | **AUC** | **BACC** |
| --- | --- | --- | --- | --- | --- |
| 4 | 1 | 19 | 146 | 0.91 | 0.85 |
| 7 | 2 | 16 | 115 | 0.80 | 0.80 |
| 1 | 1 | 16 | 172 | 0.79 | 0.74 |
| 3 | 1 | 9 | 35 | 0.71 | 0.70 |
| 5 | 1 | 9 | 49 | 0.69 | 0.69 |
| 3 | 2 | 16 | 231 | 0.66 | 0.68 |
| 7 | 1 | 7 | 7 | 0.68 | 0.68 |
| 8 | 3 | 11 | 126 | 0.67 | 0.66 |
| 17 | 1 | 12 | 154 | 0.67 | 0.65 |
| 16 | 4 | 19 | 337 | 0.79 | 0.65 |
| 10 | 2 | 11 | 139 | 0.66 | 0.64 |
| 5 | 2 | 15 | 264 | 0.68 | 0.62 |
| 9 | 1 | 6 | 46 | 0.62 | 0.61 |
| 6 | 1 | 19 | 376 | 0.58 | 0.60 |
| 14 | 2 | 13 | 240 | 0.66 | 0.59 |
| 7 | 3 | 18 | 374 | 0.76 | 0.58 |
| 8 | 2 | 4 | 27 | 0.58 | 0.58 |
| 12 | 1 | 7 | 103 | 0.58 | 0.58 |
| 9 | 2 | 15 | 310 | 0.71 | 0.57 |
| 4 | 2 | 19 | 410 | 0.81 | 0.57 |
| 2 | 1 | 13 | 268 | 0.63 | 0.56 |
| 17 | 2 | 19 | 432 | 0.58 | 0.55 |
| 1 | 2 | 18 | 429 | 0.61 | 0.52 |
| 11 | 1 | 16 | 381 | 0.58 | 0.52 |
| 10 | 1 | 2 | 32 | 0.52 | 0.52 |
| 16 | 5 | 19 | 459 | 0.66 | 0.52 |
| 6 | 2 | 19 | 471 | 0.61 | 0.50 |
| 12 | 2 | 14 | 353 | 0.52 | 0.50 |
| 9 | 3 | 18 | 455 | 0.68 | 0.49 |
| 2 | 2 | 16 | 417 | 0.63 | 0.48 |
| 13 | 4 | 13 | 353 | 0.44 | 0.47 |
| 14 | 3 | 14 | 382 | 0.63 | 0.47 |
| 15 | 4 | 13 | 365 | 0.57 | 0.46 |
| 13 | 5 | 12 | 344 | 0.51 | 0.45 |
| 18 | 4 | 2 | 109 | 0.45 | 0.44 |
| 11 | 2 | 16 | 463 | 0.53 | 0.43 |
| 15 | 5 | 13 | 399 | 0.52 | 0.42 |
| 18 | 5 | 5 | 239 | 0.39 | 0.38 |

**Table S21. Second validation for Non-covalent pharmacophore models.** Active site conformation cluster (ASCC). True positive (TP). False positive (FP). Area under ROC curve (AUC), Balanced accuracy (BACC).

| **NCM** | **ASCC** | **Max omitted features** | **TP** | **FP** | **AUC** | **BACC** |
| --- | --- | --- | --- | --- | --- | --- |
| NCM-1 | 4 | 1 | 17 | 133 | 0.75 | 0.75 |
| NCM-2 | 5 | 1 | 15 | 153 | 0.70 | 0.69 |
| NCM-3 | 17 | 1 | 20 | 374 | 0.65 | 0.61 |
| NCM-4 | 3 | 1 | 15 | 218 | 0.64 | 0.64 |
| NCM-5 | 7 | 1 | 7 | 25 | 0.63 | 0.63 |

**Table S22. Second validation for Covalent pharmacophore models.** Active site conformation cluster (ASCC). True positive (TP). False positive (FP). Area under ROC curve (AUC), Balanced accuracy (BACC).

| **CM** | **ASCC** | **Max omitted features** | **TP** | **FP** | **AUC** | **BACC** |
| --- | --- | --- | --- | --- | --- | --- |
| CM-1 | 4 | 1 | 19 | 131 | 0.93 | 0.86 |
| CM-2 | 7 | 2 | 15 | 85 | 0.83 | 0.81 |
| CM-3 | 1 | 1 | 15 | 111 | 0.80 | 0.77 |
| CM-4 | 3 | 1 | 9 | 31 | 0.71 | 0.70 |
| CM-5 | 5 | 1 | 9 | 40 | 0.70 | 0.69 |

**Table S23. Performance of Non-covalent pharmacophore models with disabled optional features.** Active site conformation cluster (ASCC). True positive (TP). False positive (FP). Area under ROC curve (AUC), Balanced accuracy (BACC).

| **NCM** | **ASCC** | **TP** | **FP** | **AUC** | **BACC** |
| --- | --- | --- | --- | --- | --- |
| NCM-1 | 4 | 1 | 4 | 0.52 | 0.51 |
| NCM-2 | 5 | 1 | 1 | 0.50 | 0.52 |
| NCM-3 | 17 | 0 | 8 | 0.49 | 0.49 |
| NCM-4 | 3 | 0 | 8 | 0.49 | 0.49 |
| NCM-5 | 7 | 0 | 0 | 0.00 | 0.50 |

**Table S24. Performance of covalent pharmacophore models with disabled optional features.** Active site conformation cluster (ASCC). True positive (TP). False positive (FP). Area under ROC curve (AUC), Balanced accuracy (BACC).

| **CM** | **ASCC** | **TP** | **FP** | **AUC** | **BACC** |
| --- | --- | --- | --- | --- | --- |
| CM-1 | 4 | 4 | 5 | 0.60 | 0.60 |
| CM-2 | 7 | 0 | 0 | 0.00 | 0.50 |
| CM-3 | 1 | 2 | 1 | 0.55 | 0.55 |
| CM-4 | 3 | 0 | 0 | 0.00 | 0.50 |
| CM-5 | 5 | 1 | 0 | 0.50 | 0.52 |

**Table S25. Performance of Non-covalent pharmacophore models with disabled oxyanion feature.** Active site conformation cluster (ASCC). True positive (TP). False positive (FP). Area under ROC curve (AUC), Balanced accuracy (BACC).

| **NCM** | **ASCC** | **Max omitted features** | **TP** | **FP** | **AUC** | **BACC** |
| --- | --- | --- | --- | --- | --- | --- |
| NCM-1 | 4 | 1 | 18 | 241 | 0.69 | 0.68 |
| NCM-2 | 5 | 1 | 14 | 295 | 0.57 | 0.54 |
| NCM-3 | 17 | 1 | 21 | 504 | 0.52 | 0.52 |
| NCM-4 | 3 | 1 | 12 | 225 | 0.56 | 0.56 |
| NCM-5 | 7 | 1 | 6 | 58 | 0.58 | 0.58 |

**Table S26. Performance of covalent pharmacophore models with disabled oxyanion feature.** Active site conformation cluster (ASCC). True positive (TP). False positive (FP). Area under ROC curve (AUC), Balanced accuracy (BACC).

| **CM** | **ASCC** | **Max omitted features** | **TP** | **FP** | **AUC** | **BACC** |
| --- | --- | --- | --- | --- | --- | --- |
| CM-1 | 4 | 1 | 19 | 371 | 0.77 | 0.61 |
| CM-2 | 7 | 2 | 16 | 331 | 0.63 | 0.57 |
| CM-3 | 1 | 1 | 16 | 365 | 0.65 | 0.53 |
| CM-4 | 3 | 1 | 10 | 186 | 0.56 | 0.56 |
| CM-5 | 5 | 1 | 14 | 241 | 0.64 | 0.61 |

**Table S27. Virtual screening results for Nirmatrelvir using Covalent pharmacophore models.**

| **CM** | **ASCC** | **Max omitted features** | **Scoring function** |
| --- | --- | --- | --- |
| CM-1 | 4 | 1 | 102.13 |
| CM-2 | 7 | 2 | 84.85 |
| CM-3 | 1 | 1 | 80.98 |
| CM-4 | 3 | 1 | 74.97 |
| CM-5 | 5 | 1 | 73.86 |

**Table S28. Virtual screening results for Nirmatrelvir using Non-covalent pharmacophore models.**

| **NCM** | **ASCC** | **Max omitted features** | **Scoring function** |
| --- | --- | --- | --- |
| NCM-1 | 4 | 1 | 73.17 |
| NCM-2 | 5 | 1 | 72.77 |
| NCM-3 | 17 | 1 | 81.81 |
| NCM-4 | 3 | 1 | 72.46 |
| NCM-5 | 7 | 1 | 101.31 |

**REFERENCES**

1. Jin, Z. *et al.* Structure of Mpro from SARS-CoV-2 and discovery of its inhibitors. *Nature* **582**, 289–293 (2020).

2. Dai, W. *et al.* Structure-based design of antiviral drug candidates targeting the SARS-CoV-2 main protease. *Science (80-. ).* **368**, 1331–1335 (2020).

3. Su, H. X. *et al.* Anti-SARS-CoV-2 activities in vitro of Shuanghuanglian preparations and bioactive ingredients. *Acta Pharmacol. Sin.* **41**, 1167–1177 (2020).

4. Ma, C. *et al.* Boceprevir, GC-376, and calpain inhibitors II, XII inhibit SARS-CoV-2 viral replication by targeting the viral main protease. *Cell Res.* **30**, 578–692 (2020).

5. Rathnayake, A. D. *et al.* 3C-like protease inhibitors block coronavirus replication in vitro and improve survival in MERS-CoV–infected mice. *Sci. Transl. Med.* **12**, 1–11 (2020).

6. Thanigaimalai, P. *et al.* Development of potent dipeptide-type SARS-CoV 3CL protease inhibitors with novel P3 scaffolds: Design, synthesis, biological evaluation, and docking studies. *Eur. J. Med. Chem.* **68**, 372–384 (2013).

7. Zhang, L. *et al.* Crystal structure of SARS-CoV-2 main protease provides a basis for design of improved a-ketoamide inhibitors. *Science (80-. ).* **368**, 409–412 (2020).

8. Yang, S. *et al.* Synthesis, crystal structure, structure-activity relationships, and antiviral activity of a potent SARS coronavirus 3CL protease inhibitor. *J. Med. Chem.* **49**, 4971–4980 (2006).

9. Drayman, N. *et al.* Drug repurposing screen identifies masitinib as a 3CLpro inhibitor that blocks replication of SARS-CoV-2 in vitro. *Preprint* (2020) doi:10.1101/2020.08.31.274639.

10. Anson, B. *et al.* Broad-spectrum inhibition of coronavirus main and papain-like proteases by HCV drugs. *Preprint* (2020) doi:10.21203/rs.3.rs-26344/v1.

11. Liu, X. *et al.* Potential therapeutic effects of dipyridamole in the severely ill patients with COVID-19. *Acta Pharm. Sin. B* **10**, 1205–1215 (2020).

12. Yamamoto, N., Matsuyama, S., Hoshino, T. & Yamamoto, N. Nelfinavir inhibits replication of severe acute respiratory syndrome coronavirus 2 in vitro. *Preprint* 2020.04.06.026476 (2020) doi:10.1101/2020.04.06.026476.

13. Jo, S., Kim, S., Shin, D. H. & Kim, M. S. Inhibition of SARS-CoV 3CL protease by flavonoids. *J. Enzyme Inhib. Med. Chem.* **35**, 145–151 (2020).

14. Turlington, M. *et al.* Discovery of N-(benzo[1,2,3]triazol-1-yl)-N-(benzyl)acetamido)phenyl) carboxamides as severe acute respiratory syndrome coronavirus (SARS-CoV) 3CLpro inhibitors: Identification of ML300 and noncovalent nanomolar inhibitors with an induced-fit binding. *Bioorganic Med. Chem. Lett.* **23**, 6172–6177 (2013).

15. Akaji, K. *et al.* Structure-based design, synthesis, and evaluation of peptide-mimetic SARS 3CL protease inhibitors. *J. Med. Chem.* **54**, 7962–7973 (2011).

16. Shao, Y. M. *et al.* Structure-based design and synthesis of highly potent SARS-CoV 3CL protease inhibitors. *ChemBioChem* **8**, 1654–1657 (2007).

17. Lu, I. L. *et al.* Structure-based drug design and structural biology study of novel nonpeptide inhibitors of severe acute respiratory syndrome coronavirus main protease. *J. Med. Chem.* **49**, 5154–5161 (2006).

18. Ryu, Y. B. *et al.* SARS-CoV 3CLpro inhibitory effects of quinone-methide triterpenes from Tripterygium regelii. *Bioorganic Med. Chem. Lett.* **20**, 1873–1876 (2010).

19. Chen, L. R. *et al.* Synthesis and evaluation of isatin derivatives as effective SARS coronavirus 3CL protease inhibitors. *Bioorganic Med. Chem. Lett.* **15**, 3058–3062 (2005).

20. Regnier, T. *et al.* New developments for the design, synthesis and biological evaluation of potent SARS-CoV 3CLpro inhibitors. *Bioorganic Med. Chem. Lett.* **19**, 2722–2727 (2009).

21. Ramajayam, R., Tan, K. P., Liu, H. G. & Liang, P. H. Synthesis and evaluation of pyrazolone compounds as SARS-coronavirus 3C-like protease inhibitors. *Bioorganic Med. Chem.* **18**, 7849–7854 (2010).

22. Zhang, H. Z. *et al.* Design and synthesis of dipeptidyl glutaminyl fluoromethyl ketones as potent severe acute respiratory syndrome coronovirus (SARS-CoV) inhibitors. *J. Med. Chem.* **49**, 1198–1201 (2006).

23. Yang, H. *et al.* Design of wide-spectrum inhibitors targeting coronavirus main proteases. *PLoS Biol.* **3**, 1–11 (2005).

24. Zhu, L. *et al.* Peptide aldehyde inhibitors challenge the substrate specificity of the SARS-coronavirus main protease. *Antiviral Res.* **92**, 204–212 (2011).

25. Wen, C. C. *et al.* Specific plant terpenoids and lignoids possess potent antiviral activities against severe acute respiratory syndrome coronavirus. *J. Med. Chem.* **50**, 4087–4095 (2007).

26. Shao, Y. M. *et al.* Design, synthesis, and evaluation of trifluoromethyl ketones as inhibitors of SARS-CoV 3CL protease. *Bioorganic Med. Chem.* **16**, 4652–4660 (2008).

27. Ghosh, A. K. *et al.* Design and synthesis of peptidomimetic severe acute respiratory syndrome chymotrypsin-like protease inhibitors. *J. Med. Chem.* **48**, 6767–6771 (2005).

28. Tian, W., Chen, C., Lei, X., Zhao, J. & Liang, J. CASTp 3.0: computed atlas of surface topography of proteins. *Nucleic Acids Res.* **46**, W363–W367 (2018).

29. Halgren, T. A. Identifying and Characterizing Binding Sites and Assessing Druggability. *J. Chem. Inf. Model.* **49**, 377–389 (2009).

30. RA, L. SURFNET: a program for visualizing molecular surfaces, cavities, and intermolecular interactions. *J. Mol. Graph.* **13**, 323–330 (1995).

31. Saranya, N. & Selvaraj, S. Variation of protein binding cavity volume and ligand volume in protein–ligand complexes. *Bioorg. Med. Chem. Lett.* **19**, 5769–5772 (2009).

32. Anderson, A. C., O’Neil, R. H., Surti, T. S. & Stroud, R. M. Approaches to solving the rigid receptor problem by identifying a minimal set of flexible residues during ligand docking. *Chem. Biol.* **8**, 445–457 (2001).

33. Anand, K. *et al.* Structure of coronavirus main proteinase reveals combination of a chymotrypsin fold with an extra α-helical domain. *EMBO J.* **21**, 3213–3224 (2002).

34. Yang, H. *et al.* The crystal structures of severe acute respiratory syndrome virus main protease and its complex with an inhibitor. *Proc. Natl. Acad. Sci. U. S. A.* **100**, 13190–13195 (2003).

35. Suárez, D. & Díaz, N. SARS-CoV-2 Main Protease: A Molecular Dynamics Study. *J. Chem. Inf. Model.* **60**, 5815–5831 (2020).
